# Biofeedback and Digitalized Motivational Interviewing to Increase Daily Physical Activity: Series of Factorial N-of-1 Randomized Controlled Trials Piloting the Precious App

**DOI:** 10.2196/34232

**Published:** 2023-11-23

**Authors:** Johanna Nurmi, Keegan Knittle, Felix Naughton, Stephen Sutton, Todor Ginchev, Fida Khattak, Carmina Castellano-Tejedor, Pilar Lusilla-Palacios, Niklas Ravaja, Ari Haukkala

**Affiliations:** 1 Social Psychology Faculty of Social Sciences University of Helsinki Helsinki Finland; 2 Behavioural Science Group, Primary Care Unit Department of Public Health and Primary Care University of Cambridge Cambridge United Kingdom; 3 Faculty of Sport and Health Sciences University of Jyväskylä Jyväskylä Finland; 4 Behavioural and Implementation Science Group School of Health Sciences University of East Anglia Norwich United Kingdom; 5 Department of Communications and Networking Aalto University Espoo Finland; 6 Grupo de Investigación en Estrés y Salud Basic Psychology Department Autonomous University of Barcelona Barcelona Spain; 7 Research Group on Aging, Frailty and Care Transitions in Barcelona Parc Sanitari Pere Virgili & Vall d’Hebron Research Institute Barcelona Spain; 8 Psiquiatría Salud Mental y Adicciones Vall d’Hebron Institut de Recerca Barcelona Spain; 9 Servicio de Psiquiatría Hospital Universitari Vall d’Hebron Barcelona Spain; 10 Departament de Psiquiatria i Medicina Legal Universitat Autònoma de Barcelona Barcelona Spain; 11 Centro de Investigación Biomédica en Red de Salud Mental Instituto de Salud Carlos III Barcelona Spain; 12 Department of Psychology and Logopedics Faculty of Medicine University of Helsinki Helsinki Finland; 13 Helsinki Collegium for Advanced Studies University of Helsinki Helsinki Finland

**Keywords:** smartphone, daily steps, activity tracker, activity bracelet, motivational interviewing, self-efficacy, self-regulation, biofeedback, N-of-1, automated, digitalized, behavior change, intervention, ecological momentary assessment, within-person design, intensive longitudinal multilevel modeling, mobile phone

## Abstract

**Background:**

Insufficient physical activity is a public health concern. New technologies may improve physical activity levels and enable the identification of its predictors with high accuracy. The Precious smartphone app was developed to investigate the effect of specific modular intervention elements on physical activity and examine theory-based predictors within individuals.

**Objective:**

This study pilot-tested a fully automated factorial N-of-1 randomized controlled trial (RCT) with the Precious app and examined whether digitalized motivational interviewing (dMI) and heart rate variability–based biofeedback features increased objectively recorded steps. The secondary aim was to assess whether daily self-efficacy and motivation predicted within-person variability in daily steps.

**Methods:**

In total, 15 adults recruited from newspaper advertisements participated in a 40-day factorial N-of-1 RCT. They installed 2 study apps on their phones: one to receive intervention elements and one to collect ecological momentary assessment (EMA) data on self-efficacy, motivation, perceived barriers, pain, and illness. Steps were tracked using Xiaomi Mi Band activity bracelets. The factorial design included seven 2-day biofeedback interventions with a Firstbeat Bodyguard 2 (Firstbeat Technologies Ltd) heart rate variability sensor, seven 2-day dMI interventions, a wash-out day after each intervention, and 11 control days. EMA questions were sent twice per day. The effects of self-efficacy, motivation, and the interventions on subsequent steps were analyzed using within-person dynamic regression models and aggregated data using longitudinal multilevel modeling (level 1: daily observations; level 2: participants). The analyses were adjusted for covariates (ie, within- and between-person perceived barriers, pain or illness, time trends, and recurring events).

**Results:**

All participants completed the study, and adherence to activity bracelets and EMA measurements was high. The implementation of the factorial design was successful, with the dMI features used, on average, 5.1 (SD 1.0) times of the 7 available interventions. Biofeedback interventions were used, on average, 5.7 (SD 1.4) times out of 7, although 3 participants used this feature a day later than suggested and 1 did not use it at all. Neither within- nor between-person analyses revealed significant intervention effects on step counts. Self-efficacy predicted steps in 27% (4/15) of the participants. Motivation predicted steps in 20% (3/15) of the participants. Aggregated data showed significant group-level effects of day-level self-efficacy (*B=*0.462; *P*<.001), motivation (*B=*0.390; *P*<.001), and pain or illness (*B*=−1524; *P<*.001) on daily steps.

**Conclusions:**

The automated factorial N-of-1 trial with the Precious app was mostly feasible and acceptable, especially the automated delivery of the dMI components, whereas self-conducted biofeedback measurements were more difficult to time correctly. The findings suggest that changes in self-efficacy and motivation may have same-day effects on physical activity, but the effects vary across individuals. This study provides recommendations based on the lessons learned on the implementation of factorial N-of-1 RCTs.

## Introduction

### Background

Most adults do not engage in sufficient physical activity for good health [1]. Noncommunicable diseases related to sedentary lifestyles are one of the leading causes of death worldwide, costing societies approximately US $67.5 billion per year according to conservative estimates [2]. Although the core reasons and dynamics of insufficient physical activity vary among societies, it is a global public health priority to support individuals in increasing their physical activity levels and improving their health [3]. To design effective interventions, a better understanding is needed of the factors that determine individuals’ physical activity in everyday life and the techniques that are effective in targeting those determinants [4].

### The Promise of Physical Activity Apps

Smartphone apps and activity trackers offer many key features for supporting active lifestyles, and the wide reach and cost-effective dissemination of digital interventions hold promise for population-level behavioral support [5]. Sensor technology in smartphones provides many advantages for physical activity interventions, such as the automatic measurement of activity and individually tailored support messages [6,7]. Another benefit is the opportunity to collect real-time data on the association between individuals’ cognition and behavior in their natural environments, minimizing mistakes because of memory bias.

The effectiveness of mobile device–based interventions for physical activity varies, but overall, mobile health apps have led to small increases in physical activity [8,9] and decreases in sedentary time [10]. However, smartphone intervention effects on daily steps have not been demonstrated [11]. To develop smartphone interventions that reach their potential and lead more systematically to health-enhancing levels of physical activity, we must identify the factors that determine physical activity and lead to successful intervention engagement.

Typically, physical activity apps are built on self-monitoring of behavior, a behavior change technique (BCT) [12] established as a key ingredient of successful physical activity interventions [13]. However, the fact that self-monitoring is an effective BCT for an *average* participant does not mean that it will help all individuals or that everyone will use this technique. Tracking physical activity may not motivate all users and can even undermine motivation [14,15]. To increase the uptake and impact of physical activity apps, self-monitoring and tracking features may need to be combined with other behavioral or motivational techniques [16].

One of the factors influencing the uptake and commitment to self-monitoring and other self-regulatory BCTs is the quality of motivation for physical activity [17,18]. Individuals are more likely to set goals, make plans, and follow their progress when physical activity meets their psychological needs, corresponds to their life goals or identity, or brings them pleasure [17,18]. By addressing these determinants of autonomous motivation, interventions may help individuals more actively engage in self-regulatory BCTs.

A meta-analysis on physical activity motivation found that interventions that included any digital component produced significant cumulative effects on intention, stage of change, and autonomous motivation, but the meta-analysis included too few studies to specifically examine the motivational effects of smartphone-based interventions or identify which digital components most effectively increased motivation [19]. To optimize the motivational efficacy of smartphone-based interventions, it could be useful to digitally replicate versions of techniques drawn from face-to-face interventions, such as interpersonal interaction, sense of relatedness, empathy, and encouragement. These are central elements in satisfying the psychological needs of self-determination theory (SDT) and are systematically used in the interaction method known as motivational interviewing (MI). Supporting the needs of autonomy, competence, and relatedness and creating an empathetic environment could also increase the impact of smartphone-delivered interventions.

### Understanding How Individuals Change: N-of-1 Studies

Most evidence on the determinants of physical activity comes from group average–based *between participant* studies. Studying individual participants at the intraindividual level may help detect effects that differ from those found between participants [20,21]. Individuals can demonstrate associations of different strength and even in opposite directions when examining key variables of interest, a phenomenon that would be missed when observing group averages only. In addition, different predictors can be more influential for different individuals, as shown by Smith et al [22], who found that different social cognitive theory–based determinants predicted physical activity in the 6 adults they studied.

Studies conducted at the within-person (or idiographic) level include N-of-1 studies, which analyze each individual as their own study unit [23]. Such studies are increasingly being used in health psychology [22,24-27]. In addition to observing associations between variables, active manipulation of research conditions can be conducted with N-of-1 randomized controlled trials (RCTs), which may assess more than one intervention element during the same trial using a factorial design, as in the studies by Nyman et al [28] and Sniehotta et al [29]. These within-person RCTs use individuals as their own controls comparing periods with intervention elements with control periods with no active intervention elements [23]. In the past, N-of-1 RCTs in the field of health behavior change have required a research team member to actively deliver the interventions, prompting the participants to choose the right intervention with daily SMS text messages [29], delivering intervention allocation envelopes to the participants once a week in person [28], and collecting data from the participants every week in person [28,29]. This is time-consuming for both researchers and participants, leading to possible selection bias and increasing the risk of errors. Automating intervention delivery via a smartphone platform may improve measurement precision and data quality. N-of-1 RCTs are recommended by experts, especially for an individual’s treatment decisions [30] and for testing theoretical mechanisms within individuals [31]. However, within-person designs remain underused in behavior change research [32].

N-of-1 designs do not limit analyses to the individual level but also allow for aggregating data across all participants [29,33]. In aggregated multilevel models, individual-level variance is incorporated into the error terms, losing its informational value about individuals [34]. Conversely, aggregated multilevel models enable some level of generalization from the whole sample while adjusting for the individuals’ differences in the dependent variable [20]. Combining idiographic N-of-1 analyses with aggregated models can offer different perspectives on the same scientific question as all methodological approaches have their own biases [35].

Smartphones and wearable technology enable the continuous collection of individual-level data, suiting within-person studies [36]. Smartphone apps also enable N-of-1 RCTs that deliver prespecified intervention techniques to users at randomly allocated times.

### The Precious App

The Precious mobile app was designed building on theory and evidence to increase users’ physical activity using motivational and self-regulatory techniques [37]. The core functions of the app were as a tracking tool with self-regulatory BCTs, such as behavioral goal setting and self-monitoring, and motivational tools aimed at increasing uptake of the tracking features.

The motivational features of the Precious app draw on MI, a person-centered communication style that supports behavior change by increasing the salience of values and goals related to the desired behavior within an atmosphere of acceptance and compassion [38]. MI has shown promise in increasing physical activity with face-to-face or telephone-delivered interventions [39] and with computer-based interventions [40,41], but automated delivery of MI via smartphone apps has not been studied.

The Precious app combines MI with heart rate variability–based biofeedback to strengthen the mental link between an individual’s actions and well-being. Biofeedback has been found to reduce stress and anxiety about physical activity, removing a barrier for some inactive individuals [42]. A pilot study also found that biofeedback improved quality of life and reduced fatigue in a small sample of women with chronic fatigue syndrome [43]. Feasibility tests with the Precious app found promising participant engagement with the MI features [37]. In a 3-month usability RCT, the Precious app was found to be acceptable among persons with obesity, and participants were particularly satisfied with the app’s biofeedback report and physical activity modules [44].

### Theoretical Determinants in the Precious Trial

Several well-established theories suggest that individuals’ physical activity is shaped primarily by 2 key modifiable psychological factors: self-efficacy and motivation.

Self-efficacy is defined as one’s beliefs in their capability to successfully perform courses of action and achieve desired effects [45]. It is characterized as a key factor determining intentions to be physically active within health psychological theories including the health action process approach [46], social cognitive theory [47], the theory of planned behavior (as perceived behavioral control) [48], and SDT (as competence) [49]. High self-efficacy predicts physical activity across different study populations, especially when initiating activity [50,51].

Motivation is a key predictor of physical activity within, for example, SDT [49]. Motivation refers to the desire, urge, energy, or reason to perform a specific behavior [52]. One of the prerequisites for sustained motivation is the sense of competence, or self-efficacy, as the fulfillment of this psychological need is suggested to lead to an internalized desire to act [53]. This internalized or autonomous motivation is an established predictor of physical activity [54-56].

Despite the central position of self-efficacy and motivation in behavioral theory and interventions, few studies have observed whether changes in these predictors are followed by immediate changes in physical activity in everyday life [22,25,57]. Studies measuring behavioral variables typically compare very few time points and summarize the average effect of the predictors for the sample rather than providing effects for each participant [34,58]. Finding an association between self-efficacy, motivation, and physical activity *within* *individuals* repeatedly over time in everyday life would provide stronger evidence for predictive models [25].

### Objectives of This Study

This study is the first in a series of factorial N-of-1 experiments conducted using the Precious app, and it specifically sought to test the impacts of the digitalized MI (dMI) and biofeedback intervention components under study. The aims of this study were to (1) test whether the participants’ daily steps increased on intervention days when the app delivered motivational interventions and (2) examine the associations among self-efficacy, motivation, physical activity, and daily steps.

The analyses addressed the following research questions (RQs): (1) Do people take more steps on days when they are offered motivational smartphone-based interventions (intervention 1: MI components; intervention 2: biofeedback) compared with nonintervention days? (RQ 1) and (2) Do daily self-efficacy and motivation predict daily steps in individuals? (RQ 2).

## Methods

### Design

This study was a 40-day 2 × 2 factorial N-of-1 RCT testing the effects of dMI and biofeedback and involved twice-daily (morning and afternoon) ecological momentary assessments (EMAs) of psychological and environmental predictors of physical activity. The study has been reported following the Consolidated Standards of Reporting Trials Extension for reporting N-of-1 Trials (CENT) [59]. The trial was not formally preregistered as conventions for the registration of factorial N-of-1 experimental studies had not been established before this study’s commencement in 2016. However, a dated publicly available version of the study protocol was published just after the start of data collection and before any data analyses [60].

**Figure 1:**
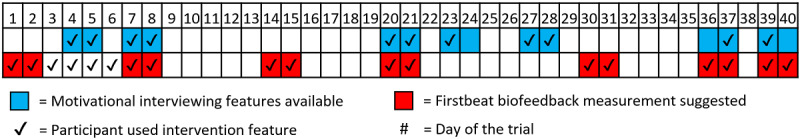
Example data from participant 1 showing the days in which the biofeedback and motivational interviewing interventions were offered and used. Participants had access to the following self-regulatory intervention components throughout the trial: behavioral goal setting, action planning, self-monitoring of behavior, and feedback on behavior.

The 40-day trial included 12 three-day active study periods (ie, 2 days during which one of the study conditions described in the Randomization section was implemented plus a wash-out day in which dMI features were hidden from the app and biofeedback notifications ceased), 2 additional control days (1 each after the fourth and eighth active study periods), and a 2-day lead-out period in which all app features were available (Figure 1). Wash-out days were included in the study design to eliminate any carryover effects of dMI or biofeedback on cognition or behavior on subsequent days [61].

### Randomization

Each active study period tested the effects of one of the following conditions: (1) both dMI and biofeedback, (2) dMI alone, (3) biofeedback alone, or (4) a control period in which neither intervention was delivered. The 4 conditions were repeatedly block randomized to one of the 12 three-day active study periods using a computer-generated code. This led to each of the 4 conditions being assigned to three 3-day study periods overall. Randomizing the timing of repeated interventions helps avoid potential time-based confounders that might systematically coincide with intervention delivery [62]. A code for activating the individually randomized trial was printed and sealed in an opaque envelope. Each participant drew one of the envelopes at the baseline meeting with a researcher (JN) and entered this code into the app to initiate the trial procedure.

### Blinding

As we aimed to test the impact of motivational features that require active cognitive engagement with the tasks [37], it was not possible to blind participants to the interventions they received on a given day. However, participants were not aware of the specific study hypotheses or analysis methods, and during the study, participants were blinded to the sequence in which intervention components would be delivered or available in the Precious app. Tests for blinding were not conducted.

### Interventions

**Figure 2:**
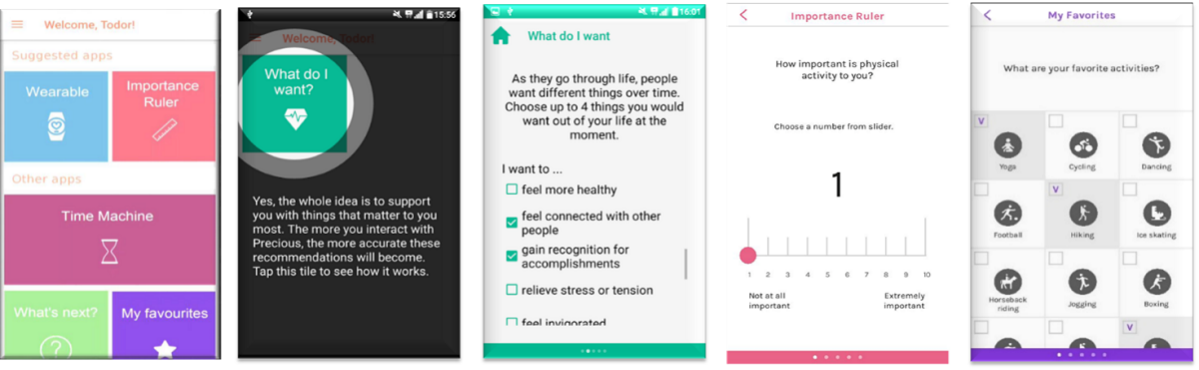
Example screenshots of the motivational interviewing features of the Precious app.

On MI intervention days, participants received a morning notification of new content: “A new test period has started. Come see what the Precious app has to offer.” The notification remained visible until it was touched to open the app or swiped away. The digitalized elements of MI (Figure 2) are described in detail in the study by Nurmi et al [37] and include the techniques listed in Table S1 in Multimedia Appendix 1. To imitate the interactivity of face-to-face MI, dMI tools were offered in a stepwise manner: 3 dMI tools in 3 consecutive periods. Set A included “What do I want” and “Choose Favorite PAs,” set B included “Importance Ruler” and “What’s Next (stage of change),” and set C included “Time Machine” and “Confidence Ruler.” Thus, the tools offered slight variability over the course of the trial to maintain user interest.

The biofeedback interventions started with a preparation notification the day before each measurement: “Tomorrow is a Firstbeat assessment day. Be sure to have the device charged and ready to wear tomorrow.” On the first intervention day, participants received a notification stating the following: “Today is the day! Please wear your Firstbeat device today and upload the data tomorrow.” On the second intervention morning, the notification read the following: “We hope you slept well. Please upload the data from your Firstbeat device to get a report on your activity, sleep, and stress levels.” To access the report, participants needed to plug the wearable sensor into their computer’s USB port and upload the data to the trial website. The biofeedback report was provided using the Firstbeat Bodyguard 2 heart rate variability monitor (Firstbeat Technologies Ltd). These data were first pushed securely to the Firstbeat company servers for analysis and then passed securely to the Precious server for storage. This intervention used BCTs 2.4 (self-monitoring outcomes), 2.6 (biofeedback), 2.7 (feedback on outcomes of behavior), 7.1 (prompts or cues), and 12.5 (adding objects to the environment) [12].

### Always Available App Features

On control and wash-out days, the dMI and biofeedback tools were hidden from the app. The participants could still freely access all the self-regulatory BCTs, including behavioral goal setting and self-monitoring (the full list is available in Table S1 in Multimedia Appendix 1). They also received communication related to their goal progress at 5 PM every day: “You’ve taken [step total] steps today—that’s (100*[step total]/[goal amount])% of your goal. Keep going!” (BCT 2.2, feedback on behavior and goal progress [12]). If participants set a step goal and reached it, they received a congratulatory message: “Good job! You’ve achieved your step goal for today. Click here to see your progress.” (BCT 10.4, social reward [12]).

### Outcomes

#### Physical Activity Data Collection

The primary outcome was daily steps, assessed continuously throughout the study using a waterproof Xiaomi Mi Band wrist-worn accelerometer. Participants agreed to wear the Mi Band for the entire duration of the 40-day trial and to only remove it temporarily. Mi Band had a step count accuracy of 96.6%, which placed it among the most accurate commercially available, Bluetooth-enabled wrist-worn step counters at the outset of this trial [63]. Time-stamped step counts were passed from the Mi Band to the Precious app via Bluetooth every 10 minutes.

#### Daily EMA

Participants were invited to report their *self-efficacy* every morning between 9 AM and 10 AM with the following question—“At this moment, how confident are you that you can be physically active for at least 30 min today?”—on a 9-step visual analog scale from “not at all” to “extremely.”

Participants were invited to report their *motivation* twice each day (once between 9 AM and 10 AM and once between 4 PM and 5 PM) with the following question—“At this moment, how motivated are you to be physically active”—on a 9-step visual analog scale from “not at all” to “extremely.”

Participants were invited to report their *perceived barriers to being active on that day* each day between 9 AM and 10 AM to control for external factors that were not captured by our predictors with the following question—“To what extent are other things you need to do today stopping you from being physically active?”—on a 9-step scale from “not at all” to “extremely.”

Participants were invited to report their *pain or illness* every day between 4 PM and 5 PM with the following question—“Did illness or pain stop you from being physically active today?”—and 3 options: “none,” “somewhat,” and “seriously.”

The EMA questions were based on previous research [64]. To minimize measurement reactivity and separate these daily assessments from the motivational interventions of the Precious app, the questions were delivered to participants’ phones via a specific EMA measurement app developed by the Netherlands Organisation for Applied Scientific Research.

Intervention use was conceptualized as (1) participants accessing a specific MI feature on the day the feature was available and (2) participants uploading the biofeedback report to the server. Both actions left a time stamp on the server file.

### Participants

Participants were recruited from the general population using advertisements in the *Metro* newspaper of the Helsinki area, Finland, and a Facebook page and targeted advertisements in October 2016. People who responded to the advertisement were contacted by the research team via email or phone to establish whether they met the following eligibility criteria: age of >18 years, ability to speak Finnish, ability to read and understand English, no contraindications to engaging in physical activity as assessed using the Physical Activity Readiness Questionnaire [65], ownership and use of a smartphone with a compatible operating system (Jelly Bean version 4.1 or higher for Android), willingness to install the Precious app on said smartphone for a 40-day period, no use of any physical activity trackers (eg, Fitbit, Garmin, or Misfit) or physical activity apps in the previous 6 months, no participation in other trials or behavior change programs in the previous 6 months or during the trial, levels of physical activity below the recommendation of 150 minutes per week of moderate-intensity physical activity [66], and willingness to wear an activity tracker for the duration of the study. In addition, participants were excluded if they were seeking to be enrolled in the trial concurrently with a friend or relative. This exclusion criterion was applied to avoid exposure to randomly timed intervention materials during control days through the other person’s phone.

Of the 147 people who responded to the advertisement, 48 (32.7%) did not respond to efforts to contact them, 24 (16.3%) were too active, 24 (16.3%) already used a physical activity tracker or health app, 16 (10.9%) did not have the necessary technical set-up, 6 (4.1%) were excluded as they wished to participate with a friend or spouse, 5 (3.4%) were undergoing other interventions, 4 (2.7%) had poor health, and 2 (1.4%) had scheduling conflicts. The remaining 17 people met all the inclusion criteria, but 2 (12%) declined to participate as they did not wish to do so without a friend or spouse who did not meet the inclusion criteria. This resulted in a final sample of 15 participants.

### Sample Size

The number of participants was limited by available resources (eg, activity bracelets, heart rate variability sensors, and technical support). The intervention length was limited partly by the estimated battery life of Mi Band activity bracelets (approximately 40-50 days without charging) and partly to avoid major public holidays that might affect participants’ physical activity. The planned sample of 15 participants with 40 observation days would yield 600 observation days in total, which was similar to that of earlier factorial N-of-1 RCTs that included 8 participants for 62 days and 10 participants for 60 days [28,29].

### Procedure

Participants were invited for a face-to-face intake session from October 2016 to November 2016 in which they were asked to read the study information sheet (which had also been sent to them by email) and could ask questions. Individuals wishing to be enrolled in the study then provided informed consent to participate and began the study.

To begin the study, participants had a 60- to 90-minute long individual instruction session with a researcher (JN) at a university office. After receiving information on the study and signing informed consent sheets in person, participants randomly chose an envelope from a bag. Each opaque envelope contained a study code, which was entered into the Precious app to carry out the study period randomization procedure described previously.

Participants received help to install the Precious and EMA apps on their phones and entered their study code into the Precious app. The researcher then instructed participants on how to use these apps. Participants then received the Mi Band activity bracelet and learned how to pair it with the Precious app. For biofeedback measurements, they then received a Firstbeat Bodyguard 2 device and were taught how to conduct heart rate variability measurements and read these reports at home on their PCs. Participants were advised to follow the instructions of the apps until their individual follow-up meetings. Printed instructions and researchers’ contact details were provided with the material pack, and participants were encouraged to contact the researchers in case of any technical difficulties. All participants received a portable power bank as a gift for participating and to help keep their phones charged during the trial.

After the 40-day trial, participants returned to the university for an individual follow-up meeting, debriefing, and exit interview and to return the activity bracelet and heart rate variability monitor. A researcher (JN or KK) downloaded the wristband data that were stored locally on participants’ phones and helped participants uninstall the study apps. Participants were then asked about their experiences using the Precious app over the course of the study during a semistructured interview. At the end, participants were rewarded with 3 movie tickets and thanked for their participation.

### Statistical Methods

#### Preliminary Analyses

The analyses were conducted using SPSS Statistics (version 24; IBM Corp). Steps and daily EMA scores were plotted to visualize temporal patterns, check for nonlinear patterns, and visually explore and compare individuals’ scores as recommended in the studies by Bolger and Laurenceau [20], McDonald et al [32], and Manolov et al [67].

#### N-of-1 Analyses

Participants’ time-series data sets were analyzed separately using dynamic regression [32,68] as this method can accommodate small sample sizes [69]. Dynamic regression modeling aims to capture the impact of past observations on an outcome by building autoregressive lag models and, therefore, can be used to account for regular cyclical patterns that may occur within an individual’s physical activity levels over time [70]. To maximize statistical power, wash-out days were included in these analyses and treated as control days (as no interventions were delivered).

To investigate the effects of MI and biofeedback interventions on daily steps, a multistep procedure was followed. First, we examined linear and quadratic trends within the time series using curve estimation and retained any statistically significant time trends as predictors in subsequent models. Next, we examined cyclical patterns within the data by examining the day of the week as a predictor of daily steps. For participants for whom autocorrelation in steps was likely based on autocorrelation plots and the statistical threshold of *P*<.05, lagged step variables were created and used as controls.

Any variables that predicted steps in these initial models were retained for the final model, which in addition introduced 2 dichotomous intervention delivery variables (ie, delivery of dMI and delivery of biofeedback) as primary predictors of daily steps and perceived barriers and pain as control variables. This procedure was then repeated to examine the relationship between morning motivation and morning self-efficacy and participants’ daily step totals.

#### Aggregated Analyses

To investigate the effects of the interventions on participants’ steps, we used random intercept multilevel modeling on an aggregated data set that *excluded* wash-out days (465 possible observations; 409 after accounting for missing step data). First, a null model (model 1) was run to fit the grand mean for steps, provide a baseline for the model fit statistics, and assess the intraclass correlation coefficient (ICC). Time was then added as a fixed factor in model 2 and also as a random factor in model 3. In model 4, we added the day of the week (to control for weekly repeating patterns in steps) and within- and between-subject levels of pain or illness and perceived barriers (to control for factors that were not under the participants’ control, which varied both within and between persons [20]). Model 5 introduced 2 dichotomous intervention delivery variables (ie, delivery of dMI and delivery of biofeedback) as the primary predictors of daily steps. Finally, in model 6, we examined whether including autocorrelation patterns improved model fit.

To examine the associations between morning motivation and morning self-efficacy and participants’ steps, we used random intercept multilevel modeling on an aggregated data set that *included* wash-out days (600 possible observations; 530 after accounting for missing step data). We included wash-out days in these analyses as we did not expect carryover effects of the interventions to moderate the examined relationships between independent variables (ie, motivation and self-efficacy) and step counts. In these analyses, we followed the same 6-step modeling process described previously, but model 5 introduced within- and between-person measures of motivation or self-efficacy instead of intervention delivery variables

To facilitate interpretation of the intercept in all multilevel analyses, the scores were grand mean and person mean centered. Iterative improvements in model fit were assessed by conducting a chi-squared test using the difference in deviance (−2 log likelihood) between successive models as the chi-squared test value and the difference in *df* between successive models as the *df* for the chi-squared test.

### Ethics Approval

The University of Helsinki Ethical Review Board in the Humanities and Social and Behavioural Sciences granted a favorable decision for this study (statement 3/2016).

## Results

### Overview

A total of 15 healthy adults (n=4, 27% male and n=11, 73% female) took part in the study, with ages ranging from 28 to 57 (mean 42.33, SD 9.82) years. All participants reported wearing the activity bracelet continuously for the duration of the trial; however, some participants were missing step data because of technical errors, including participant 9, who had no step data, and participant 2, who had step data for only 53% (21/40) of the measurement days. This left a total of 88.3% (530/600) of usable data points. Missing values were not imputed as the missing completely at random test [71] found the study values not to be missing completely at random (*χ*^2^_25_=85.3; *P*<.001) and common multiple-imputation methods cannot reliably handle this potential bias [72,73].

All participants (15/15, 100%) finished the trial, returned for the follow-up meeting, reported wearing the activity bracelet continuously for the entire duration of the trial, and kept using features of the Precious app throughout the trial. Participants engaged with dMI features during an average of 5.10 (SD 1.0; range 3-7) out of the 7 intervention periods and conducted biofeedback measurements during an average of 5.67 (SD 1.4; range 2-7) out of 7 intervention periods. All participants (15/15, 100%) conducted biofeedback measurements, but 4 of them partly missed the suggested intervention days: 3 (75%) conducted a measurement a day later than suggested, and 1 (25%) conducted all their biofeedback measurements during control days. Unforeseen technical problems prevented 13% (2/15) of the participants from accessing their biofeedback reports during the trial (details in Table 1 and Table S1 in Multimedia Appendix 2). Among the 13 participants for whom data were available, step count goals were set on 42.7% (222/520) of the trial days, with a wide range of 0 to 38 days out of 40. These data are presented visually in Figure S1 in Multimedia Appendix 3.

**Table 1:** Participant characteristics, intervention use, completion of ecological momentary assessment (EMA) measurements, and daily steps by participant (n=40 days).

Participant	Age (years)^a^	Sex	Motivational interviewing interventions used (n=7^b^), n (%)	Biofeedback interventions used (n=7^c^), n (%)	Morning EMA measurements completed, n (%)				Evening EMA measurements completed, n (%)			Daily steps, mean (SD)
					Motivation	Self-efficacy	Perceived barriers	Motivation		Pain or illness		
P01	60	Male	7 (100)	7 (100)^d^	28 (70)	28 (70)	28 (70)	29 (73)		29 (73)	7463 (3660)	
P02	40	Female	6 (86)	6 (86)	37 (93)	37 (93)	37 (93)	37 (93)		37 (93)	18,218 (3542)	
P03	40	Male	5 (71)	5 (71)^e^	34 (85)	34 (85)	34 (85)	38 (95)		38 (95)	12,216 (2642)	
P04	50	Female	5 (71)	2 (29)^f^	20 (50)	20 (50)	20 (50)	21 (53)		19 (48)	8044 (2451)	
P05	30	Female	3 (43)	4 (57)	39 (98)	39 (98)	39 (98)	39 (98)		39 (98)	20,640 (4378)	
P06	40	Female	4 (57)	6 (86)	37 (93)	37 (93)	37 (93)	35 (88)		35 (88)	7812 (2465)	
P07	30	Male	5 (71)	6 (86)	29 (73)	29 (73)	29 (73)	28 (70)		28 (70)	6447 (3313)	
P08	40	Female	5 (71)	6 (86)^e^	39 (98)	39 (98)	39 (98)	39 (98)		39 (98)	8868 (1873)	
P09	50	Female	4 (57)	5 (71)	34 (85)	34 (85)	34 (85)	32 (80)		32 (80)	—^g^	
P10	60	Male	5 (71)	7 (100)	37 (93)	37 (93)	37 (93)	36 (90)		36 (90)	16,167 (2593)	
P11	30	Female	6 (86)	7 (100)	39 (98)	39 (98)	39 (98)	38 (95)		38 (90)	12,134 (5573)	
P12	30	Female	6 (86)	—	37 (93)	37 (93)	37 (93)	38 (95)		37 (93)	8840 (3626)	
P13	60	Female	—	—	38 (95)	38 (95)	38 (95)	39 (98)		39 (98)	8809 (4482)	
P14	40	Female	—	—	36 (90)	36 (90)	36 (90)	37 (93)		37 (93)	7606 (3121)	
P15	40	Female	5 (71)	7 (100)	39 (98)	39 (98)	39 (98)	39 (98)		39 (98)	13,415 (3287)	

^a^Age rounded to the nearest multiple of 10 for anonymization.

^b^Motivational interviewing interventions were available 7 times.

^c^Biofeedback interventions were suggested 7 times via smartphone notifications.

^d^The participant conducted an additional biofeedback measurement during the first week.

^e^The participant conducted 1 biofeedback measurement a day late and received the report during the wash-out day.

^f^The participant conducted both measurements during control days.

^g^Not available; these use data are missing because of a server log problem.

### Time Trends and Periodicity

Visual analysis of sequence charts (Multimedia Appendix 4) confirmed that individuals’ steps varied sufficiently over time to conduct analyses [32]. Individual participants’ steps did not show statistically significant time trends except for participant 4, whose steps slightly decreased over time (*B*=−84, SD 39, 95% CI −163 to −5; *R*^2^=0.12; *P*=.04). However, visual assessment showed a decline in participant 4’s steps only during control days (Multimedia Appendix 4). Multimedia Appendix 4 also shows how participants’ average activity levels and progress over time differed between individuals. Weekday-related patterns in physical activity were detected and controlled for in participants 10, 11, and 13; autoregression was detected in participants 7, 10, 11, 14, and 15; and their individual lag was adjusted with a pertinent step lag variable in the dynamic regressions.

### RQ 1: Intervention Effects on Daily Steps

The overall mean daily steps were 10,786 (SD 5393) on dMI intervention days, 11,125 (SD 5360) on biofeedback intervention days, and 11,053 (SD 5922) on control days (including wash-out days). See Table 2 for additional step count data. The dMI and biofeedback interventions did not show any statistically significant associations with daily steps for any individual participant (all *P*>.05; Table 3).

**Table 2:** Mean and SD of steps on the days specific intervention elements were available and on control days.

Participant	Firstbeat Biofeedback, mean (SD)		Motivational interviewing, mean (SD)		Days when both interventions were available, mean (SD)	Control days (including wash-out days), mean (SD)
	Days when Firstbeat Biofeedback was suggested	Days when Firstbeat Biofeedback was not suggested	Days when motivational interviewing was available	Days when motivational interviewing was unavailable		
All	11,125 (5360)	10,953 (5448)	10,786 (5393)	11,127 (5427)	10,937 (5514)	11,053 (5922)
P01	7348 (2572)	7517 (4111)	6670 (2384)	7830 (4107)	6982 (3079)	7865 (4578)
P02	18,813 (4142)	17,980 (3404)	18,115 (5667)	18,241 (3108)	20,513 (8370)	18,327 (3501)
P03	12,495 (3825)	12,065 (1792)	11,780 (3434)	12,450 (2143)	12,324 (4460)	12,368 (1833)
P04	8643 (1821)	7731 (2706)	8550 (1969)	7841 (2627)	8943 (2187)	7683 (2908)
P05	20,472 (5144)	20,730 (4014)	20,508 (4810)	20,710 (4225)	21,398 (4707)	21,152 (3675)
P06	7181 (2202)	8152 (2572)	7413 (2172)	8028 (2625)	6836 (2234)	8144 (2765)
P07	7670 (2957)	5733 (3357)	7143 (3288)	6040 (3328)	7602 (3278)	5467 (3366)
P08	8566 (1356)	9031 (2107)	8586 (2009)	9020 (1818)	8491 (1508)	9126 (1970)
P10	16,478 (2454)	15,999 (2697)	16,108 (1891)	16,198 (2937)	15,879 (2316)	15,874 (3013)
P11	12,701 (5623)	11,828 (5633)	13,716 (4988)	11,282 (5775)	13,005 (5779)	10,978 (5850)
P12	10,146 (4050)	8187 (3284)	8004 (3450)	9258 (3705)	9403 (2952)	8731 (3103)
P13	7460 (3772)	9535 (4730)	8543 (4555)	8952 (4527)	6763 (3819)	9120 (4791)
P14	7753 (2831)	7533 (3308)	6339 (3466)	8240 (2790)	7754 (2635)	8387 (2697)
P15	13,214 (3182)	13,523 (3399)	13,020 (2574)	13,628 (3643)	12,327 (2565)	13,397 (3673)

**Table 3:** The effect of digitalized motivational interviewing (dMI) and Firstbeat Biofeedback on individual participants’ daily steps.

Participant			B (SE; 95% CI)		*P* value
**P01^a^**					
	dMI	−1122 (1626; −4486 to 2242)		.50	
	Biofeedback	413 (1632; −2962 to 3788)		.80	
	Perceived barriers	−484 (471; −1459 to 490)		.32	
**P02**					
	dMI	−1033 (1744; −4749 to 2683)		.56	
	Biofeedback	1418 (1881; −2591 to 5426)		.46	
	Perceived barriers	−646 (410; −1519 to 227)		.14	
	Pain or illness	−1661 (1264; −4355 to 1033)		.21	
**P03^a^**					
	dMI	−492 (1037; −2609 to 1625)		.64	
	Biofeedback	516 (1012; −1550 to 2582)		.61	
	Perceived barriers	−750 (461; −1692 to 192)		.11	
**P04**					
	dMI	−333 (3492; −15,356 to 14,690)		.93	
	Biofeedback	1968 (3726; −14,063 to 18,000)		.65	
	Perceived barriers	−431 (679; −3352 to 2490)		.59	
	Pain or illness	−411 (4816; −21,131 to 20,308)		.94	
	Time	−54 (128; −604 to 495)		.71	
**P05^a^**					
	dMI	354 (1734; −3167 to 3875)		.84	
	Biofeedback	−775 (1774; −4377 to 2826)		.67	
	Perceived barriers	559 (730; −923 to 2042)		.45	
**P06**					
	dMI	−194 (1040; −2325 to 1936)		.85	
	Biofeedback	−1044 (1083; −3263 to 1176)		.34	
	Perceived barriers	−47 (168; −390 to 296)		.78	
	Pain or illness	511 (1283; −2118 to 3139)		.69	
**P07^b^**					
	dMI	346 (1398; −2633 to 3324)		.81	
	Biofeedback	628 (1413; −2384 to 3640)		.66	
	Perceived barriers	−142 (309; −800 to 516)		.65	
	Pain or illness	−1995 (766; −3627 to −363)		.02	
**P08**					
	dMI	−391 (723; −1862 to 1081)		.59	
	Biofeedback	−372 (734; −1866 to 1122)		.62	
	Perceived barriers	−123 (142; −412 to 165)		.39	
	Pain or illness	35 (2102; −4242 to 4312)		.99	
**P10^a,c^**					
	dMI	−797 (943; −2725 to 1132)		.41	
	Biofeedback	350 (944; −1581 to 2281)		.71	
	Perceived barriers	167 (329; −504 to 839)		.61	
	Day of the week	512 (218; 66 to 957)		.03	
**P11^d^**					
	dMI	1196 (1914; −2747 to 5138)		.54	
	Biofeedback	4 (2135; −4393 to 4401)		>99	
	Perceived barriers	34 (419; −828 to 896)		.94	
	Pain or illness	−2857 (2858; −8744 to 3030)		.33	
	Day of the week	−581 (468; −1545 to 383)		.23	
**P12**					
	dMI	−2190 (1381; −5010 to 630)		.12	
	Biofeedback	2462 (1351; −298 to 5222)		.08	
	Perceived barriers	108 (219; −339 to 554)		.63	
	Pain or illness	−1239 (1519; −4341 to 1864)		.42	
**P13**					
	dMI	−437 (1205; −2892 to 2019)		.72	
	Biofeedback	−773 (1300; −3422 to 1876)		.56	
	Perceived barriers	−667 (245; −1166 to −169)		.01	
	Pain or illness	−3337 (1047; −5471 to −1204)		.003	
	Day of the week	−722 (300; −1333 to −111)		.02	
**P14^e^**					
	dMI	−1594 (877; −3391 to 203)		.08	
	Biofeedback	1624 (878; −175 to 3424)		.08	
	Perceived barriers	852 (269; 302 to 1403)		.004	
	Pain or illness	−2654 (836; −4367 to −942)		.004	
**P15^a,f^**					
	dMI	−1315 (1004; −3377 to 748)		.20	
	Biofeedback	129 (973; −1870 to 2128)		.90	
	Perceived barriers	−602 (242; −1100 to −105)		.02	
	Day of the week	−337 (248; −846 to 172)		.19	

^a^The participant did not report any pain or illness.

^b^A 2-day step lag (*P*=.02) was included in the analysis of P07.

^c^A 4-day step lag (*P*=.03) was included in the analysis of P10.

^d^A 7-day step lag (*P*=.03) was included in the analysis of P11.

^e^A 1-day step lag (*P*=.01) was included in the analysis of P14.

^f^6-day (*P*=.006) and 7-day (*P*=.04) step lags were included in the analysis of P15.

### RQ 1: Aggregated Effects of the Intervention Components on Daily Steps

The average number of steps of participants across all time points (excluding wash-out days; fixed effect in the null model) was 11,137, and participants’ overall steps did not significantly change over the course of the study. The data showed no advantage of a quadratic fit compared with a linear fit. The differences between participants’ step slopes explained 21% of the variance in steps over time. The ICC was high, with 57.1% of the variance attributable to differences between participants. The covariance between slope and intercept was nonsignificant (*P*=.53), suggesting that the initial level of steps did not affect changes over time.

Multilevel models of the intervention effects did not identify any significant associations between condition and steps (dMI: *B*=−246, 95% CI −1012 to 520, SE 389, t_312_=0.63, and *P*=.53; biofeedback: *B*=67, 95% CI −688 to 821, SE 384, t_311_=−0.17, and *P*=.86). Adjusting for autoregression did not significantly improve the model fit, and there was no association between participants’ steps at adjacent time points (*B*=0.12, SE 0.07; *P*=.11). Table 4 presents the series of models used, and Table 5 shows the details of the model that best fit the data. The day of the week variable revealed that participants were most active on Mondays and Wednesdays, taking 2303 and 1609 steps more, respectively, than on Sundays. Multimedia Appendix 4 shows that intercepts and slopes varied substantially between participants on both intervention and nonintervention days.

**Table 4:** Sequential multilevel models used to investigate the effects of the interventions on steps.

Model	Modeled parameters (*df*)	–2LL^a^ model deviance	Chi-squared (*df*) for change in model fit^b^	*P* value	BIC^c^
1	Fixed and random intercepts (1)	7882.7	N/A^d^	N/A	7900.8
2	Model 1+fixed time (2)	7879.5	3.2 (1)	.07	7903.6
3	Model 2+random time (3)	7877.4	2.1 (1)	.15	7913.5
4	Model 3+day of the week+barriers to PA^e^ (within and between persons)+pain or illness (within and between persons)	6363.3	1514 (10)	<.001	6456.2
5	Model 4+dMI^f^ on or off+biofeedback on or off	6362.9	0.4 (2)	.82	6467.4
6	Model 5+autocorrelation (within persons)	6360.5	2.4 (1)	.12	6470.7

^a^2LL: −2 log likelihood.

^b^Chi-squared test statistic and *df* derived from differences between model N and model N – 1 in deviance and *df*, respectively.

^c^BIC: Bayesian information criterion.

^d^N/A: not applicable.

^e^PA: physical activity.

^f^dMI: digitalized motivational interviewing.

**Table 5:** Parameter estimates for multilevel model 6 investigating daily steps as a function of the availability of the motivational interviewing and biofeedback interventions excluding wash-out days.

Type of effect	Variables in model	B (SE; 95% CI)	*t* test (*df*)	*P* value
Fixed	Intercept	10,513 (1161; 8109 to 12,918)	9.04 (23)	<.001
Fixed	Time	−39 (21; −83 to 6)	−1.86 (15)	.08
Fixed	dMI^a^	−246 (389; −1012 to 520)	0.63 (312)	.53
Fixed	Biofeedback	67 (384; −688 to 821)	−0.17 (311)	.86
Fixed	Within-person perceived barriers	−111 (92; −292 to 69)	−1.21 (312)	.23
Fixed	Between-person perceived barriers	820 (758; −805 to 2445)	1.08 (14)	.30
Fixed	Within-person pain or illness	−2228 (490; −3191 to –1265)	−4.55 (314)	<.001
Fixed	Between-person pain or illness	−4039 (4199; −13,018 to 4939)	−0.96 (14)	.35
Random (between persons)	Intercept UN (1,1): intercept variance	12,253,862 (5,555,328; 5,039,391 to 29,796,684)	2.21	.03
Random (between persons)	UN (2,2): slope variance	1158 (2060; 35 to 37,828)	0.56	.57
Random (between persons)	UN (2,1): covariance	50,792 (81,582 (−109,105 to 210,689)	0.62	.53
Random (within persons)	Residual AR1^b^ diagonal	11,318,313 (937,548; 9,622,151 to 9,622,151)	12.07	<.001
Random (within persons)	Autocorrelation AR1 ρ	0.12 (0.07; −0.03 to 0.26)	1.59	.11

^a^dMI: digitalized motivational interviewing.

^b^AR1: autoregression of lag 1.

### RQ 2: Associations Between Daily Self-Efficacy and Motivation and Daily Steps

Morning self-efficacy predicted a higher number of steps during the day in 27% (4/15) of the participants (Table 6), and morning motivation predicted a higher number of steps during the day in 20% (3/15) of the participants (Table 7). Self-efficacy and motivation were analyzed and are presented separately because of their theory-based association [46,48,49] and high correlation in the sample (*r*=0.597).

**Table 6:** Dynamic regression of morning self-efficacy and daily steps controlling for perceived barriers and pain or illness^a^.

Participant			B (SE; 95% CI)		*P* value
**P01^b^**					
	Self-efficacy	1007 (357; 270 to 1744)		.009	
	Perceived barriers	−574 (401; −1401 to 254)		.17	
**P02**					
	Self-efficacy	206 (410; −664 to 1075)		.62	
	Perceived barriers	−447 (400; −1294 to 400)		.28	
	Pain or illness	−1799 (1205; −4353 to 755)		.16	
**P03^b^**					
	Self-efficacy	−101 (557; −1237 to 1035)		.86	
	Perceived barriers	−864 (518; −1922 to 193)		.11	
**P04**					
	Self-efficacy	−337 (511; −1963 to 1289)		.56	
	Perceived barriers	−370 (494; −1942 to 1203)		.51	
	Pain or illness	1105 (3991; −11,596 to 13,806)		.80	
	Time	−93 (113; −454 to 268)		.47	
**P05^b^**					
	Self-efficacy	931 (437; 42 to 1820)		.04	
	Perceived barriers	30 (310; −600 to 660)		.92	
**P06**					
	Self-efficacy	63 (258; −463 to 590)		.81	
	Perceived barriers	51 (253; −466 to 567)		.84	
	Pain or illness	260 (1245; −2287 to 2807)		.84	
**P07^c^**					
	Self-efficacy	8 (394; −828 to 844)		.99	
	Perceived barriers	−124 (311; −784 to 535)		.70	
	Pain or illness	−2048 (1181; −4553 to 456)		.10	
**P08**					
	Self-efficacy	66 (305; −554 to 685)		.83	
	Perceived barriers	−84 (173; −436 to 268)		.63	
	Pain or illness	−176 (2019; −4278 to 3927)		.93	
**P10^b,d^**					
	Self-efficacy	578 (442; −325 to 1480)		.20	
	Perceived barriers	182 (314; −459 to 822)		.57	
	Day of the week	412 (219; −35 to 859)		.07	
**P11^e^**					
	Self-efficacy	1161 (325; 493 to 1829)		.001	
	Perceived barriers	583 (344; −124 to 1289)		.10	
	Pain or illness	−3685 (2055; −7908 to 539)		.09	
	Day of the week	−145 (398; −963 to 672)		.72	
**P12**					
	Self-efficacy	−160 (212; −592 to 272)		.46	
	Perceived barriers	133 (225; −326 to 592)		.56	
	Pain or illness	−1258 (1515; −4348 to 1832)		.41	
**P13**					
	Self-efficacy	438 (269; −110 to 986)		.11	
	Perceived barriers	−497 (259; −1025 to 30)		.06	
	Pain or illness	−2345 (1076; −4533 to −156)		.04	
	Day of the week	−727 (276; −1287 to −166)		.01	
**P14^f^**					
	Self-efficacy	435 (136; 157 to 714)		.003	
	Perceived barriers	580 (261; 46 to 1114)		.03	
	Pain or illness	−1599 (819; −3275 to 76)		.06	
**P15^b,g^**					
	Self-efficacy	37 (461; −912 to 985)		.94	
	Perceived barriers	−695 (352; −1418 to 28)		.06	
	Day of the week	−420 (239; −911 to 71)		.09	

^a^Motivation and perceived barriers were measured at 9 AM. Pain or illness was measured at 4 PM. Time, day of the week, and lagged steps were added to participants whose data showed statistically significant time, periodical, or autoregressive effects, as in the study by McDonald et al [32].

^b^The participant did not report any pain or illness.

^c^A 2-day step lag (*P*=.01) was included in the analysis of P07.

^d^A 4-day step lag (*P*=.08) was included in the analysis of P10.

^e^A 7-day step lag (*P*=.005) was included in the analysis of P11.

^f^A 1-day step lag (*P*=.07) was included in the analysis of P14.

^g^A 6-day (*P*=.004) and 7-day (*P*=.10) step lags were included in the analysis of P15.

**Table 7:** Dynamic regression of morning motivation and daily steps controlling for perceived barriers and pain or illness for all participants^a^.

Participant			B (SE; 95% CI)		*P* value
**P01^b^**					
	Motivation	1321 (348; 602 to 2040)		<.001	
	Perceived barriers	−325 (368; −1084 to 434)		.39	
**P02**					
	Motivation	177 (418; −709 to 1062)		.68	
	Perceived barriers	−528 (373; −1319 to 263)		.18	
	Pain or illness	−1721 (1263; −4398 to 955)		.19	
**P03^b^**					
	Motivation	1328 (809; −322 to 2978)		.11	
	Perceived barriers	−726 (426; −1595 to 143)		.10	
**P04**					
	Motivation	−270 (857; −2996 to 2456)		.77	
	Perceived barriers	−400 (643; −2446 to 1647)		.58	
	Pain or illness	−188 (4160; −13,428 to 13,052)		.97	
	Time	−63 (109; −409 to 284)		.61	
**P05^b^**					
	Motivation	1119 (486; 134 to 2105)		.03	
	Perceived barriers	847 (626; −422 to 2116)		.18	
**P06**					
	Motivation	226 (263; −311 to 763)		.40	
	Perceived barriers	176 (256; −348 to 700)		.50	
	Pain or illness	40 (1253; −2523 to 2602)		.98	
**P07^c^**					
	Motivation	−240 (697; −1717 to 1237)		.74	
	Perceived barriers	−169 (321; −850 to 512)		.61	
	Pain or illness	−2309 (1018; −4466 to −151)		.04	
**P08**					
	Motivation	129 (224; −327 to 584)		.57	
	Perceived barriers	−79 (147; −377 to 220)		.60	
	Pain or illness	−167 (1990; −4213 to 3878)		.93	
**P10^b,d^**					
	Motivation	786 (736; −717 to 2290)		.29	
	Perceived barriers	304 (361; −433 to 1042)		.41	
	Day of the week	488 (211; 57 to 920)		.03	
**P11^e^**					
	Motivation	1497 (320; 838 to 2155)		<.001	
	Perceived barriers	541 (297; −69 to 1151)		.08	
	Pain or illness	−2511 (1842; −6296 to 1275)		.18	
	Day of the week	−250 (348; −965 to 464)		.48	
**P12**					
	Motivation	−218 (199; −624 to 188)		.28	
	Perceived barriers	139 (221; −311 to 589)		.53	
	Pain or illness	−978 (1530; −4098 to 2142)		.53	
**P13**					
	Motivation	353 (303; −263 to 969)		.25	
	Perceived barriers	−588 (251; −1098 to −78)		.03	
	Pain or illness	−2610 (1074; −4796 to −424)		.02	
	Day of the week	−714 (286; −1295 to −133)		.02	
**P14^f^**					
	Motivation	94 (219; −354 to 542)		.67	
	Perceived barriers	836 (289; 245 to 1426)		.007	
	Pain or illness	−2523 (917; −4400 to −647)		.01	
**P15^b,g^**					
	Motivation	−213 (386; −1005 to 589)		.59	
	Perceived barriers	−641 (292; −1240 to −43)		.04	
	Day of the week	−264 (253; −782 to 255)		.31	

^a^Motivation and perceived barriers were measured at 9 AM. Pain or illness was measured at 4 PM. Time, day of the week, and lagged steps were added to participants whose data showed statistically significant time, periodical, or autoregressive effects, as in the study by McDonald et al [32].

^b^The participant did not report any pain or illness.

^c^A 2-day step lag (*P*=.007) was included in the analysis of P07.

^d^A 4-day step lag (*P*=.03) was included in the analysis of P10.

^e^A 7-day step lag (*P*=.01) was included in the analysis of P11.

^f^A 1-day step lag (*P*=.05) was included in the analysis of P14.

^g^A 6-day (*P*=.006) and 7-day (*P*=.05) step lags were included in the analysis of P15.

### RQ 2: Aggregated Associations Between Self-Efficacy and Motivation and Daily Steps

Table 8 presents the series of models used to investigate associations between motivation and self-efficacy and daily steps, and Table 9 shows the details of the models that best fit the data. When including wash-out days, the ICC was high—approximately 61.4% of the variance in steps was attributable to differences between participants. The average number of steps across all participants and time points (fixed effect in the null model) was 11,185. The average starting level of steps across participants was 11,789. The fixed effect of time on steps was statistically significant and negative (*B*=−0.32, 95% CI −58 to 5; *P*=.02); however, adding time to the model did not improve the model fit, and the linear change over time explained only 1.1% of the variability within participants in their steps. The data showed no advantage of a quadratic fit compared with a linear fit. Adding a random effect of time did not improve the model fit. However, the variation in the growth model between participants seemed significant—adding a random effect of time when also letting the intercept and slope correlate (UN) explained 13% of the between-person intercept variance (difference between individuals). This would indicate that participants had different starting step levels and trajectories over time, as shown in Multimedia Appendix 4. The covariance between slope and intercept—UN (2,1)—was nonsignificant (*P*=.23), suggesting that the initial level of steps did not affect how much they changed over time. Adding the control variables day of the week, within- and between-person perceived barriers, and pain or illness clearly improved the model fit (*P*<.001) and explained 5.4% of the within-person variance and 3.3% of the between-person variance in steps.

Adding the predictor, fixed effect of within- and between-person *self-efficacy,* improved the model fit (*P*<.001) and explained 3.9% of the residual variance (individuals’ change over time) in steps. The fixed effect of within-person self-efficacy was statistically significant (*B*=462, 95% CI 296-628, SE 84; *P*<.001), suggesting that, when participants’ morning self-efficacy increased by 1, their daily steps increased by 462. The within-person perceived barriers were not statistically significant (*B*=−48, 95% CI −209 to 114, SE 82; *P*=.56; Table 9). Within-person pain or illness scores indicated that participants took, on average, 1524 steps less when they reported 1 score higher on a scale of 0 to 2 (*B*=−1524, 95% CI −2378 to 669, SE 435; *P*<.001). Parameter estimates for all variables in this model are shown in Table 9.

The fixed effect, within- and between-person morning *motivation*, improved the model fit and explained 2.7% of residual variance (individuals’ change over time) in steps. The fixed effect of within-person motivation was statistically significant (*B*=390, 95% CI 201-578, SE 96; *P*<.001), suggesting that, when participants’ morning motivation increased by 1, their daily steps increased by 390. Within-person perceived barriers were not statistically significant (*B*=−93, 95% CI −257 to 71; *P*=.27). Within-person pain or illness scores indicated that participants took, on average, 1828 steps less when they reported 1 score higher on a scale of 0 to 2 (*B*=−1828, 95% CI −2676 to –980, SE 431; *P*<.001). Parameter estimates for all variables in this model are shown in Table 10.

**Table 8:** Sequential multilevel models used to investigate the associations between self-efficacy (SE) and motivation (mot) and steps.

Model	Modeled parameters (*df*)	–2LL^a^ model deviance	Chi-squared (*df*) for change in model fit^b^	*P* value	BIC^c^
1	Fixed and random intercepts (1)	10,208.8	N/A^d^	N/A	10,227.6
2	Model 1+fixed time	10,203.2	5.6 (1)	.02	10,228.3
3	Model 2+random time	10,202.1	1.1 (1)	.29	10,239.8
4	Model 3+day of the week+barriers to PA^e^ (within and between persons)+pain or illness (within and between persons)	8288.6	1913.5 (10)	<.001	8385.7
5 (SE)	Model 4+self-efficacy (within and between persons)	8263.2	25.4 (2)	<.001	8372.4
6 (SE)	Model 5b+autocorrelation (within persons)	8255.4	7.8 (1)	.005	8370.7
5 (mot)	Model 4+motivation (within and between persons)	8270.3	18.3 (2)	<.001	8379.5
6 (mot)	Model 5a+autocorrelation (within persons)	8264.8	5.5 (1)	.02	8380.1

^a^–2LL: −2 log likelihood.

^b^Chi-squared test statistic and *df* derived from differences between model N and model N – 1 in deviance and *df*, respectively. Separate modeling processes examined the effects of self-efficacy and motivation on steps, but models 1 to 4 were statistically identical in both processes.

^c^BIC: Bayesian information criterion.

^d^N/A: not applicable.

^e^PA: physical activity.

**Table 9:** Parameter estimates for multilevel model of daily steps as a function of morning self-efficacy including wash-out days.

Type of effect	Variables in model	B (SE; 95% CI)	*t* test (*df*)	*P* value
Fixed	Intercept	10,328 (1190; 7816 to 12,839)	8.68 (17)	<.001
Fixed	Time	−23 (19; −63 to 18)	−1.20 (15)	.25
Fixed	Within-persons self-efficacy	462 (84; 296 to 628)	5.47 (402)	<.001
Fixed	Between-persons self-efficacy	263 (952; −1782 to 2309)	0.28 (14)	.79
Fixed	Within-persons perceived barriers	−48 (82; −209 to 114)	−0.58 (399)	.56
Fixed	Between-persons perceived barriers	926 (801; −792 to 2644)	1.16 (14)	.27
Fixed	Within-persons pain or illness	−1524 (435; −2378 to −669)	−3.51 (404)	<.001
Fixed	Between-persons pain or illness	−4059 (4630; −13,985 to 5867)	−0.88 (14)	.40
Random (between persons)	Intercept UN (1.1): intercept variance	15,897,177 (6,771,866; 6,898,043 to 36,636,511)	2.35	.02
Random (between persons)	UN (2.2): slope variance	−18,058 (83,791; −182,286 to 146,169)	−0.22	.83
Random (between persons)	UN (2.1): covariance	703 (1626; 8 to 65,553)	0.43	.67
Random (within persons)	Residual AR1^a^ diagonal	10,572,410 (775,140; 9,157,274 to 12,206,236)	13.64	<.001
Random (within persons)	Autocorrelation AR1 ρ	0.15 (0.06; 0.04 to 0.26)	2.79	.005

^a^AR1: autoregression of lag 1.

**Table 10:** Parameter estimates for multilevel model of daily steps as a function of morning motivation including wash-out days.

Type of effect	Variable in model	B (SE; 95% CI)	*t* test (*df*)	*P* value
Fixed	Intercept	10,286 (1106; 7960 to 12,612)	9.30 (18)	<.001
Fixed	Time	−27 (20; −69 to 16)	−1.35 (15)	.20
Fixed	Within-person motivation	390 (96; 201 to 578)	4.07 (297)	<.001
Fixed	Between-person motivation	1756 (921; −222 to 3734)	1.91 (14)	.08
Fixed	Within-person perceived barriers	−93 (83; −257 to 71)	−1.12 (383)	.27
Fixed	Between-person perceived barriers	708 (716; −828 to 2244)	0.99 (14)	.34
Fixed	Within-person pain or illness	−1828 (431; −2676 to −980)	−4.24 (397)	<.001
Fixed	Between-person pain or illness	−2244 (4111; −11,049 to 6562)	−0.55 (14)	.59
Random (between persons)	Intercept UN (1.1): intercept variance	13,175,341 (5,727,389; 5,620,049 to 30,887,561)	2.30	.02
Random (between persons)	UN (2.2): slope variance	1175 (1816; 57 to 24,306)	0.65	.52
Random (between persons)	UN (2.1): covariance	−36,902 (81,879; −197,382 to 123,578)	−0.45	.65
Random (within persons)	Residual AR1^a^ diagonal	10,772,153 (782,097; 9,343,342 to 12,419,461)	13.77	<.001
Random (within persons)	Autocorrelation AR1 ρ	0.13 (0.05; 0.02 to 0.23)	2.35	.02

^a^AR1: autoregression of lag 1.

## Discussion

### Principal Findings

No statistically significant differences were detected between steps on intervention and control days, neither in the N-of-1 analyses nor when the data were aggregated. The average of the aggregated steps on biofeedback intervention days was approximately the same as the control day average, and on dMI intervention days, the step average was somewhat lower than the control day average, although this difference was not statistically significant. These findings should be interpreted with caution because of the following features of the pilot trial.

First, the availability of self-regulatory BCTs may have reduced the intervention effectiveness. To maintain user interest in the app over time, the participants had continuous access to several self-regulatory BCTs also during control days (Table S1 in Multimedia Appendix 2). Meta-regressions have found a combination of self-monitoring and other self-regulatory BCTs to be effective in increasing physical activity [13] and intentions for physical activity [19]. Behavioral goal setting and self-monitoring were also found to increase steps in some participants in a factorial N-of-1 RCT [29]. Of the MI techniques used in this study, meta-analyses have found support only for the techniques “BCT 15.2: mental rehearsal of successful performance” in increasing intentions [19] and “SDT3: provide a rationale” in increasing autonomous motivation for physical activity [74]. These meta-analyses did not detect statistically significant effects of MI on autonomous motivation [19,74], intention, or stage of change [19]. Thus, the continuous availability of self-regulatory BCTs may have overridden the possible effects of the motivational interventions.

Second, the impact of the dMI intervention may have been diluted by the daily EMA questions as answering questions on motivation, self-efficacy, perceived barriers, and pain or illness also requires self-reflection on one’s motivation, capability, and opportunity to be physically active. Although the dMI intervention had more substantial content and used BCTs to evoke reflection on the reasons, life goals, and positive memories with physical activity [37], it is possible that users associated the daily EMA questions with the Precious app content more generally.

Third, the second intervention element, heart rate variability–based biofeedback, has the potential to be highly motivational content as it is personally relevant and responds immediately to changes in behavior [75-77]. The downside of this personal tailoring is that the content cannot be standardized or predetermined (ie, the “active ingredient” of the feedback may change). For instance, inactive participants may find it discouraging to see their activity levels not meet the recommendations, and participants with high stress scores may decide to focus on recovery instead of exercise. Thus, providing biofeedback to participants whose behavior and stress levels differ means that the intervention content also differs. Interestingly, anecdotal feedback in the follow-up interviews (not reported in this study) revealed that the participants may have actively varied their activity levels on biofeedback measurement days to receive a comprehensive picture of their well-being. Thus, the biofeedback measurements may have encouraged participants to even decrease their steps to receive a baseline reading on a recovery day.

Finally, the motivational interventions may also have met a ceiling effect. Despite self-reporting physical activity levels low enough to be included in the study, our participants were nevertheless those who had actively contacted the research team after seeing the newspaper advertisement, and they reported relatively high motivation and had high daily step averages overall. This possible ceiling effect and self-selection bias issue are a wider problem in physical activity promotion research.

The MI-based relational features in the Precious app aimed to support users’ need for relatedness [37] but did not seem to reach the effectiveness of face-to-face MI [39]. This is in line with the results of a meta-analysis that found that face-to-face delivery is a key factor in interventions targeting motivation and intention for physical activity [19]. Future studies are needed to investigate how digital interventions could approach the effectiveness of personal contact, exploring, for instance, the amount of contact [78] or the depth of engagement with intervention content [37]. The simple automated messages of the Precious app may not be perceived as MI, and more sophisticated, artificial intelligence–based solutions could improve the user experience and service effectiveness.

The observational analyses revealed that morning self-efficacy predicted daily steps in 27% (4/15) of the participants, whereas motivation predicted steps in 20% (3/15) of the participants. Self-efficacy and motivation were also statistically significant predictors when aggregating data from all participants. This acted as a validating element for the pilot trial data collection as daily steps and their predictors followed the theory-based hypotheses. It also provides further support to the models that suggest self-efficacy and motivation as key determinants of physical activity. The positive associations suggest that strong enough self-efficacy and motivation will help some individuals overcome everyday hurdles and find ways to add more steps to their everyday lives. Thus, self-efficacy and motivation remain central intervention targets.

Only 4 (29%) out of 14 participants showed an association between self-efficacy or motivation and steps when N-of-1 analyses were undertaken. For some, this may be explained by the low statistical power. These associations may also be hidden by time lags of varying lengths as motivation may not translate to physical activity immediately. Physical activity is time-consuming, and motivation can only translate to activity when environmental factors allow that [34]. For instance, participant 13 reported that her daily steps mainly depended on the availability of a car as her job included moving between different locations either by car or on foot. Exercising and sports may also require special clothing or equipment, and certain activities may be available only on certain weekdays, whereas others require a partner or team to play with. The perceived barriers variable may have controlled for some of these environmental factors. More accurate control variables might be created by interviewing participants on their personal barriers and facilitators before the start of the trial. Instead of daily steps, a more proximal outcome of increased motivation might be an increase in action planning [18]. Future studies could focus on detecting the effects of motivation on planning and follow the enactment of these plans.

Individual differences were found in the associations between the EMA predictors and steps. For participants 13 and 14, even high motivation could not overcome the impact of perceived barriers and pain or illness. For participants 7 and 14, pain or illness may have decreased daily steps independently of their motivation, but the effect was not detected when self-efficacy was included in the model, suggesting that their beliefs about their capability to be active could provide a better estimate of their daily activity than their desire to be active. Interestingly, participant 14 seemed to take more steps on days with higher self-efficacy but also on days with higher perceived barriers. A mixed methods study could identify possible reasons behind these associations by interviewing participants using their data as a starting point.

This trial aimed to test the immediate impact of specific intervention elements on individuals and did not hypothesize a lasting change in an individual’s steps over time. The data showed a slight overall decrease in steps over time, possibly influenced by the timing of the intervention as weather conditions often become increasingly challenging in Finland from October to December [79].

### Lessons Learned in the Delivery and Measurement of the N-of-1 RCT

This feasibility study revealed many useful considerations for future studies in this emergent field of within-person RCTs.

#### Challenges Related to Daily Steps as a Measurement Unit

The Precious trial found a high variation in step counts between individuals (57%), which is in line with previous N-of-1 research [29]. However, 43% of the variance was detected within individuals. In this study, the difference between intervention and control days was approximately 2000 steps for several participants, but this difference did not reach statistical significance. Similarly, in a previous N-of-1 trial [29], intervention and control day differences as high as 1500 steps did not reach statistical significance. As steps vary over time from hundreds to tens of thousands of steps per day, large intervention effects would be needed to create step increases that are statistically detectable from this naturally high day-to-day variation. The problem of low power is typical in the field of highly personalized digital interventions; for example, “just in time adaptive intervention” studies have not been powered to detect intervention effects [6].

Steps are also a challenging outcome measure as connectivity errors or activities performed without the activity bracelet can lead to thousands of steps’ worth of missing data. In this study, although we instructed participants to wear the device at all times, we were unable to assess device wear time, and periods of nonwear may have biased the results for some users. For a more reliable study of daily activity, advanced modeling with, for example, heart rate data may be necessary to identify whether participants are inactive or whether the data are missing. In this trial, steps were only collected using the activity bracelet, and the manually logged activities were not translated into daily steps, as conceptualized in the study by Nurmi et al [37]. This may have biased the outcomes of participants who engaged in physical activity that does not accumulate steps, such as resistance training.

A third challenge related to daily steps is that the variation in steps can have a natural compensatory fluctuation—days with high activity may be followed by recovery days with low activity. Therefore, 2 consecutive intervention days may not be optimal for physical activity N-of-1 RCTs. A solution to this might be to change the analysis units from daily activity to a more dynamic observation, for instance, monitoring the frequency and height of peak activity days. Averaging physical activity scores over a week (eg, with rolling 7-d averages) or modeling the number of days since the previous intervention day could also be used to account for the cyclical nature of physical activity.

Another consideration with daily steps is determining the start and end of a day for each participant. Interventions and measurements scheduled daily may be influenced by changing working hours in certain jobs that involve night shifts. Late nights out may also add a significant number of steps after midnight. When studying the impact on daily steps, the time when the day is cut off may thus affect the results. The data in this study were collected during the period of midnight to 11:59 PM each day, but another approach would be to dynamically collect the data until an extended period of inactivity during the night (or a personally determined period for individuals with exceptional circadian rhythms).

#### Challenges of the N-of-1 RCT: Tailored Content

Precious was designed as a tailored, interactive motivational service that suggests relevant BCTs based on users’ preferences and motivational stage [37]. The use-based recommendations were switched off during the trial as an N-of-1 RCT requires randomizing the timing of repeated intervention elements. This may have affected the effectiveness of the Precious app as some core elements targeting autonomy, relatedness, and intrinsic motivation were not available. For instance, gamified features often include surprises and achievement-based rewards, but this type of reactive elements fit the N-of-1 RCT design poorly. Thus, future tests of the Precious system would need to be conducted using a research design that allows for the use of interactive motivational content. One option is the “changing criterion design” [80], in which a new intervention feature is introduced when participants reach a certain level in the outcome variable.

#### Considerations Regarding the N-of-1 RCT: Factorial Design

The notice of a new intervention period and feedback on goal progress were delivered via smartphone notifications. Some participants reported missing some notifications or seeing only the beginning of the text in the smartphone notifications. This feedback led to the development of a library of notifications on the Precious app after the trial had finished so that future users could access their earlier messages anytime. The high importance of timely intervention delivery in the factorial N-of-1 RCT design would lead us to encourage the use of repeated notifications until the message has been marked as received.

In addition, when assigning randomly timed interventions to individuals, some participants may end up with long periods without an intervention. For instance, several Precious trial participants had a 14-day gap between 2 dMI sessions (however, they received biofeedback interventions during that time). The possible long gaps and irregularity of the intervention elements may in fact diminish participants’ likelihood of tiring of the intervention elements. N-of-1 RCTs are not ideally suited to interventions that require a certain frequency of intervention exposure and are best suited to interventions with rapid- rather than slow-onset effects and limited carryover effects [61].

### Strengths

To our knowledge, this study is the first to test smartphone delivery of dMI features in a fully automated factorial N-of-1 RCT. Both approaches are important avenues for future research—they incorporate the possibilities that new technologies offer for personalized, ubiquitous support to individuals and for rapid testing of the impact of several intervention components in small study populations. Such solutions with reduced costs and easy delivery are needed to tackle major public health challenges.

The procedures of this pilot field trial were acceptable judging by the high intervention uptake and high participant adherence to the activity bracelet and daily EMA measurements. All participants (15/15, 100%) completed the trial, which is in line with other N-of-1 studies lasting a maximum of 3 months with no dropout [27,29,81] or a low dropout [28,82].

The data collection strategy was another strength of this trial as the cognitive correlates of physical activity were collected in a real-life environment and physical activity was objectively measured. Daily EMA measurements minimized the biases associated with retrospective questionnaires. The interventions in the Precious app were multifaceted, including techniques from MI and physiological, heart rate variability–based biofeedback to increase the salience of the immediate consequences of behavior on physiological well-being. Unlike interventions delivered in person, smartphone delivery with a modular study design allowed for testing for specific, isolated intervention techniques and their immediate impact on behavior. This study design may help determine the “active ingredients” in interventions and, thus, advance the understanding of behavioral determinants. This approach could also help determine the individual “dosage” that each user needs [5,83,84]. The use of digital technology also helped track the exact timing of the interventions and recognize that some biofeedback measurements were conducted outside the suggested times.

This factorial N-of-1 RCT with the Precious app showed high acceptability and adherence in an ecologically valid setting. The detected daily within-person associations among self-efficacy, motivation, and steps provided further support for central behavior change theories but also highlighted the possible differences between individuals as these associations were only detected in less than one-third of participants. In addition, this pilot study identified several suggestions to improve the implementation of future N-of-1 RCTs, which come with their specific challenges [61].

### Limitations

The automated N-of-1 delivery was mainly successful. Delivery of the dMI elements of the Precious app was deemed acceptable and feasible, whereas the biofeedback interventions faced some technical and practical challenges. As 27% (4/15) of the participants missed some biofeedback measurement days, smartphone notifications alone seem to have been an insufficient nudge to start the measurement. As N-of-1 RCTs are very sensitive to the accurate timing of interventions [61], future studies using app-controlled intervention delivery need to ensure that notifications are received on time, possibly using sound and vibration alarms in addition to text-based notifications.

This study conducted intention-to-treat analyses studying whether intervention *availability* is associated with increased step count. Participants typically only engaged with the dMI interventions on the first day they were available. Owing to the pilot nature of the study, the relatively low number of days of engagement with the service, and the spillover of the biofeedback measurements, no per-protocol analyses were conducted. Future studies could explore whether the days in which participants actively engaged with the intervention materials are associated with changes in behavior as behavior change interventions are most effective for people who actively use the available BCTs [85-87].

To minimize participant burden and the risk of confounding motivational effects, all EMA questions were single items [88]. However, single items offer no same-day reference points for imputing missing values. As participants showed high adherence to the twice-a-day EMA measurement [89], future studies could consider assessing more motivational variables that, for example, distinguish qualitatively different motivations from SDT [49].

This study used a factorial design to test 2 different interventions in a short field trial. The duration of this trial was limited to 40 days, primarily because of the estimated battery life of the accelerometers used to collect step data. This means that the study was underpowered to assess the interaction effects of the 2 interventions and, according to recent evidence [90], perhaps even underpowered to study the effects of single interventions in which longer time-series data are needed.

### Conclusions

This paper presented an automated N-of-1 factorial RCT delivered via smartphone testing 2 types of physical activity interventions: dMI and heart rate variability–based biofeedback. High intervention uptake and high adherence to daily EMA measurements indicated a good level of acceptance of the Precious app and the automated factorial N-of-1 design, but no intervention effects were found. Daily self-efficacy and motivation were associated with daily steps in 27% (4/15) and 20% (3/15) of the participants, respectively, and in the aggregated data from all participants. The novel use of randomly timed, preprogrammed smartphone notifications for the delivery of the intervention components may decrease the risk of human errors in intervention allocation or data collection. The automated delivery may be sensitive to other challenges, such as missed smartphone notifications and technical problems. With careful selection of intervention content and improved focus on the uptake of digital interventions during the allocated intervention days, an automated N-of-1 RCT can become a valuable tool for testing the impact of specific intervention techniques.

## Appendix

Multimedia Appendix 1 Relational and behavior change techniques that were available throughout the trial.

Multimedia Appendix 2 Technical challenges during the trial.

Multimedia Appendix 3 Days of the trial in which participants either set a step goal (1) or did not set a step goal (0) or for which data were missing (hyphens).

Multimedia Appendix 4 Step count graphs for all trial participants, with separate trend lines for control and active intervention days.

Multimedia Appendix 5 CONSORT-eHEALTH checklist (V 1.6.1).

## Abbreviations

BCT: behavior change technique
CENT: Consolidated Standards of Reporting Trials Extension for reporting N-of-1 Trials
dMI: digitalized motivational interviewing
EMA: ecological momentary assessment
ICC: intraclass correlation coefficient
MI: motivational interviewing
RCT: randomized controlled trial
RQ: research question
SDT: self-determination theory

## Data Availability

The data sets generated and analyzed during this study are available from the Open Science Framework [91].
