# Supplementary material for: Biofeedback and Digitalized Motivational Interviewing to Increase Daily Physical Activity: Series of Factorial N-of-1 Randomized Controlled Trials Piloting the Precious App
Source: JMIR Form Res. 2023 Nov 23;7:e34232. doi: 10.2196/34232 (PMC10704305; doi:10.2196/34232)
Supplement: Multimedia Appendix 1 [file formative_v7i1e34232_app1.docx]

*Supplementary table 1.* Relational techniques and behavior change techniques that were available through the trial.

| **Relational technique as in** Hardcastle et al. (2017) | **Available during MI interventions** | **Available during biofeedback interventions** | **Available during control days** | **Description** |
| --- | --- | --- | --- | --- |
| T1.1: Open-ended questions | Y |  |  | Asking questions that cannot be answered with a single-word answer (e.g., yes, no, rarely). |
| T1.2: Affirmations | Y | Y | Y | Acknowledging users’ self-worth and efforts. |
| T1.3: Reflective statements | Y | Y |  | Rewording the selections user has made. |
| T3.16: Emphasize autonomy | Y | Y | Y | Freedom to select outcome goals, behavioral goals, activities and their timing. |
| T3.22: Normalizing | Y | Y |  | Acknowledging that it is not uncommon to find it difficult to engage in new behaviors. |
| T4.2: Consider change options | Y |  |  | Using neutral language and supporting the user to consider different options. |
| T4.5: Support change | Y |  |  | Acknowledging users’ ability to select best options for them and reminding them of the selections they’ve made. |
| **Behavior change techniques (BCTs) as in** Michie et al. (2013) |  |  |  |  |
| 1.3 Outcome goal setting | Y |  |  | Prompting users to reflect on their life goals and to choose their preferred outcome goal(s) from a list. |
| 1.7 Review outcome goals | Y |  |  | Asking if the user is happy with their previous outcome goals or wants to set a new goal. |
| 15.2 Mental rehearsal of successful performance | Y |  |  | Asking users to visualize a future event in which they would enjoy physical activity and prompts them to reflect on the positive consequences of this experience. |
| 15.3 Focus on past success | Y |  |  | Asking users to reflect on a past event in which they enjoyed physical activity and prompting them to reflect on the positive consequences of this experience. |
| Additional BCT: Linking behavioral goals with outcome goals | Y |  |  | Reminding users that their behaviors (e.g. tidying, dancing) can help them to achieve their outcome goals (e.g. reducing stress, feeling connected to others). |
| 10.4 Social reward | Y | Y | Y | Delivering smartphone notifications with encouraging messages based on tracked service engagement or activity. Biofeedback reports acknowledge and celebrate progress. |
| 1.1 Behavioral goal setting | Y | Y | Y | Suggesting a daily step goal. To set an achievable goal, users see their past seven-day average + 5% as a starting point. |
| 1.4 Action planning* | Y | Y | Y | Planning tool lets the user to choose activity type, intensity and timing. |
| 1.5 Review behavior goal(s) | Y | Y | Y | The self-monitoring tool displays earlier goals, when those were achieved, and allows to adjust the goal for the current day. |
| 1.6 Discrepancy between current behavior and goal | Y | Y | Y | Users receive smartphone notifications on the step % that they have accomplished by afternoon. Their goal progress is continuously visible in the self-regulation tool. |
| 2.2 Feedback on behavior | Y | Y | Y | A smartphone notification on progress towards user’s step goal and goal achievement is sent every afternoon. |
| 2.3 Self-monitoring of behavior | Y | Y | Y | The self-regulation tool displays the steps tracked with the activity bracelet in the past three days. Users can manually log other activities than walking, running, and cycling (which are automatically tracked). Previous action plans can be checked completed with a single tap. |
| 2.4 Self-monitoring of  outcome(s) of behavior |  | Y |  | Firstbeat biofeedback report provided visualizations and verbal feedback on rest, recovery, stress levels, and physical activity. Participants could use these to monitor how their body’s reactions change over time. |
| 2.6 Biofeedback |  | Y |  | Informing the participants about their heart-rate variability-based physical activity levels, stress levels, and sleep quality. |
| 2.7. Feedback on outcome(s)  of behavior |  | Y |  | Firstbeat biofeedback report provided visualizations and verbal feedback on rest, recovery, stress levels, and physical activity. |
