# Supplementary material for: Biofeedback and Digitalized Motivational Interviewing to Increase Daily Physical Activity: Series of Factorial N-of-1 Randomized Controlled Trials Piloting the Precious App
Source: JMIR Form Res. 2023 Nov 23;7:e34232. doi: 10.2196/34232 (PMC10704305; doi:10.2196/34232)
Supplement: Multimedia Appendix 2 [file formative_v7i1e34232_app2.docx]

*Supplementary table 2.* Technical challenges during the trial.

| Participant | Motivational interviewing interventions | Firstbeat biofeedback interventions | Daily EMA questions | Other | Outcome |
| --- | --- | --- | --- | --- | --- |
| P01 | - | - | Received the first EMA measurement notification in Dutch instead of Finnish | - | The provider corrected the language |
|  | - | Used the Firstbeat device for 4 extra days at the beginning of the trial. | - | - | Received instructions by email and started using Firstbeat during assigned intervention times |
|  | - | Could not access the Firstbeat report after the first measurement because of an extra space at the end of username | - | - | Username fixed and access to the following reports restored |
| P02 | - | - | - | Activity bracelet not communicated step data to the phone for 19/40 days only | Data received only for 21 days in the middle of the trial |
|  | - | Could not access the first biofeedback report and the second measurement was lost for unknown technical reason | - | - | Had the sensor replaced and could access following reports |
| P03 | - | Conducted the first measurement a day late. | - | - |  |
|  |  | Did not receive/see the last two notifications to conduct biofeedback measurements |  |  |  |
| P04 | - | Only conducted two biofeedback measurements, both during control days, and did not manage to download the reports to her computer | - | - | Participant received the reports after the trial and thus did not get any biofeedback during intervention days |
| P05 | - | - | - | - |  |
| P06 | - | - | - | - |  |
| P07 | - | - | - | - |  |
| P08 | - | Conducted the sixth measurement a day late | - | - |  |
| P09 | - | Reported that one biofeedback measurement did not identify her high intensity training as vigorous activity | - | No step data recorded on the phone or the server |  |
| P10 | - | Conducted the third measurement a day late | - | - |  |
| P11 | - | Was not able to download the biofeedback reports during the trial | Did not receive EMA questions during the first trial day | - | Conducted 6/7 biofeedback measurements during the assigned times but received the reports at the follow-up meeting |
| P12 | - | Could not log into the biofeedback server during the trial | - | - | Biofeedback reports lost |
| P13 | - | - | - | No usage data on the server | Biofeedback reports lost |
| P14 | - | - | - | No usage data on the server | Biofeedback reports lost |
| P15 | - | - | - | - | - |
