# Supplementary figures and images for: Biofeedback and Digitalized Motivational Interviewing to Increase Daily Physical Activity: Series of Factorial N-of-1 Randomized Controlled Trials Piloting the Precious App
Source: JMIR Form Res. 2023 Nov 23;7:e34232. doi: 10.2196/34232 (PMC10704305; doi:10.2196/34232)

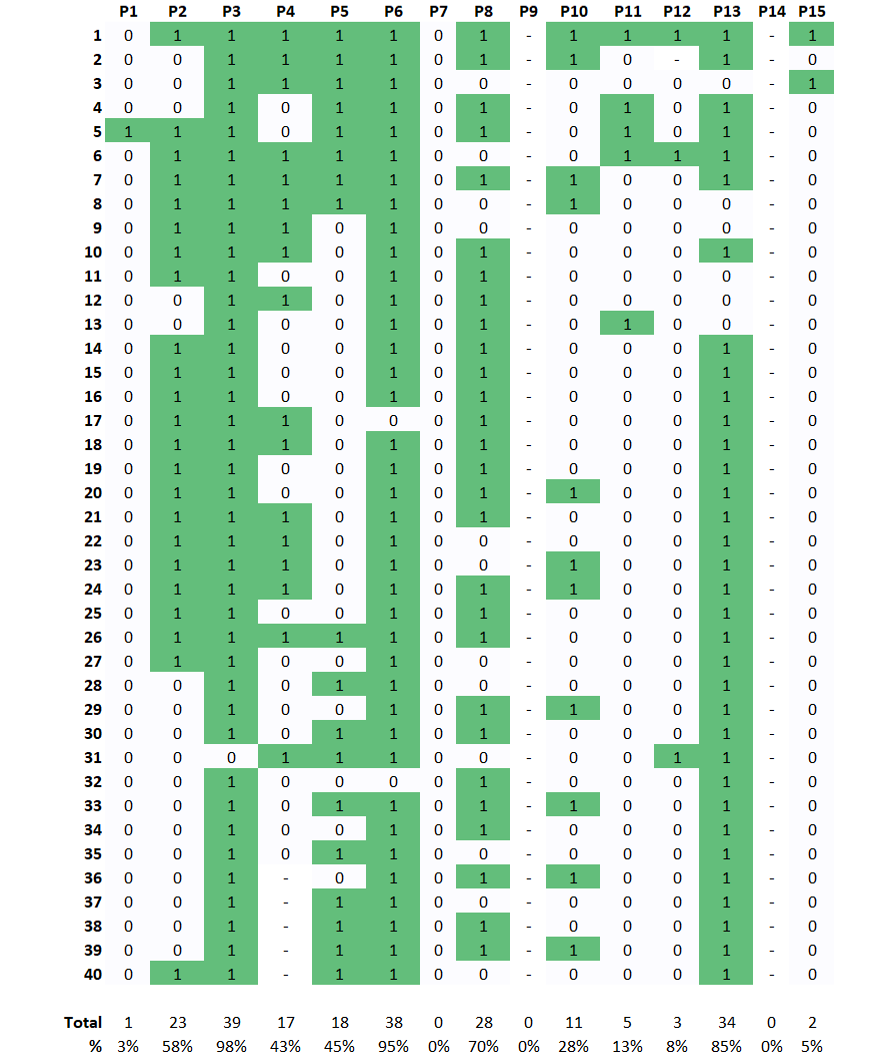

Supplement: Multimedia Appendix 3 [file formative_v7i1e34232_app3.png]
