# Supplementary material for: Biofeedback and Digitalized Motivational Interviewing to Increase Daily Physical Activity: Series of Factorial N-of-1 Randomized Controlled Trials Piloting the Precious App
Source: JMIR Form Res. 2023 Nov 23;7:e34232. doi: 10.2196/34232 (PMC10704305; doi:10.2196/34232)

Participant 1

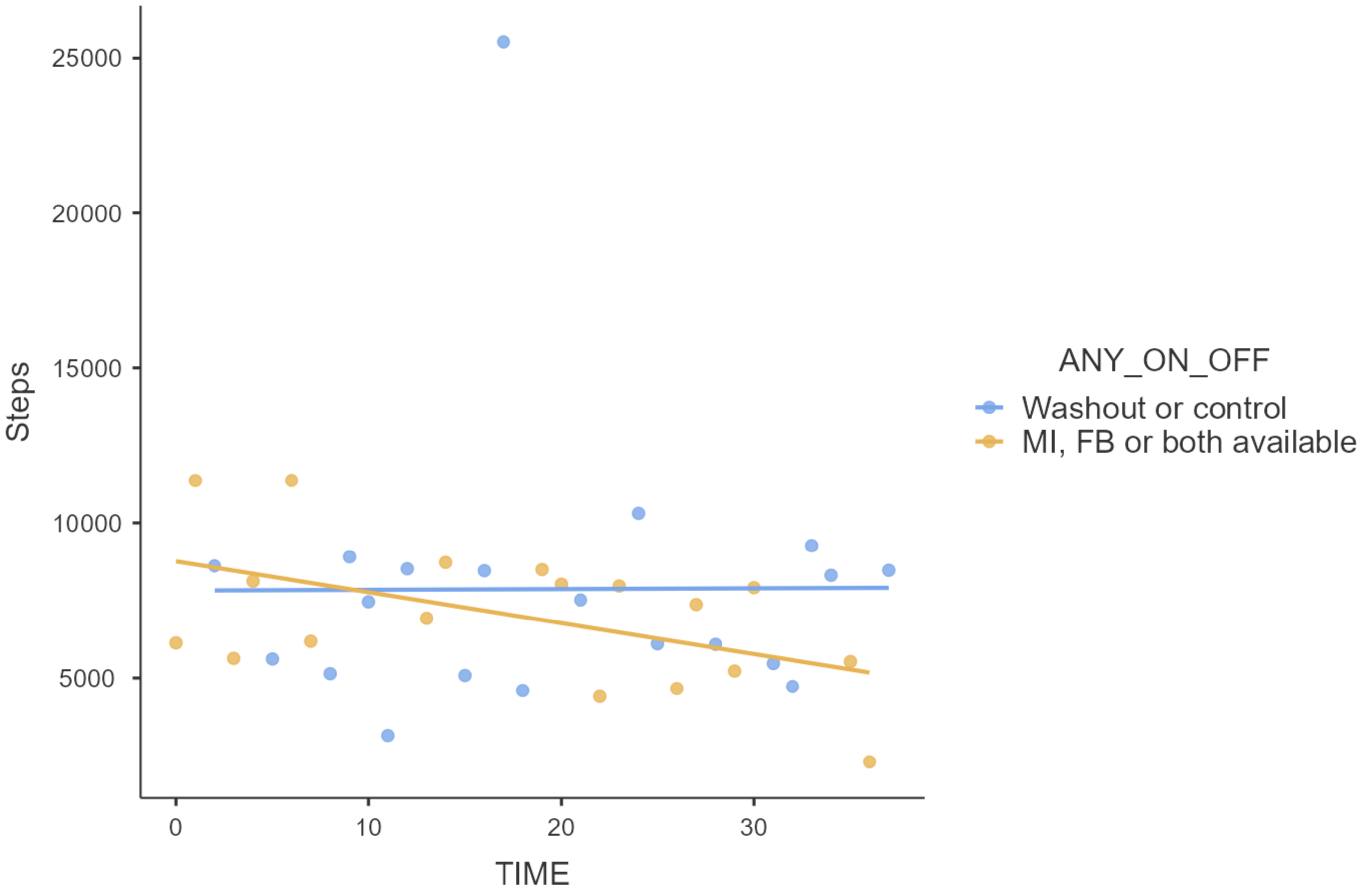

Participant 2

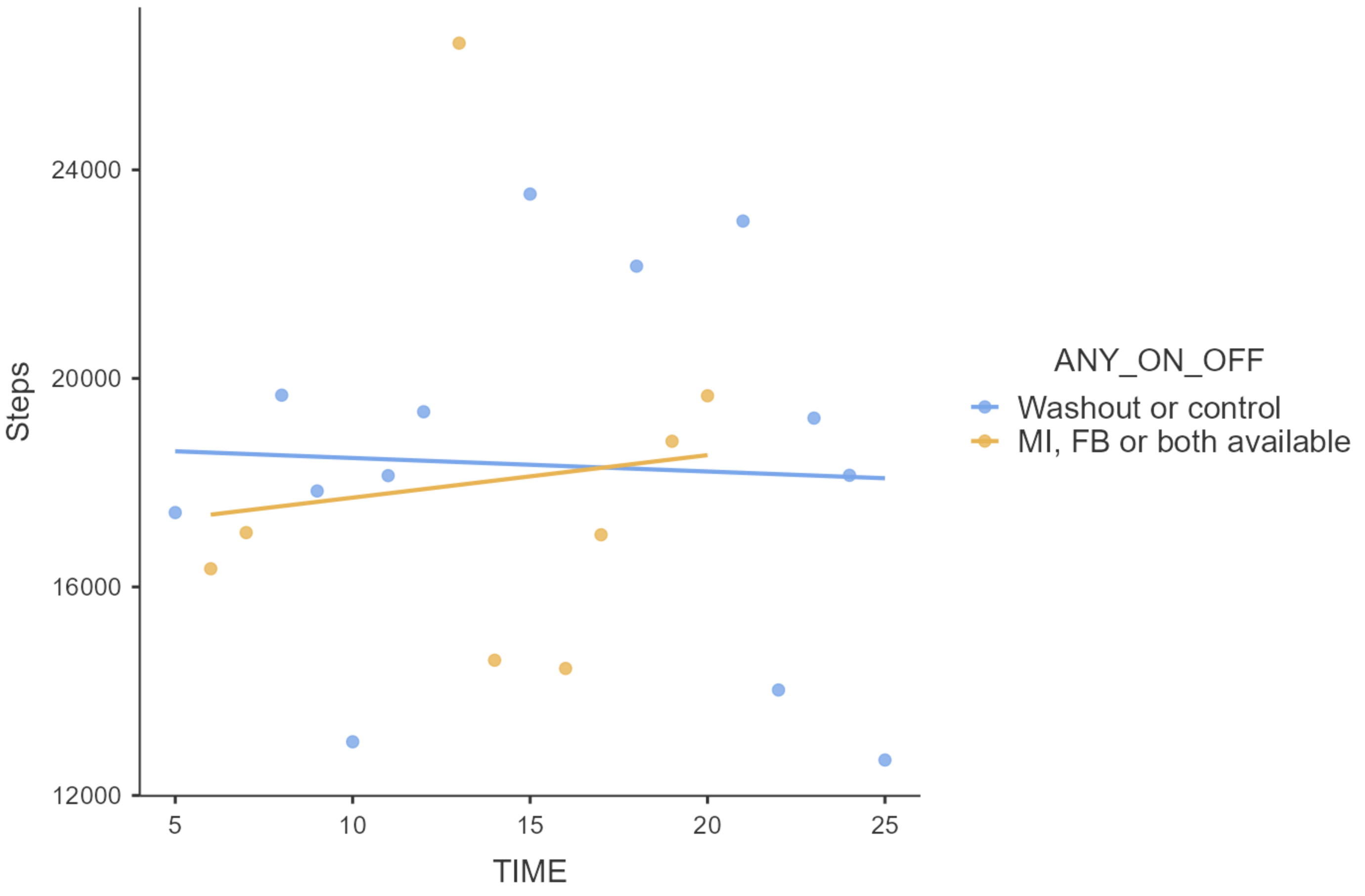

Participant 3

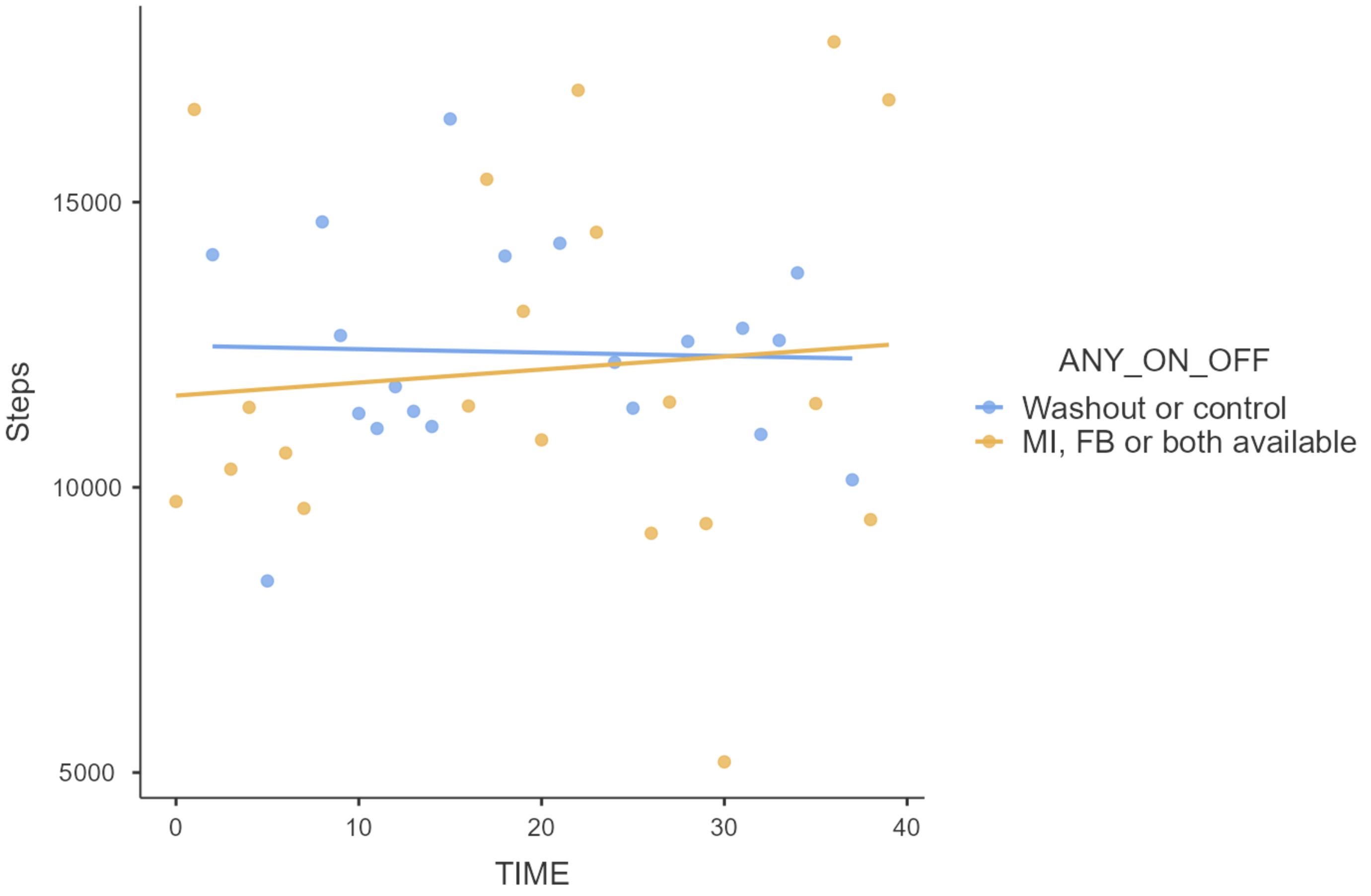

Participant 4

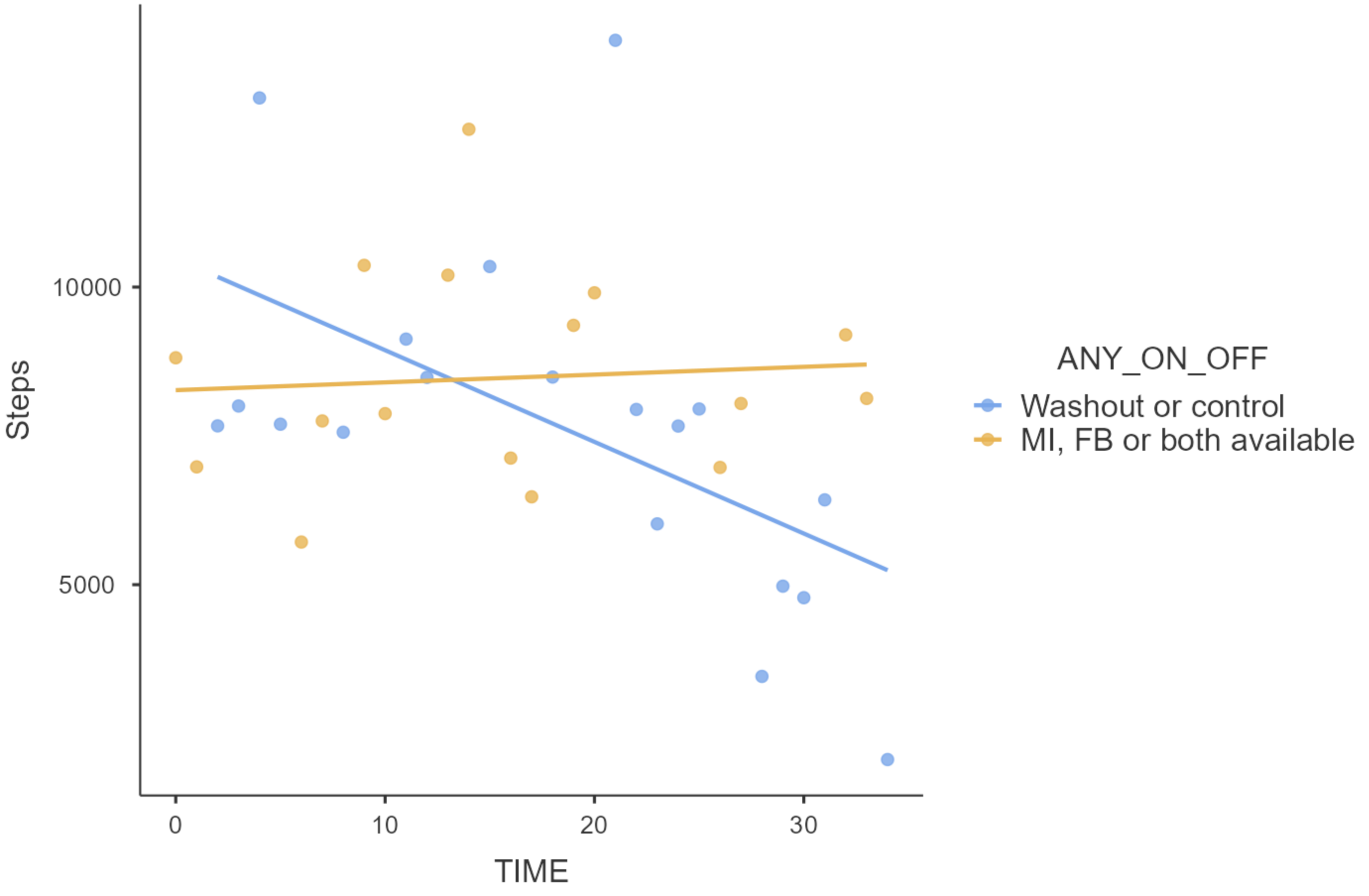

Participant 5

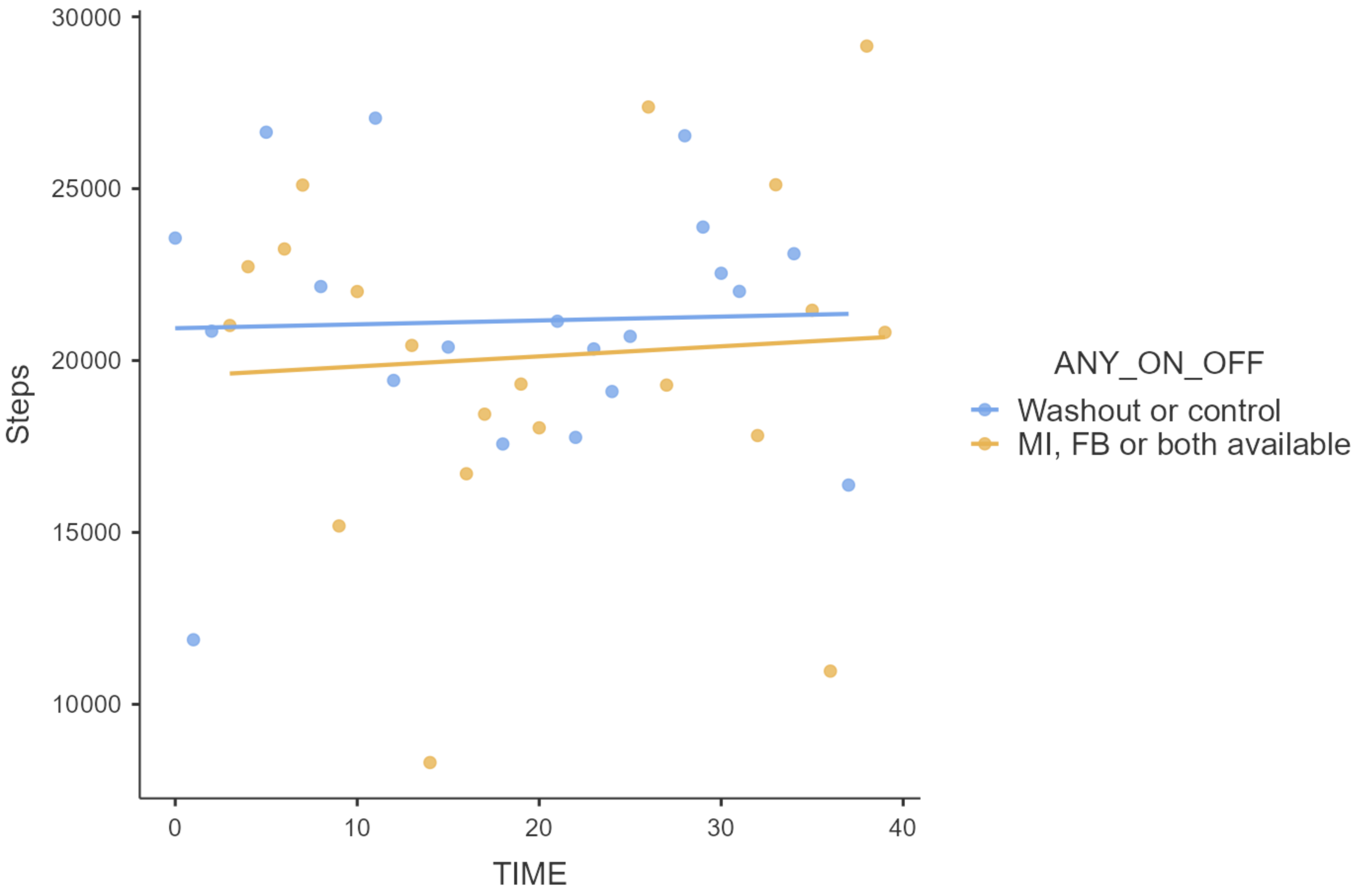

Participant 6

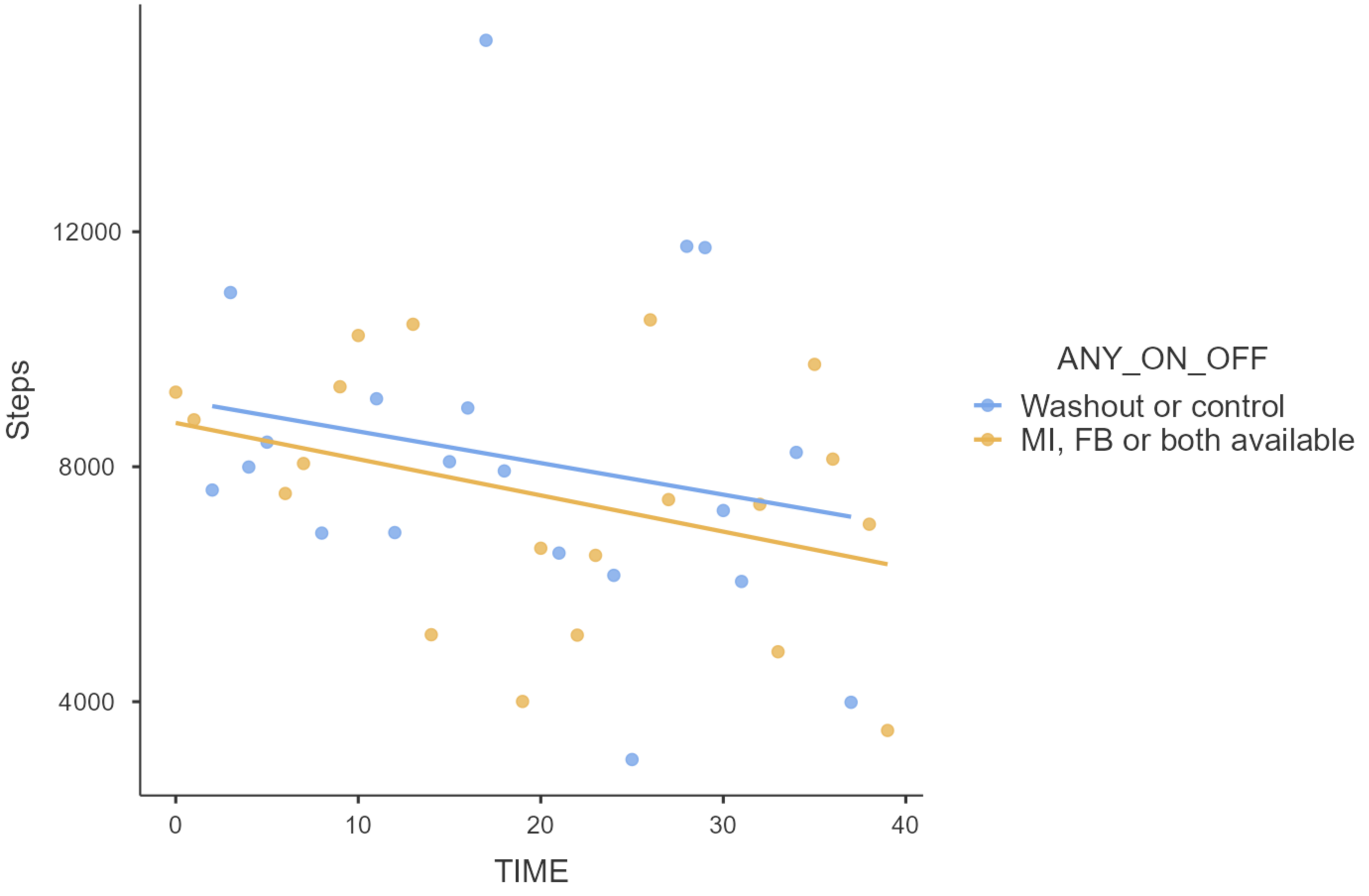

Participant 7

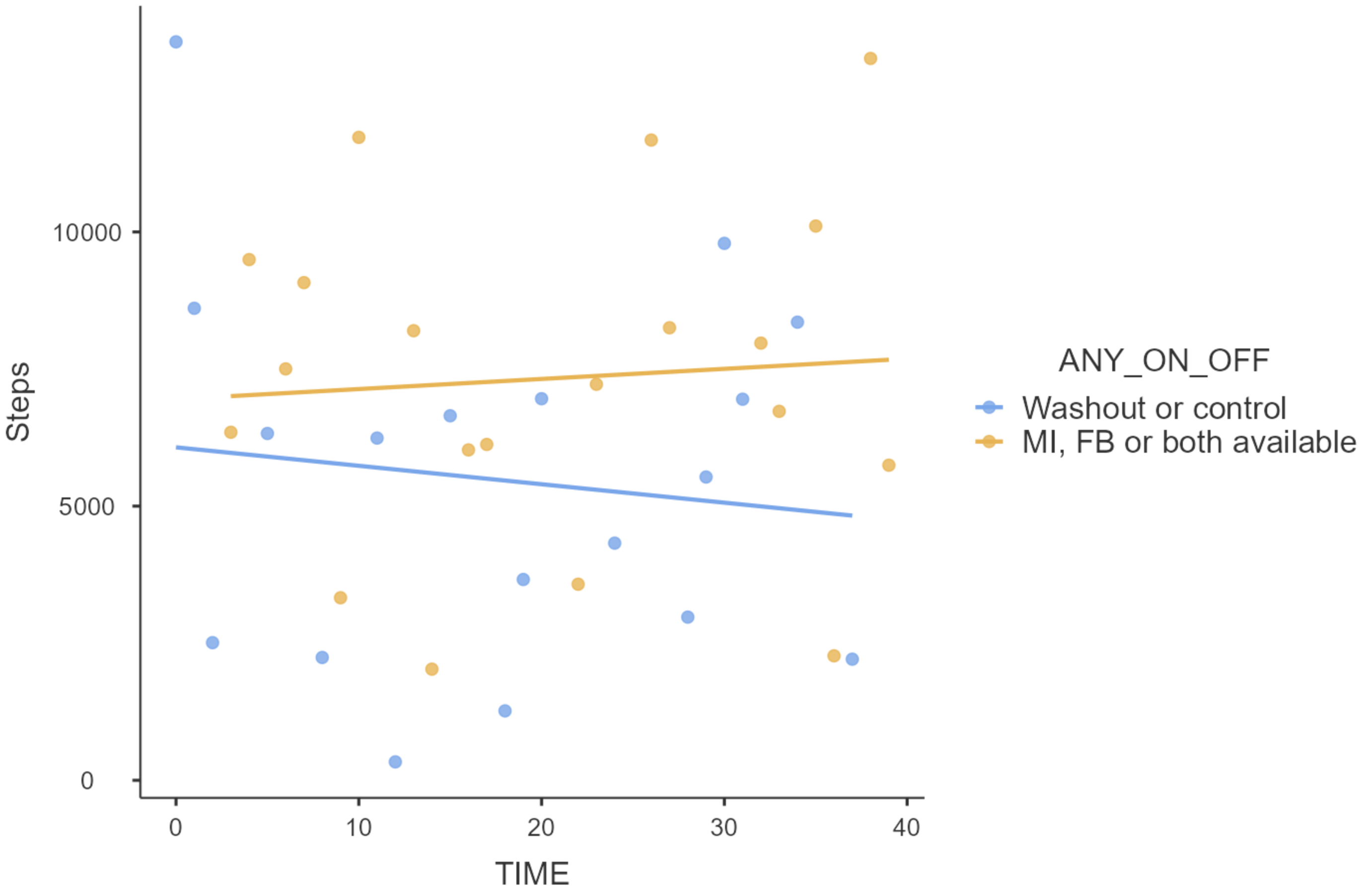

Participant 8

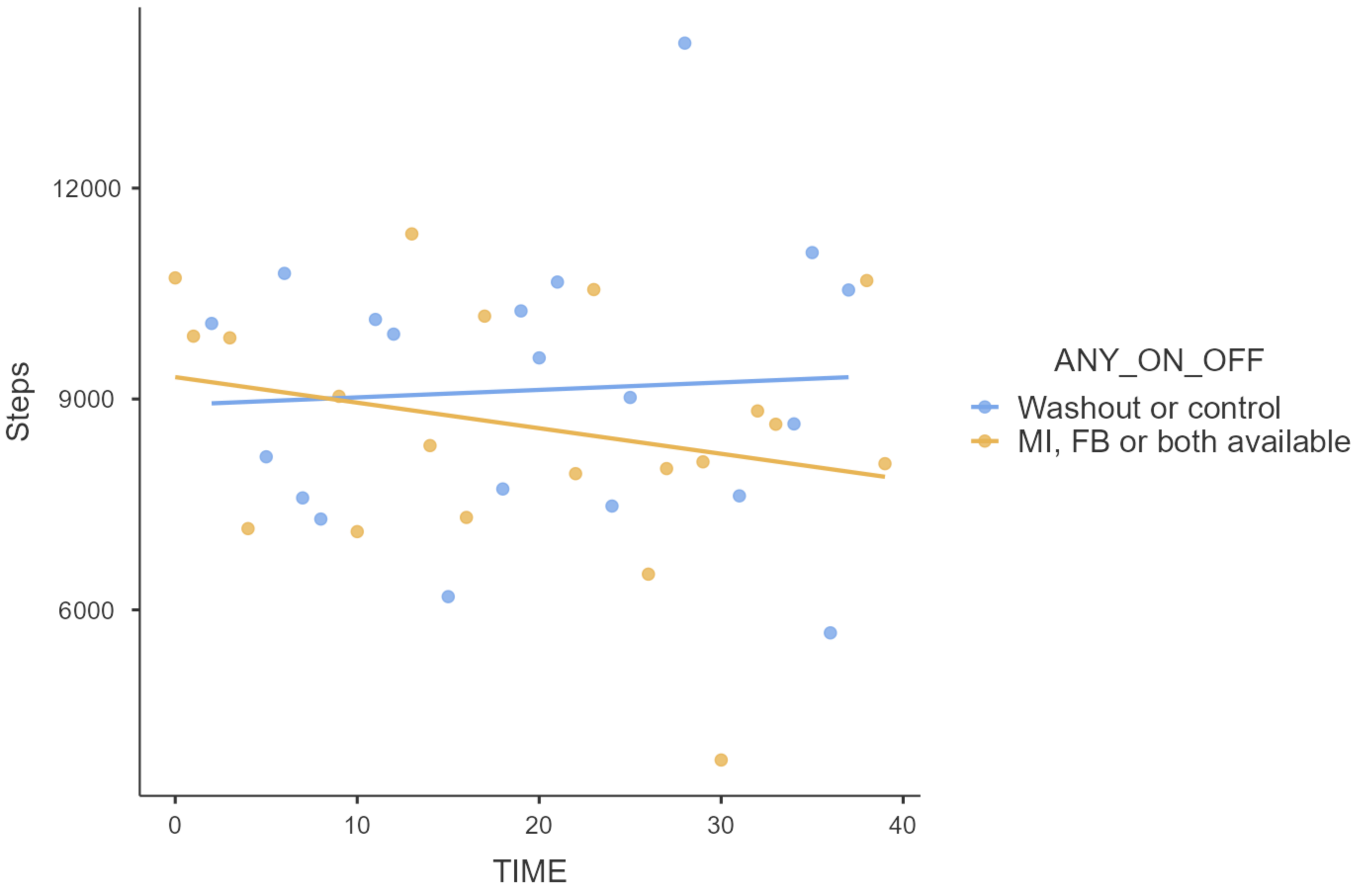

Participant 10

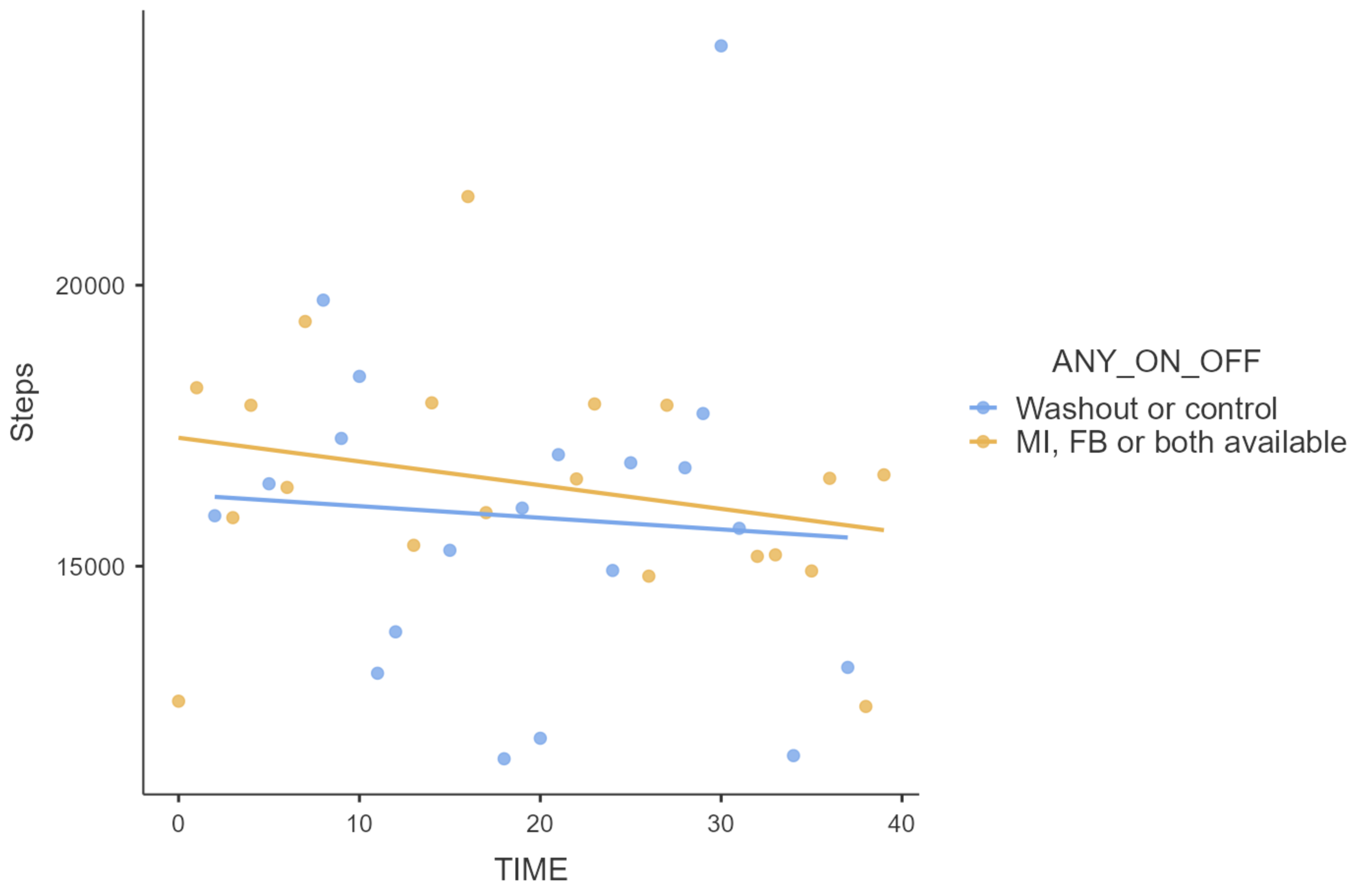

Participant 11

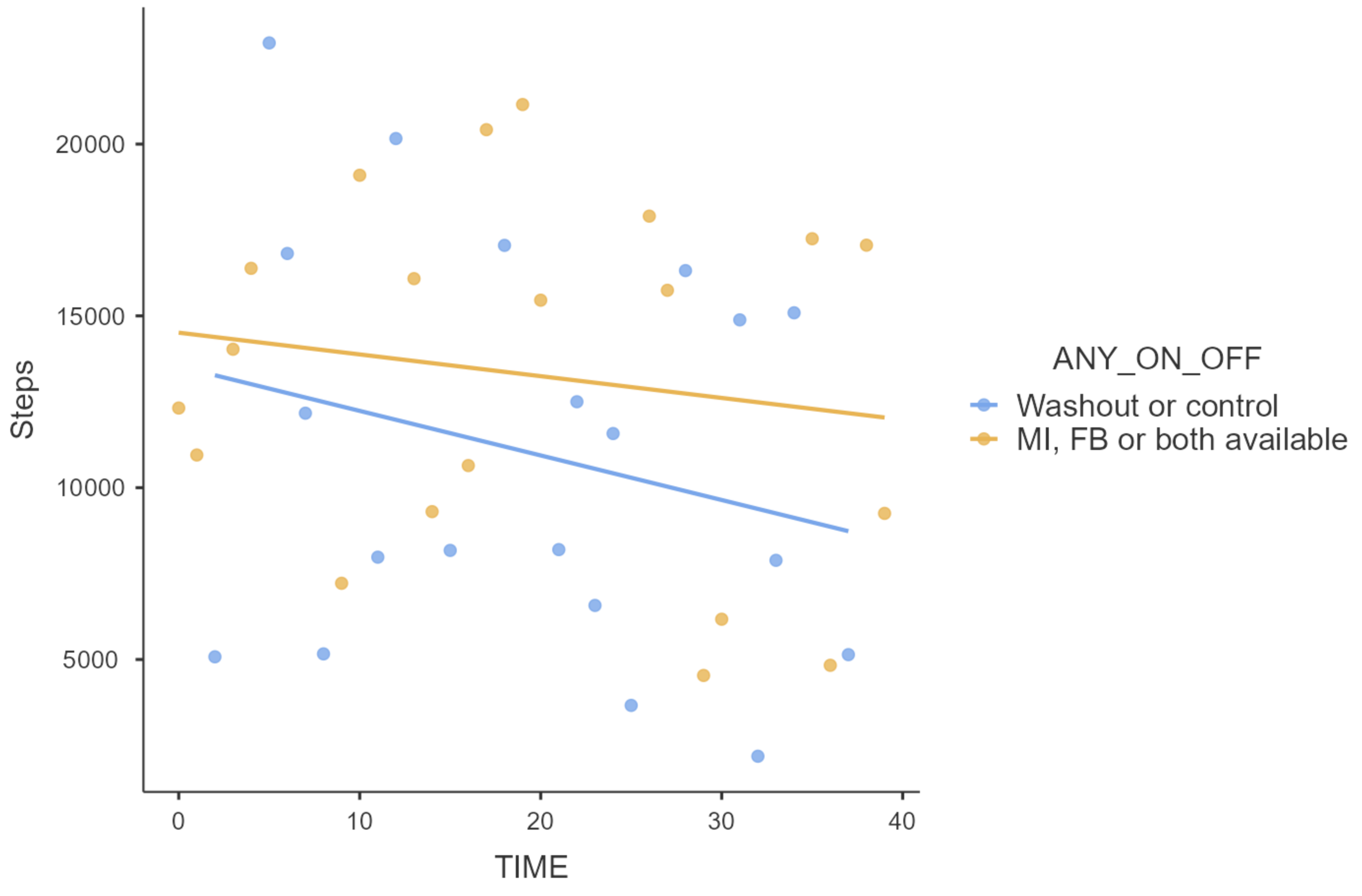

Participant 12

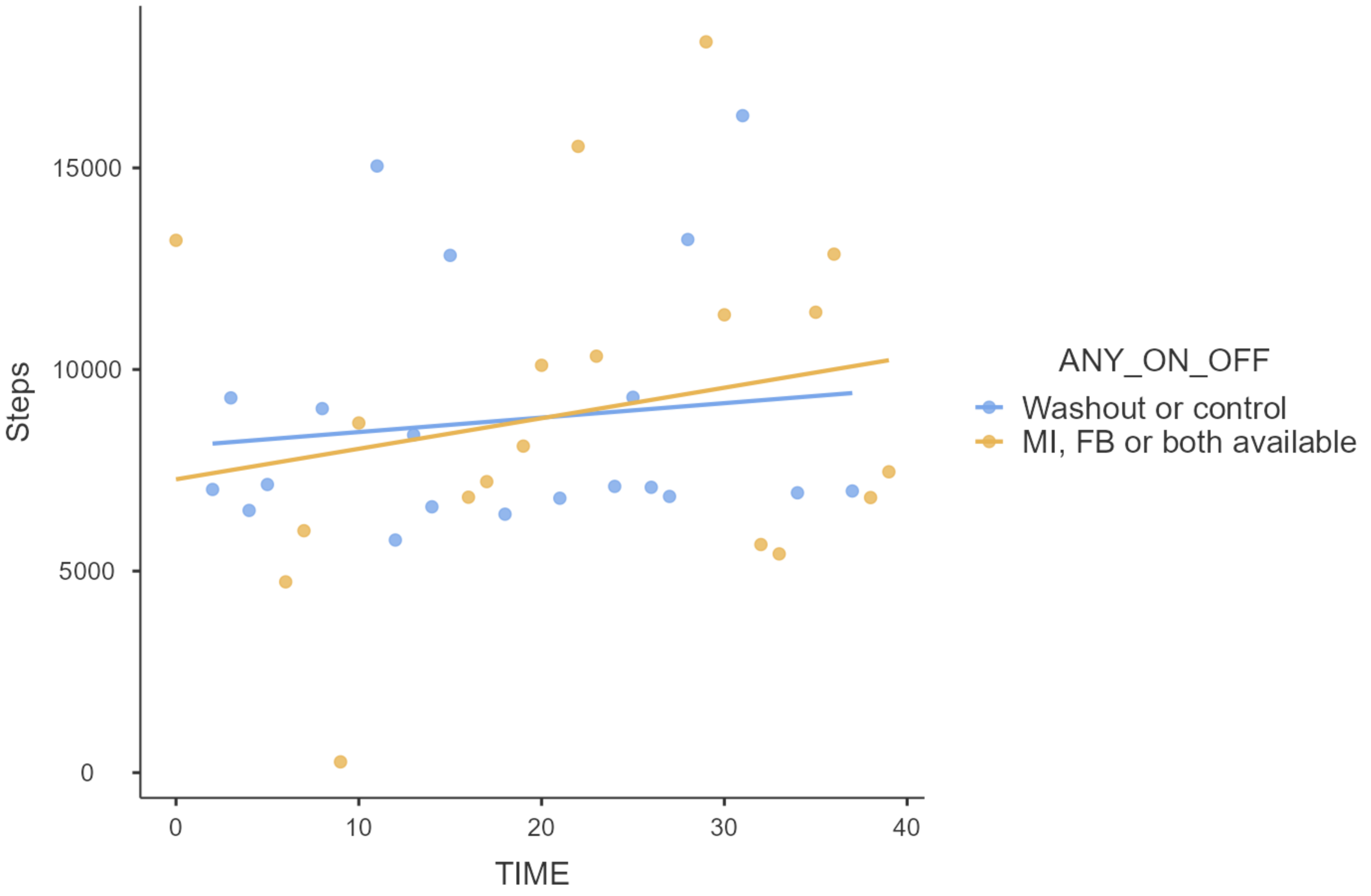

Participant 13

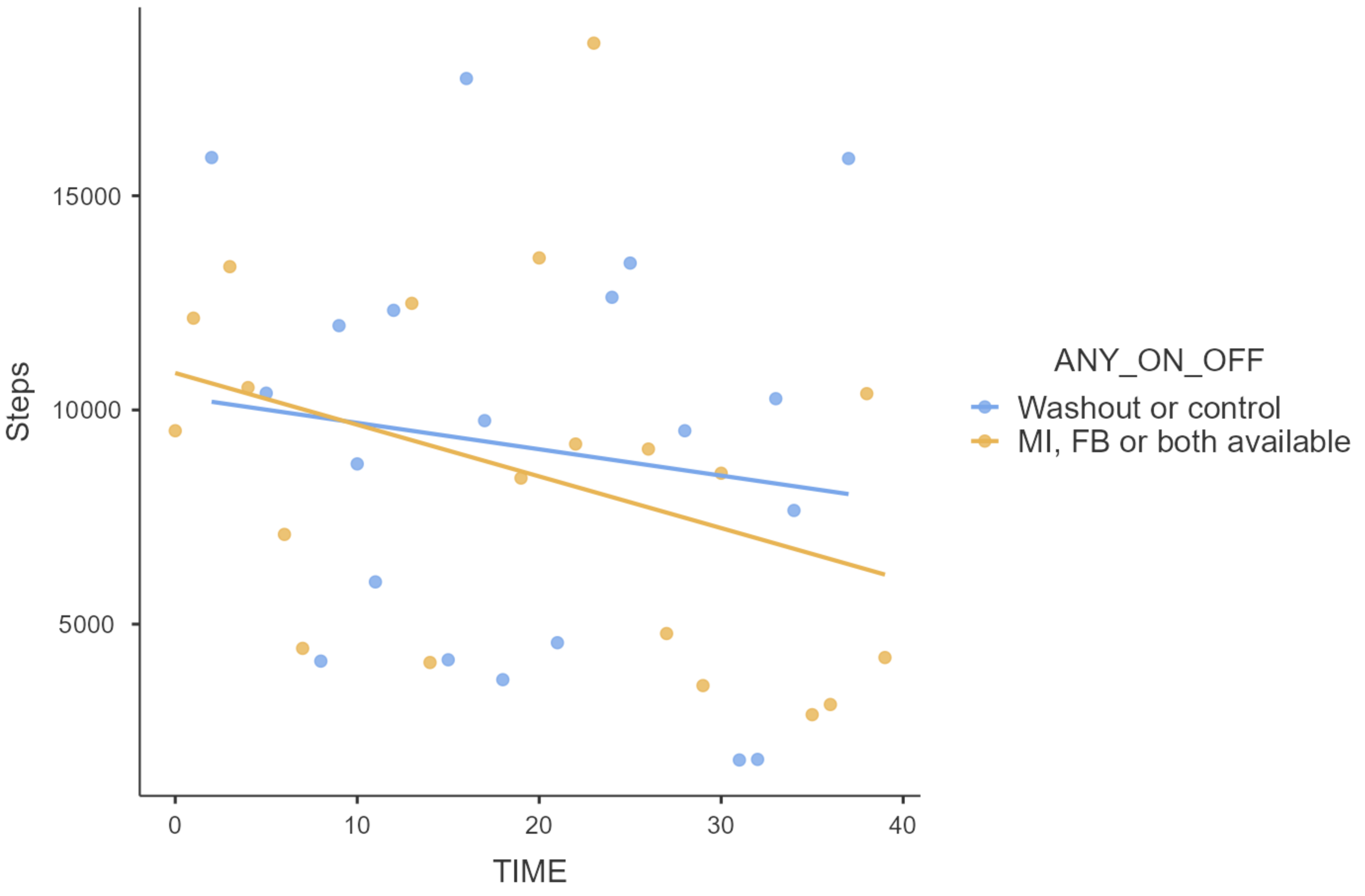

Participant 14

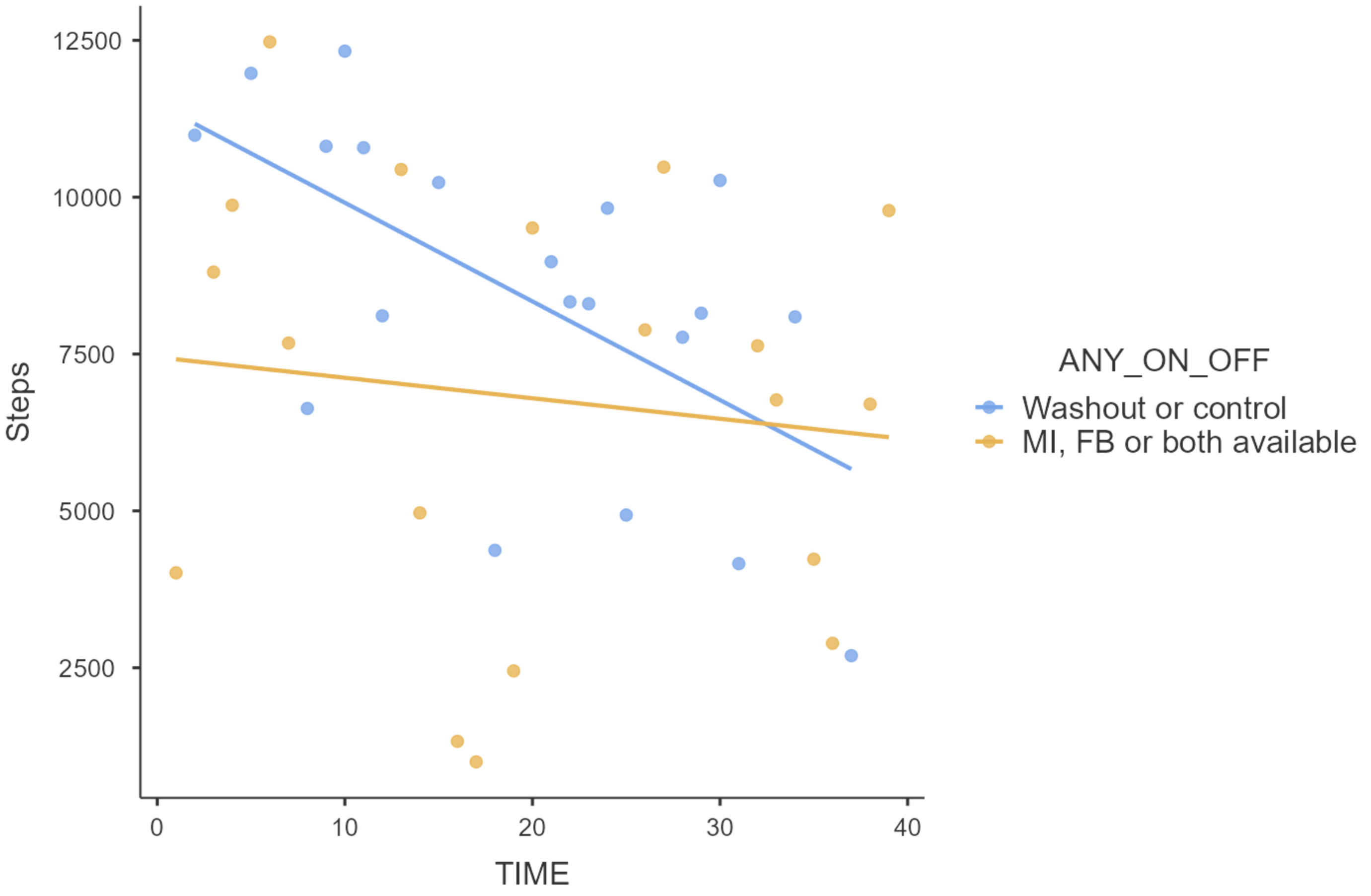

Participant 15

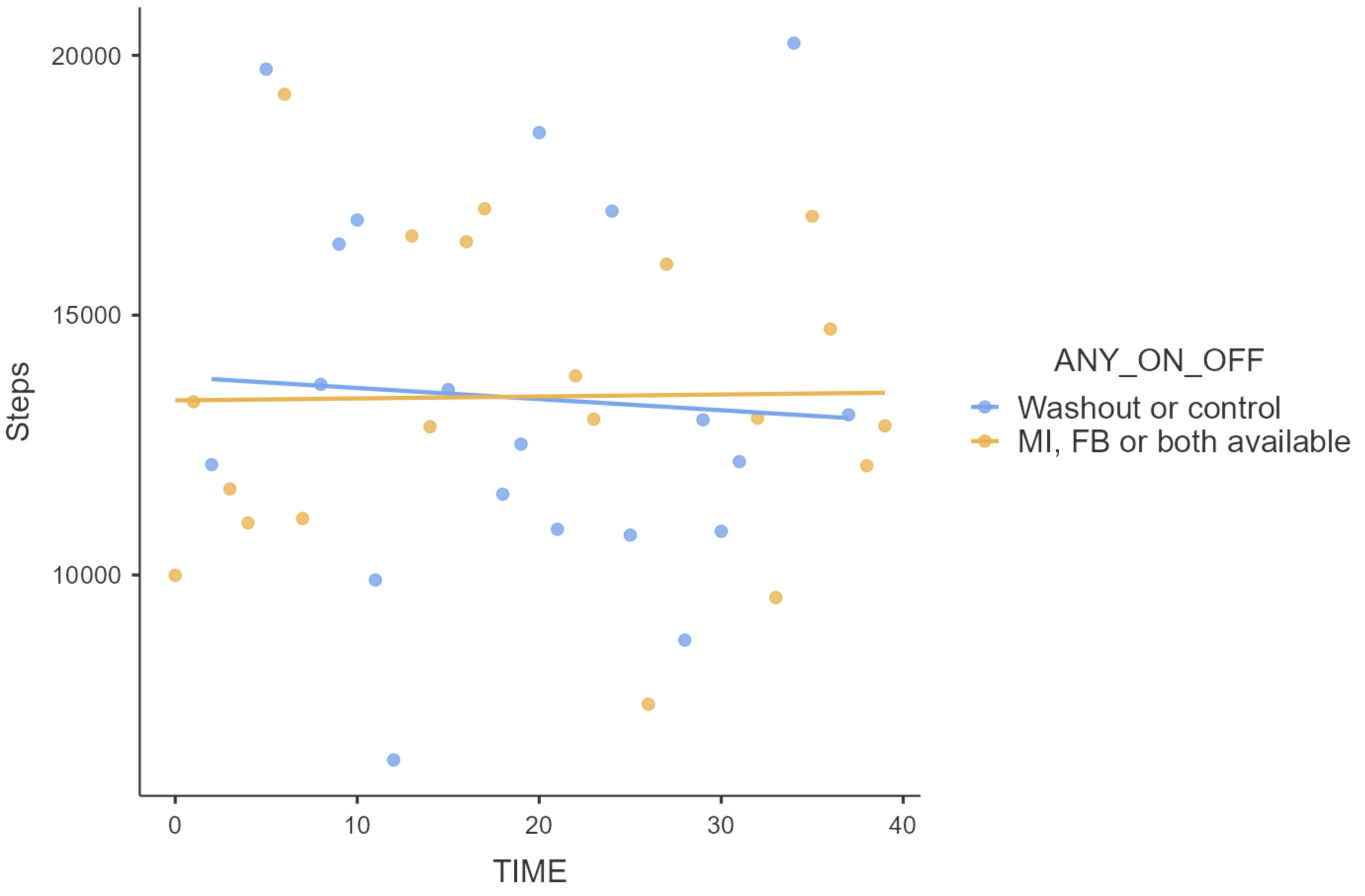

Supplement: Multimedia Appendix 4 [file formative_v7i1e34232_app4.pdf]
