# Supplementary material for: Biofeedback and Digitalized Motivational Interviewing to Increase Daily Physical Activity: Series of Factorial N-of-1 Randomized Controlled Trials Piloting the Precious App
Source: JMIR Form Res. 2023 Nov 23;7:e34232. doi: 10.2196/34232 (PMC10704305; doi:10.2196/34232)
Supplement: Multimedia Appendix 5 [file formative_v7i1e34232_app5.pdf]

CONSORT-EHEALTH (V 1.6.1) - Submission/Publication Form

The CONSORT-EHEALTH checklist is intended for authors of randomized trials evaluating web-based and Internet-based applications/interventions, including mobile interventions, electronic games (incl multiplayer games), social media, certain telehealth applications, and other interactive and/or networked electronic applications. Some of the items (e.g. all subitems under item 5 - description of the intervention) may also be applicable for other study designs.

The goal of the CONSORT EHEALTH checklist and guideline is to be
a) a guide for reporting for authors of RCTs,
b) to form a basis for appraisal of an ehealth trial (in terms of validity)

CONSORT-EHEALTH items/subitems are MANDATORY reporting items for studies published in the Journal of Medical Internet Research and other journals / scientific societies endorsing the checklist.

Items numbered 1., 2., 3., 4a., 4b etc are original CONSORT or CONSORT-NPT (non-pharmacologic treatment) items.
Items with Roman numerals (i., ii, iii, iv etc.) are CONSORT-EHEALTH extensions/clarifications.

As the CONSORT-EHEALTH checklist is still considered in a formative stage, we would ask that you also RATE ON A SCALE OF 1-5 how important/useful you feel each item is FOR THE PURPOSE OF THE CHECKLIST and reporting guideline (optional).

Mandatory reporting items are marked with a red *.
In the textboxes, either copy & paste the relevant sections from your manuscript into this form - please include any quotes from your manuscript in QUOTATION MARKS,
or answer directly by providing additional information not in the manuscript, or elaborating on why the item was not relevant for this study.

YOUR ANSWERS WILL BE PUBLISHED AS A SUPPLEMENTARY FILE TO YOUR PUBLICATION IN JMIR AND ARE CONSIDERED PART OF YOUR PUBLICATION (IF ACCEPTED).
Please fill in these questions diligently. Information will not be copyedited, so please use proper spelling and grammar, use correct capitalization, and avoid abbreviations.

DO NOT FORGET TO SAVE AS PDF _AND_ CLICK THE SUBMIT BUTTON SO YOUR ANSWERS ARE IN OUR DATABASE !!!

Citation Suggestion (if you append the pdf as Appendix we suggest to cite this paper in the caption):
Eysenbach G, CONSORT-EHEALTH Group
CONSORT-EHEALTH: Improving and Standardizing Evaluation Reports of Web-based and Mobile Health Interventions
J Med Internet Res 2011;13(4):e126

URL: http://www.jmir.org/2011/4/e126/
doi: 10.2196/jmir.1923
PMID: 22209829

transcender@gmail.com Switch accounts

Not shared

Draft saved

* Indicates required question

Your name *

First Last

Keegan Knittle

Primary Affiliation (short), City, Country *

University of Toronto, Toronto, Canada

University of Jyväskylä, Finland

Your e-mail address *

abc@gmail.com

keegan.p.knittle@jyu.fi

Title of your manuscript *

Provide the (draft) title of your manuscript.

Biofeedback and Digitalized Motivational Interviewing to Increase Daily Physical Activity: Series of Factorial N-of-1 Randomized Controlled Trials Piloting the Precious App

Name of your App/Software/Intervention *

If there is a short and a long/alternate name, write the short name first and add the long name in brackets.

PRECIOUS (PREventive Care Infrastructure bas

Evaluated Version (if any)

e.g. "V1", "Release 2017-03-01", "Version 2.0.27913"

not applicable

Language(s) *

What language is the intervention/app in? If multiple languages are available, separate by comma (e.g. "English, French")

English

URL of your Intervention Website or App

e.g. a direct link to the mobile app on app in appstore (itunes, Google Play), or URL of the website. If the intervention is a DVD or hardware, you can also link to an Amazon page.

http://www.thepreciousproject.eu/

URL of an image/screenshot (optional)

Your answer

Accessibility *

Can an enduser access the intervention presently?

- ( ) access is free and open
- ( ) access only for special usergroups, not open
- ( ) access is open to everyone, but requires payment/subscription/in-app purchases
- (•) app/intervention no longer accessible
- ( ) Other:

Primary Medical Indication/Disease/Condition *

e.g. "Stress", "Diabetes", or define the target group in brackets after the condition, e.g. "Autism (Parents of children with)", "Alzheimers (Informal Caregivers of)"

Physical activity promotion

Primary Outcomes measured in trial *

comma-separated list of primary outcomes reported in the trial

Daily steps

Secondary/other outcomes

Are there any other outcomes the intervention is expected to affect?

Your answer

Recommended "Dose" *

What do the instructions for users say on how often the app should be used?

- ( ) Approximately Daily
- ( ) Approximately Weekly
- ( ) Approximately Monthly
- ( ) Approximately Yearly
- (x) "as needed"
- ( ) Other:

Approx. Percentage of Users (starters) still using the app as recommended after 3 months *

- (x) unknown / not evaluated
- ( ) 0-10%
- ( ) 11-20%
- ( ) 21-30%
- ( ) 31-40%
- ( ) 41-50%
- ( ) 51-60%
- ( ) 61-70%
- ( ) 71%-80%
- ( ) 81-90%
- ( ) 91-100%
- ( ) Other:

Overall, was the app/intervention effective? *

- ( ) yes: all primary outcomes were significantly better in intervention group vs control
- ( ) partly: SOME primary outcomes were significantly better in intervention group vs control
- (•) no statistically significant difference between control and intervention
- ( ) potentially harmful: control was significantly better than intervention in one or more outcomes
- ( ) inconclusive: more research is needed
- ( ) Other:

Article Preparation Status/Stage *

At which stage in your article preparation are you currently (at the time you fill in this form)

- ( ) not submitted yet - in early draft status
- ( ) not submitted yet - in late draft status, just before submission
- ( ) submitted to a journal but not reviewed yet
- ( ) submitted to a journal and after receiving initial reviewer comments
- (•) submitted to a journal and accepted, but not published yet
- ( ) published
- ( ) Other:

Journal *

If you already know where you will submit this paper (or if it is already submitted), please provide the journal name (if it is not JMIR, provide the journal name under "other")

- ( ) not submitted yet / unclear where I will submit this
- ( ) Journal of Medical Internet Research (JMIR)
- ( ) JMIR mHealth and UHealth
- ( ) JMIR Serious Games
- ( ) JMIR Mental Health
- ( ) JMIR Public Health
- (x) JMIR Formative Research
- ( ) Other JMIR sister journal
- ( ) Other:

Is this a full powered effectiveness trial or a pilot/feasibility trial? *

- (x) Pilot/feasibility
- ( ) Fully powered

Manuscript tracking number *

If this is a JMIR submission, please provide the manuscript tracking number under "other" (The ms tracking number can be found in the submission acknowledgement email, or when you login as author in JMIR. If the paper is already published in JMIR, then the ms tracking number is the four-digit number at the end of the DOI, to be found at the bottom of each published article in JMIR)

- ( ) no ms number (yet) / not (yet) submitted to / published in JMIR
- (x) Other: #34232

TITLE AND ABSTRACT

1a) TITLE: Identification as a randomized trial in the title

1a) Does your paper address CONSORT item 1a? *

I.e does the title contain the phrase "Randomized Controlled Trial"? (if not, explain the reason under "other")

- (●) yes
- ( ) Other:

1a-i) Identify the mode of delivery in the title

Identify the mode of delivery. Preferably use “web-based” and/or “mobile” and/or “electronic game” in the title. Avoid ambiguous terms like “online”, “virtual”, “interactive”. Use “Internet-based” only if Intervention includes non-web-based Internet components (e.g. email), use “computer-based” or “electronic” only if offline products are used. Use “virtual” only in the context of “virtual reality” (3-D worlds). Use “online” only in the context of “online support groups”. Complement or substitute product names with broader terms for the class of products (such as “mobile” or “smart phone” instead of “iphone”), especially if the application runs on different platforms.

	1	2	3	4	5	
subitem not at all important	○	○	○	○	○	essential

Does your paper address subitem 1a-i? *

Copy and paste relevant sections from manuscript title (include quotes in quotation marks "like this" to indicate direct quotes from your manuscript), or elaborate on this item by providing additional information not in the ms, or briefly explain why the item is not applicable/relevant for your study

"The Precious smartphone app was developed to investigate the effect of specific, modular intervention elements on physical activity, and to examine theory-based predictors within individuals."

1a-ii) Non-web-based components or important co-interventions in title

Mention non-web-based components or important co-interventions in title, if any (e.g., “with telephone support”).

	1	2	3	4	5	
subitem not at all important	○	○	○	○	○	essential

Does your paper address subitem 1a-ii?

Copy and paste relevant sections from manuscript title (include quotes in quotation marks "like this" to indicate direct quotes from your manuscript), or elaborate on this item by providing additional information not in the ms, or briefly explain why the item is not applicable/relevant for your study

not applicable as all content app based

1a-iii) Primary condition or target group in the title

Mention primary condition or target group in the title, if any (e.g., “for children with Type I Diabetes”) Example: A Web-based and Mobile Intervention with Telephone Support for Children with Type I Diabetes: Randomized Controlled Trial

	1	2	3	4	5	
subitem not at all important	○	○	○	○	○	essential

Does your paper address subitem 1a-iii? *

Copy and paste relevant sections from manuscript title (include quotes in quotation marks "like this" to indicate direct quotes from your manuscript), or elaborate on this item by providing additional information not in the ms, or briefly explain why the item is not applicable/relevant for your study

not applicable as not for a specific population

1b) ABSTRACT: Structured summary of trial design, methods, results, and conclusions

NPT extension: Description of experimental treatment, comparator, care providers, centers, and blinding status.

1b-i) Key features/functionalities/components of the intervention and comparator in the METHODS section of the ABSTRACT

Mention key features/functionalities/components of the intervention and comparator in the abstract. If possible, also mention theories and principles used for designing the site. Keep in mind the needs of systematic reviewers and indexers by including important synonyms. (Note: Only report in the abstract what the main paper is reporting. If this information is missing from the main body of text, consider adding it)

	1	2	3	4	5	
subitem not at all important	○	○	○	○	◉	essential

Clear selection

Does your paper address subitem 1b-i? *

Copy and paste relevant sections from the manuscript abstract (include quotes in quotation marks "like this" to indicate direct quotes from your manuscript), or elaborate on this item by providing additional information not in the ms, or briefly explain why the item is not applicable/relevant for your study

Yes. "The factorial design included seven 2-day biofeedback interventions with a Firstbeat Bodyguard 2 (Firstbeat Technologies Ltd) heart- rate variability sensor, and seven 2-day dMI interventions, a wash-out- day after each intervention, and 11 control days."

1b-ii) Level of human involvement in the METHODS section of the ABSTRACT

Clarify the level of human involvement in the abstract, e.g., use phrases like “fully automated” vs. “therapist/nurse/care provider/physician-assisted” (mention number and expertise of providers involved, if any). (Note: Only report in the abstract what the main paper is reporting. If this information is missing from the main body of text, consider adding it)

	1	2	3	4	5	
subitem not at all important	○	○	○	○	○	essential

Does your paper address subitem 1b-ii?

Copy and paste relevant sections from the manuscript abstract (include quotes in quotation marks "like this" to indicate direct quotes from your manuscript), or elaborate on this item by providing additional information not in the ms, or briefly explain why the item is not applicable/relevant for your study

Yes. "This study pilot- tested a fully automated factorial N-of-1 randomized controlled trial (RCT) with the Precious app"

1b-iii) Open vs. closed, web-based (self-assessment) vs. face-to-face assessments in the METHODS section of the ABSTRACT

Mention how participants were recruited (online vs. offline), e.g., from an open access website or from a clinic or a closed online user group (closed usergroup trial), and clarify if this was a purely web-based trial, or there were face-to-face components (as part of the intervention or for assessment). Clearly say if outcomes were self-assessed through questionnaires (as common in web-based trials). Note: In traditional offline trials, an open trial (open-label trial) is a type of clinical trial in which both the researchers and participants know which treatment is being administered. To avoid confusion, use “blinded” or “unblinded” to indicated the level of blinding instead of “open”, as “open” in web-based trials usually refers to “open access” (i.e. participants can self-enrol). (Note: Only report in the abstract what the main paper is reporting. If this information is missing from the main body of text, consider adding it)

	1	2	3	4	5	
subitem not at all important	○	○	○	○	○	essential

Does your paper address subitem 1b-iii?

Copy and paste relevant sections from the manuscript abstract (include quotes in quotation marks "like this" to indicate direct quotes from your manuscript), or elaborate on this item by providing additional information not in the ms, or briefly explain why the item is not applicable/relevant for your study

In total, 15 adults recruited from newspaper advertisements participated in a 40-day factorial N-of-1 RCT. They installed 2 study apps onto their phones: one to receive intervention elements on individually randomized days and one to collect ecological momentary assessment (EMA) data on self-efficacy, motivation, perceived barriers, pain, and illness.

1b-iv) RESULTS section in abstract must contain use data

Report number of participants enrolled/assessed in each group, the use/uptake of the intervention (e.g., attrition/adherence metrics, use over time, number of logins etc.), in addition to primary/secondary outcomes. (Note: Only report in the abstract what the main paper is reporting. If this information is missing from the main body of text, consider adding it)

	1	2	3	4	5	
subitem not at all important	○	○	○	○	○	essential

Does your paper address subitem 1b-iv?

Copy and paste relevant sections from the manuscript abstract (include quotes in quotation marks "like this" to indicate direct quotes from your manuscript), or elaborate on this item by providing additional information not in the ms, or briefly explain why the item is not applicable/relevant for your study

"The implementation of the factorial design was successful, with the dMI features used, on average, 5.1 times of the 7 available interventions (SD=1.0). Biofeedback interventions were used, on average, 5.7 times out of 7 (SD=1.4), although 3 participants used this feature a day later than suggested and 1 did not use it at all. "

1b-v) CONCLUSIONS/DISCUSSION in abstract for negative trials

Conclusions/Discussions in abstract for negative trials: Discuss the primary outcome - if the trial is negative (primary outcome not changed), and the intervention was not used, discuss whether negative results are attributable to lack of uptake and discuss reasons. (Note: Only report in the abstract what the main paper is reporting. If this information is missing from the main body of text, consider adding it)

	1	2	3	4	5	
subitem not at all important	(•)	( )	( )	( )	( )	essential

Clear selection

Does your paper address subitem 1b-v?

Copy and paste relevant sections from the manuscript abstract (include quotes in quotation marks "like this" to indicate direct quotes from your manuscript), or elaborate on this item by providing additional information not in the ms, or briefly explain why the item is not applicable/relevant for your study

"Neither within- nor between-person analyses revealed significant intervention effects on step counts. "

INTRODUCTION

2a) In INTRODUCTION: Scientific background and explanation of rationale

2a-i) Problem and the type of system/solution

Describe the problem and the type of system/solution that is object of the study: intended as stand-alone intervention vs. incorporated in broader health care program? Intended for a particular patient population? Goals of the intervention, e.g., being more cost-effective to other interventions, replace or complement other solutions? (Note: Details about the intervention are provided in “Methods” under 5)

	1	2	3	4	5	
subitem not at all important	( )	( )	( )	( )	( )	essential

Does your paper address subitem 2a-i? *

Copy and paste relevant sections from the manuscript (include quotes in quotation marks "like this" to indicate direct quotes from your manuscript), or elaborate on this item by providing additional information not in the ms, or briefly explain why the item is not applicable/relevant for your study

"Most adults do not engage in sufficient physical activity for good health [1]. " and "The Precious mobile app was designed building on theory and evidence to increase users' physical activity using motivational and self-regulatory techniques [37]. The core functions of the app were as a tracking tool with self-regulatory BCTs, such as behavioral goal setting and self-monitoring, and motivational tools aimed at increasing uptake of the tracking features."

2a-ii) Scientific background, rationale: What is known about the (type of) system

Scientific background, rationale: What is known about the (type of) system that is the object of the study (be sure to discuss the use of similar systems for other conditions/diagnoses, if appropiate), motivation for the study, i.e. what are the reasons for and what is the context for this specific study, from which stakeholder viewpoint is the study performed, potential impact of findings [2]. Briefly justify the choice of the comparator.

	1	2	3	4	5	
subitem not at all important	○	○	○	○	○	essential

Does your paper address subitem 2a-ii? *

Copy and paste relevant sections from the manuscript (include quotes in quotation marks "like this" to indicate direct quotes from your manuscript), or elaborate on this item by providing additional information not in the ms, or briefly explain why the item is not applicable/relevant for your study

"In a 3-month usability RCT, the Precious app was found to be acceptable among persons with obesity, and participants were particularly satisfied with the app's biofeedback report and physical activity modules [44]."

2b) In INTRODUCTION: Specific objectives or hypotheses

Does your paper address CONSORT subitem 2b? *

Copy and paste relevant sections from the manuscript (include quotes in quotation marks "like this" to indicate direct quotes from your manuscript), or elaborate on this item by providing additional information not in the ms, or briefly explain why the item is not applicable/relevant for your study

"The analyses addressed the following research questions (RQs): (1) Do people take more steps on days when they are offered motivational smartphone-based interventions (intervention 1: MI components; intervention 2: biofeedback) compared with nonintervention days? (RQ 1) and (2) Do daily self-efficacy and motivation predict daily steps in individuals? (RQ 2)."

METHODS

3a) Description of trial design (such as parallel, factorial) including allocation ratio

Does your paper address CONSORT subitem 3a? *

Copy and paste relevant sections from the manuscript (include quotes in quotation marks "like this" to indicate direct quotes from your manuscript), or elaborate on this item by providing additional information not in the ms, or briefly explain why the item is not applicable/relevant for your study

"This study was a 40-day 2 × 2 factorial N-of-1 RCT testing the effects of dMI and biofeedback and involved twice-daily (morning and afternoon) ecological momentary assessments (EMAs) of psychological and environmental predictors of physical activity" and "The 40-day trial included 12 three-day active study periods (ie, 2 days during which one of the study conditions described in the 'Randomization' section was implemented plus a wash-out day in which dMI features were hidden from the app and biofeedback notifications ceased), 2 additional control days (one each after the fourth and eighth active study periods), and a 2-day lead-out period in which all app features were available "

3b) Important changes to methods after trial commencement (such as eligibility criteria), with reasons

Does your paper address CONSORT subitem 3b? *

Copy and paste relevant sections from the manuscript (include quotes in quotation marks "like this" to indicate direct quotes from your manuscript), or elaborate on this item by providing additional information not in the ms, or briefly explain why the item is not applicable/relevant for your study

no changes to methods after trial commencement.

3b-i) Bug fixes, Downtimes, Content Changes

Bug fixes, Downtimes, Content Changes: ehealth systems are often dynamic systems. A description of changes to methods therefore also includes important changes made on the intervention or comparator during the trial (e.g., major bug fixes or changes in the functionality or content) (5-iii) and other "unexpected events" that may have influenced study design such as staff changes, system failures/downtimes, etc. [2].

	1	2	3	4	5	
subitem not at all important	○	○	○	○	○	essential

Does your paper address subitem 3b-i?

Copy and paste relevant sections from the manuscript (include quotes in quotation marks "like this" to indicate direct quotes from your manuscript), or elaborate on this item by providing additional information not in the ms, or briefly explain why the item is not applicable/relevant for your study

no bug fixes or downtimes or content changes during the study.

4a) Eligibility criteria for participants

Does your paper address CONSORT subitem 4a? *

Copy and paste relevant sections from the manuscript (include quotes in quotation marks "like this" to indicate direct quotes from your manuscript), or elaborate on this item by providing additional information not in the ms, or briefly explain why the item is not applicable/relevant for your study

"eligibility criteria: age of >18 years, ability to speak Finnish, ability to read and understand English, no contraindications to engaging in physical activity as assessed using the Physical Activity Readiness Questionnaire [65], ownership of a smartphone with a compatible operating system (“Jelly Bean” version 4.1 or higher for Android), willingness to install the Precious app on said smartphone for a 40-day period, no use of any physical activity trackers (eg, Fitbit, Garmin, or Misfit) or physical activity apps in the previous 6 months, no participation in other trials or behavior change programs in the previous 6 months or during the trial, levels of physical activity below the recommendation of 150 minutes per week of moderate-intensity physical activity [66], and willingness to wear an activity tracker for the duration of the study. In addition, participants were excluded if they were seeking to be enrolled in the trial concurrently with a friend or relative. This exclusion criterion was applied to avoid exposure to randomly timed intervention materials during control days through the other person’s phone."

4a-i) Computer / Internet literacy

Computer / Internet literacy is often an implicit “de facto” eligibility criterion - this should be explicitly clarified.

	1	2	3	4	5	
subitem not at all important	○	○	○	○	○	essential

Does your paper address subitem 4a-i?

Copy and paste relevant sections from the manuscript (include quotes in quotation marks "like this" to indicate direct quotes from your manuscript), or elaborate on this item by providing additional information not in the ms, or briefly explain why the item is not applicable/relevant for your study

"ownership and use of a smartphone with a compatible operating system (“Jelly Bean” version 4.1 or higher for Android), "

4a-ii) Open vs. closed, web-based vs. face-to-face assessments:

Open vs. closed, web-based vs. face-to-face assessments: Mention how participants were recruited (online vs. offline), e.g., from an open access website or from a clinic, and clarify if this was a purely web-based trial, or there were face-to-face components (as part of the intervention or for assessment), i.e., to what degree got the study team to know the participant. In online-only trials, clarify if participants were quasi-anonymous and whether having multiple identities was possible or whether technical or logistical measures (e.g., cookies, email confirmation, phone calls) were used to detect/prevent these.

	1	2	3	4	5	
subitem not at all important	○	○	○	○	○	essential

Does your paper address subitem 4a-ii? *

Copy and paste relevant sections from the manuscript (include quotes in quotation marks "like this" to indicate direct quotes from your manuscript), or elaborate on this item by providing additional information not in the ms, or briefly explain why the item is not applicable/relevant for your study

"Participants were recruited from the general population using advertisements in the “Metro” newspaper of the Helsinki area, Finland, and a Facebook page and targeted advertisements in October 2016. People who responded to the advertisement were contacted by the research team via email or phone to establish whether they met the following eligibility criteria:"

4a-iii) Information giving during recruitment

Information given during recruitment. Specify how participants were briefed for recruitment and in the informed consent procedures (e.g., publish the informed consent documentation as appendix, see also item X26), as this information may have an effect on user self-selection, user expectation and may also bias results.

	1	2	3	4	5	
subitem not at all important	○	○	○	○	○	essential

Does your paper address subitem 4a-iii?

Copy and paste relevant sections from the manuscript (include quotes in quotation marks "like this" to indicate direct quotes from your manuscript), or elaborate on this item by providing additional information not in the ms, or briefly explain why the item is not applicable/relevant for your study

From informed consent document: "The study period is approximately forty days, during which we hope you will use the PRECIOUS application on your own phone and keep the related activity tracker/activity monitor with you at all times. The daily use of the PRECIOUS application takes about 2-10 minutes, depending on how much you use the optional features or tasks. At several points in the trial, you will have the chance to complete a FirstBeat well-being analysis using a sensor that attaches to your chest with a sticker and measures heart rate variability. You will receive the results of the analyses on your phone (a value of approximately 150 euros/analysis). You can participate in the study without the FirstBeat well-being analysis if you choose."

4b) Settings and locations where the data were collected

Does your paper address CONSORT subitem 4b? *

Copy and paste relevant sections from the manuscript (include quotes in quotation marks "like this" to indicate direct quotes from your manuscript), or elaborate on this item by providing additional information not in the ms, or briefly explain why the item is not applicable/relevant for your study

"To begin the study, participants had a 60- to 90-minute long individual instruction session with a researcher (JN) at a university office. " and "Participants were recruited from the ... Helsinki area, Finland"

4b-i) Report if outcomes were (self-)assessed through online questionnaires

Clearly report if outcomes were (self-)assessed through online questionnaires (as common in web-based trials) or otherwise.

	1	2	3	4	5	
subitem not at all important	○	○	○	○	○	essential

Does your paper address subitem 4b-i? *

Copy and paste relevant sections from the manuscript (include quotes in quotation marks "like this" to indicate direct quotes from your manuscript), or elaborate on this item by providing additional information not in the ms, or briefly explain why the item is not applicable/relevant for your study

Yes. "Physical Activity Data Collection
The primary outcome was daily steps, assessed continuously throughout the study using a waterproof Xiaomi Mi Band wrist-worn accelerometer. Participants agreed to wear the Mi Band for the entire duration of the 40-day trial and to only remove it temporarily. Mi Band had a step count accuracy of 96.6%, which placed it among the most accurate commercially available, Bluetooth-enabled wrist-worn step counters at the outset of this trial [63]. Time-stamped step counts were passed from the Mi Band to the Precious app via Bluetooth every 10 minutes.
Daily EMA
Participants were invited to report their self-efficacy every morning between 9 AM and 10 AM with the following question—“At this moment, how confident are you that you can be physically active for at least 30 min today?”—on a 9-step visual analog scale from Not at all to Extremely.
Participants were invited to report their motivation twice each day (once between 9 AM and 10 AM and once between 4 PM and 5 PM) with the following question—“At this moment, how motivated are you to be physically active”—on a 9-step visual analog scale from Not at all to Extremely.
Participants were invited to report their perceived barriers to being active on that day each day between 9 AM and 10 AM to control for external factors that were not captured by our predictors with the following question—“To what extent are other things you need to do today stopping you from being physically active?”—on a 9-step scale from Not at all to Extremely.
Participants were invited to report their pain or illness every day between 4 PM and 5 PM with the following question—“Did illness or pain stop you from being physically active today?”—and 3 options: none, somewhat, and seriously.
The EMA questions were based on previous research [64]. To minimize measurement reactivity and separate these daily assessments from the motivational interventions of the Precious app, the questions were delivered to participants’ phones via a specific EMA measurement app developed by the Netherlands Organisation for Applied Scientific Research.
Intervention use was conceptualized as (1) participants accessing a specific MI feature on the day the feature was available and (2) participants uploading the biofeedback report to the server. Both actions left a time stamp on the server file.
"

4b-ii) Report how institutional affiliations are displayed

Report how institutional affiliations are displayed to potential participants [on ehealth media], as affiliations with prestigious hospitals or universities may affect volunteer rates, use, and reactions with regards to an intervention.(Not a required item – describe only if this may bias results)

	1	2	3	4	5	
subitem not at all important	○	○	○	○	○	essential

Does your paper address subitem 4b-ii?

Copy and paste relevant sections from the manuscript (include quotes in quotation marks "like this" to indicate direct quotes from your manuscript), or elaborate on this item by providing additional information not in the ms, or briefly explain why the item is not applicable/relevant for your study

this is not applicable to our study

5) The interventions for each group with sufficient details to allow replication, including how and when they were actually administered

5-i) Mention names, credential, affiliations of the developers, sponsors, and owners

Mention names, credential, affiliations of the developers, sponsors, and owners [6] (if authors/evaluators are owners or developer of the software, this needs to be declared in a “Conflict of interest” section or mentioned elsewhere in the manuscript).

	1	2	3	4	5	
subitem not at all important	○	○	○	○	○	essential

Does your paper address subitem 5-i?

Copy and paste relevant sections from the manuscript (include quotes in quotation marks "like this" to indicate direct quotes from your manuscript), or elaborate on this item by providing additional information not in the ms, or briefly explain why the item is not applicable/relevant for your study

"Interventions
On MI intervention days, participants received a morning notification of new content: “A new test period has started. Come see what the Precious app has to offer.” The notification remained visible until it was touched to open the app or swiped away. The digitalized elements of MI (Figure 2) are described in detail in the study by Nurmi et al [37] and include the techniques listed in Table S1 in Multimedia Appendix 1. To imitate the interactivity of face-to-face MI, dMI tools were offered in a stepwise manner: 3 dMI tools in 3 consecutive periods. Set A included “What do I want” and “Choose Favorite PAs,” set B included “Importance Ruler” and “What’s Next (stage of change),” and set C included “Time Machine” and “Confidence Ruler.” Thus, the tools offered slight variability over the course of the trial to maintain user interest.
The biofeedback interventions started with a preparation notification the day before each measurement: “Tomorrow is a Firstbeat assessment day. Be sure to have the device charged and ready to wear tomorrow.” On the first intervention day, participants received a notification stating the following: “Today is the day! Please wear your Firstbeat device today and upload the data tomorrow.” On the second intervention morning, the notification read the following: “We hope you slept well. Please upload the data from your Firstbeat device to get a report on your activity, sleep, and stress levels.” To access the report, participants needed to plug the wearable sensor into their computer’s USB port and upload the data to the trial website. The biofeedback report was provided using the Firstbeat Bodyguard 2 heart rate variability monitor (Firstbeat Technologies Ltd). These data were first pushed securely to the Firstbeat company servers for analysis and then passed securely to the Precious server for storage. This intervention used BCTs 2.4 (self-monitoring outcomes), 2.6 (biofeedback), 2.7 (feedback on outcomes of behavior), 7.1 (prompts/cues), and 12.5 (adding objects to the environment) [12]."

5-ii) Describe the history/development process

Describe the history/development process of the application and previous formative evaluations (e.g., focus groups, usability testing), as these will have an impact on adoption/use rates and help with interpreting results.

	1	2	3	4	5	
subitem not at all important	○	○	○	○	○	essential

Does your paper address subitem 5-ii?

Copy and paste relevant sections from the manuscript (include quotes in quotation marks "like this" to indicate direct quotes from your manuscript), or elaborate on this item by providing additional information not in the ms, or briefly explain why the item is not applicable/relevant for your study

Detailed descriptions of the development process are available from: https://mhealth.jmir.org/2020/1/e12884

5-iii) Revisions and updating

Revisions and updating. Clearly mention the date and/or version number of the application/intervention (and comparator, if applicable) evaluated, or describe whether the intervention underwent major changes during the evaluation process, or whether the development and/or content was “frozen” during the trial. Describe dynamic components such as news feeds or changing content which may have an impact on the replicability of the intervention (for unexpected events see item 3b).

	1	2	3	4	5	
subitem not at all important	○	○	○	○	○	essential

Does your paper address subitem 5-iii?

Copy and paste relevant sections from the manuscript (include quotes in quotation marks "like this" to indicate direct quotes from your manuscript), or elaborate on this item by providing additional information not in the ms, or briefly explain why the item is not applicable/relevant for your study

"Always Available App Features
On control and wash-out days, the dMI and biofeedback tools were hidden from the app. The participants could still freely access all the self-regulatory BCTs, including behavioral goal setting and self-monitoring (the full list is available in Table S1 in Multimedia Appendix 1). They also received communication related to their goal progress at 5 PM every day: “You’ve taken [step total] steps today—that’s (100*[step total]/[goal amount])% of your goal. Keep going!” (BCT 2.2, feedback on behavior and goal progress [12]). If participants set a step goal and reached it, they received a congratulatory message: “Good job! You’ve achieved your step goal for today. Click here to see your progress.” (BCT 10.4, social reward [12])."

5-iv) Quality assurance methods

Provide information on quality assurance methods to ensure accuracy and quality of information provided [1], if applicable.

	1	2	3	4	5	
subitem not at all important	○	○	○	○	○	essential

Does your paper address subitem 5-iv?

Copy and paste relevant sections from the manuscript (include quotes in quotation marks "like this" to indicate direct quotes from your manuscript), or elaborate on this item by providing additional information not in the ms, or briefly explain why the item is not applicable/relevant for your study

Not relevant here as no changes took place in app content during the trial.

5-v) Ensure replicability by publishing the source code, and/or providing screenshots/screen-capture video, and/or providing flowcharts of the algorithms used

Ensure replicability by publishing the source code, and/or providing screenshots/screen-capture video, and/or providing flowcharts of the algorithms used. Replicability (i.e., other researchers should in principle be able to replicate the study) is a hallmark of scientific reporting.

	1	2	3	4	5	
subitem not at all important	○	○	○	○	○	essential

Does your paper address subitem 5-v?

Copy and paste relevant sections from the manuscript (include quotes in quotation marks "like this" to indicate direct quotes from your manuscript), or elaborate on this item by providing additional information not in the ms, or briefly explain why the item is not applicable/relevant for your study

Detailed descriptions of intervention content are available from: https://mhealth.jmir.org/2020/1/e12884

5-vi) Digital preservation

Digital preservation: Provide the URL of the application, but as the intervention is likely to change or disappear over the course of the years; also make sure the intervention is archived (Internet Archive, webcitation.org, and/or publishing the source code or screenshots/videos alongside the article). As pages behind login screens cannot be archived, consider creating demo pages which are accessible without login.

	1	2	3	4	5	
subitem not at all important	○	○	○	○	○	essential

Does your paper address subitem 5-vi?

Copy and paste relevant sections from the manuscript (include quotes in quotation marks "like this" to indicate direct quotes from your manuscript), or elaborate on this item by providing additional information not in the ms, or briefly explain why the item is not applicable/relevant for your study

Source files of the app are available upon request.

5-vii) Access

Access: Describe how participants accessed the application, in what setting/context, if they had to pay (or were paid) or not, whether they had to be a member of specific group. If known, describe how participants obtained “access to the platform and Internet” [1]. To ensure access for editors/reviewers/readers, consider to provide a “backdoor” login account or demo mode for reviewers/readers to explore the application (also important for archiving purposes, see vi).

	1	2	3	4	5	
subitem not at all important	○	○	○	○	○	essential

Does your paper address subitem 5-vii? *

Copy and paste relevant sections from the manuscript (include quotes in quotation marks "like this" to indicate direct quotes from your manuscript), or elaborate on this item by providing additional information not in the ms, or briefly explain why the item is not applicable/relevant for your study

Participants only had access to the app for the trial. Not released to public.

5-viii) Mode of delivery, features/functionalities/components of the intervention and comparator, and the theoretical framework

Describe mode of delivery, features/functionalities/components of the intervention and comparator, and the theoretical framework [6] used to design them (instructional strategy [1], behaviour change techniques, persuasive features, etc., see e.g., [7, 8] for terminology). This includes an in-depth description of the content (including where it is coming from and who developed it) [1]," whether [and how] it is tailored to individual circumstances and allows users to track their progress and receive feedback" [6]. This also includes a description of communication delivery channels and – if computer-mediated communication is a component – whether communication was synchronous or asynchronous [6]. It also includes information on presentation strategies [1], including page design principles, average amount of text on pages, presence of hyperlinks to other resources, etc. [1].

	1	2	3	4	5	
subitem not at all important	○	○	○	○	○	essential

Does your paper address subitem 5-viii? *

Copy and paste relevant sections from the manuscript (include quotes in quotation marks "like this" to indicate direct quotes from your manuscript), or elaborate on this item by providing additional information not in the ms, or briefly explain why the item is not applicable/relevant for your study

Detailed descriptions of intervention content are available from: https://mhealth.jmir.org/2020/1/e12884

5-ix) Describe use parameters

Describe use parameters (e.g., intended “doses” and optimal timing for use). Clarify what instructions or recommendations were given to the user, e.g., regarding timing, frequency, heaviness of use, if any, or was the intervention used ad libitum.

	1	2	3	4	5	
subitem not at all important	○	○	○	○	○	essential

Does your paper address subitem 5-ix?

Copy and paste relevant sections from the manuscript (include quotes in quotation marks "like this" to indicate direct quotes from your manuscript), or elaborate on this item by providing additional information not in the ms, or briefly explain why the item is not applicable/relevant for your study

Your answer

5-x) Clarify the level of human involvement

Clarify the level of human involvement (care providers or health professionals, also technical assistance) in the e-intervention or as co-intervention (detail number and expertise of professionals involved, if any, as well as “type of assistance offered, the timing and frequency of the support, how it is initiated, and the medium by which the assistance is delivered”. It may be necessary to distinguish between the level of human involvement required for the trial, and the level of human involvement required for a routine application outside of a RCT setting (discuss under item 21 – generalizability).

	1	2	3	4	5	
subitem not at all important	○	○	○	○	○	essential

Does your paper address subitem 5-x?

Copy and paste relevant sections from the manuscript (include quotes in quotation marks "like this" to indicate direct quotes from your manuscript), or elaborate on this item by providing additional information not in the ms, or briefly explain why the item is not applicable/relevant for your study

Participants were advised to follow the instructions of the apps until their individual follow-up meetings. Printed instructions and researchers' contact details were provided with the material pack, and participants were encouraged to contact the researchers in case of any technical difficulties. All participants received a portable power bank as a gift for participating and to help keep their phones charged during the trial.

5-xi) Report any prompts/reminders used

Report any prompts/reminders used: Clarify if there were prompts (letters, emails, phone calls, SMS) to use the application, what triggered them, frequency etc. It may be necessary to distinguish between the level of prompts/reminders required for the trial, and the level of prompts/reminders for a routine application outside of a RCT setting (discuss under item 21 – generalizability).

	1	2	3	4	5	
subitem not at all important	○	○	○	○	○	essential

Does your paper address subitem 5-xi? *

Copy and paste relevant sections from the manuscript (include quotes in quotation marks "like this" to indicate direct quotes from your manuscript), or elaborate on this item by providing additional information not in the ms, or briefly explain why the item is not applicable/relevant for your study

On MI intervention days, participants received a morning notification of new content: “A new test period has started. Come see what the Precious app has to offer.” The notification remained visible until it was touched to open the app or swiped away. The digitalized elements of MI (Figure 2) are described in detail in the study by Nurmi et al [37] and include the techniques listed in Table S1 in Multimedia Appendix 1. To imitate the interactivity of face-to-face MI, dMI tools were offered in a stepwise manner: 3 dMI tools in 3 consecutive periods. Set A included “What do I want” and “Choose Favorite PAs,” set B included “Importance Ruler” and “What’s Next (stage of change),” and set C included “Time Machine” and “Confidence Ruler.” Thus, the tools offered slight variability over the course of the trial to maintain user interest.
The biofeedback interventions started with a preparation notification the day before each measurement: “Tomorrow is a Firstbeat assessment day. Be sure to have the device charged and ready to wear tomorrow.” On the first intervention day, participants received a notification stating the following: “Today is the day! Please wear your Firstbeat device today and upload the data tomorrow.” On the second intervention morning, the notification read the following: “We hope you slept well. Please upload the data from your Firstbeat device to get a report on your activity, sleep, and stress levels.” To access the report, participants needed to plug the wearable sensor into their computer’s USB port and upload the data to the trial website. The biofeedback report was provided using the Firstbeat Bodyguard 2 heart rate variability monitor (Firstbeat Technologies Ltd). These data were first pushed securely to the Firstbeat company servers for analysis and then passed securely to the Precious server for storage. This intervention used BCTs 2.4 (self-monitoring outcomes), 2.6 (biofeedback), 2.7 (feedback on outcomes of behavior), 7.1 (prompts/cues), and 12.5 (adding objects to the environment) [12].
Always Available App Features
On control and wash-out days, the dMI and biofeedback tools were hidden from the app. The participants could still freely access all the self-regulatory BCTs, including behavioral goal setting and self-monitoring (the full list is available in Table S1 in Multimedia Appendix 1). They also received communication related to their goal progress at 5 PM every day: “You’ve taken [step total] steps today—that’s (100*[step total]/[goal amount])% of your goal. Keep going!” (BCT 2.2, feedback on behavior and goal progress [12]). If participants set a step goal and reached it, they received a congratulatory message: “Good job! You’ve achieved your step goal for today. Click here to see your progress.” (BCT 10.4, social reward [12]).

5-xii) Describe any co-interventions (incl. training/support)

Describe any co-interventions (incl. training/support): Clearly state any interventions that are provided in addition to the targeted eHealth intervention, as ehealth intervention may not be designed as stand-alone intervention. This includes training sessions and support [1]. It may be necessary to distinguish between the level of training required for the trial, and the level of training for a routine application outside of a RCT setting (discuss under item 21 – generalizability.

	1	2	3	4	5	
subitem not at all important	○	○	○	○	○	essential

Does your paper address subitem 5-xii? *

Copy and paste relevant sections from the manuscript (include quotes in quotation marks "like this" to indicate direct quotes from your manuscript), or elaborate on this item by providing additional information not in the ms, or briefly explain why the item is not applicable/relevant for your study

On control and wash-out days, the dMI and biofeedback tools were hidden from the app. The participants could still freely access all the self-regulatory BCTs, including behavioral goal setting and self-monitoring (the full list is available in Table S1 in Multimedia Appendix 1). They also received communication related to their goal progress at 5 PM every day: “You've taken [step total] steps today—that's (100*[step total]/[goal amount])% of your goal. Keep going!” (BCT 2.2, feedback on behavior and goal progress [12]). If participants set a step goal and reached it, they received a congratulatory message: “Good job! You've achieved your step goal for today. Click here to see your progress.” (BCT 10.4, social reward [12]).

6a) Completely defined pre-specified primary and secondary outcome measures, including how and when they were assessed

Does your paper address CONSORT subitem 6a? *

Copy and paste relevant sections from the manuscript (include quotes in quotation marks "like this" to indicate direct quotes from your manuscript), or elaborate on this item by providing additional information not in the ms, or briefly explain why the item is not applicable/relevant for your study

Outcomes
Physical Activity Data Collection
The primary outcome was daily steps, assessed continuously throughout the study using a waterproof Xiaomi Mi Band wrist-worn accelerometer. Participants agreed to wear the Mi Band for the entire duration of the 40-day trial and to only remove it temporarily. Mi Band had a step count accuracy of 96.6%, which placed it among the most accurate commercially available, Bluetooth-enabled wrist-worn step counters at the outset of this trial [63]. Time-stamped step counts were passed from the Mi Band to the Precious app via Bluetooth every 10 minutes.
Daily EMA
Participants were invited to report their self-efficacy every morning between 9 AM and 10 AM with the following question—“At this moment, how confident are you that you can be physically active for at least 30 min today?”—on a 9-step visual analog scale from Not at all to Extremely.
Participants were invited to report their motivation twice each day (once between 9 AM and 10 AM and once between 4 PM and 5 PM) with the following question—“At this moment, how motivated are you to be physically active”—on a 9-step visual analog scale from Not at all to Extremely.
Participants were invited to report their perceived barriers to being active on that day each day between 9 AM and 10 AM to control for external factors that were not captured by our predictors with the following question—“To what extent are other things you need to do today stopping you from being physically active?”—on a 9-step scale from Not at all to Extremely.
Participants were invited to report their pain or illness every day between 4 PM and 5 PM with the following question—“Did illness or pain stop you from being physically active today?”—and 3 options: none, somewhat, and seriously.
The EMA questions were based on previous research [64]. To minimize measurement reactivity and separate these daily assessments from the motivational interventions of the Precious app, the questions were delivered to participants’ phones via a specific EMA measurement app developed by the Netherlands Organisation for Applied Scientific Research.
Intervention use was conceptualized as (1) participants accessing a specific MI feature on the day the feature was available and (2) participants uploading the biofeedback report to the server. Both actions left a time stamp on the server file.

6a-i) Online questionnaires: describe if they were validated for online use and apply CHERRIES items to describe how the questionnaires were designed/deployed

If outcomes were obtained through online questionnaires, describe if they were validated for online use and apply CHERRIES items to describe how the questionnaires were designed/deployed [9].

	1	2	3	4	5	
subitem not at all important	○	○	○	○	○	essential

Does your paper address subitem 6a-i?

Copy and paste relevant sections from manuscript text

Single item measures had not been validated, but had been used in an earlier n-of-1 study, showing an ability to predict behavior.

6a-ii) Describe whether and how “use” (including intensity of use/dosage) was defined/measured/monitored

Describe whether and how “use” (including intensity of use/dosage) was defined/measured/monitored (logins, logfile analysis, etc.). Use/adoption metrics are important process outcomes that should be reported in any ehealth trial.

	1	2	3	4	5	
subitem not at all important	○	○	○	○	○	essential

Does your paper address subitem 6a-ii?

Copy and paste relevant sections from manuscript text

"Intervention use was conceptualized as (1) participants accessing a specific MI feature on the day the feature was available and (2) participants uploading the biofeedback report to the server. Both actions left a time stamp on the server file."

6a-iii) Describe whether, how, and when qualitative feedback from participants was obtained

Describe whether, how, and when qualitative feedback from participants was obtained (e.g., through emails, feedback forms, interviews, focus groups).

	1	2	3	4	5	
subitem not at all important	○	○	○	○	○	essential

Does your paper address subitem 6a-iii?

Copy and paste relevant sections from manuscript text

"After the 40-day trial, participants returned to the university for an individual follow-up meeting, debriefing, and exit interview ... Participants were then asked about their experiences using the Precious app over the course of the study during a semistructured interview. "

6b) Any changes to trial outcomes after the trial commenced, with reasons

Does your paper address CONSORT subitem 6b? *

Copy and paste relevant sections from the manuscript (include quotes in quotation marks "like this" to indicate direct quotes from your manuscript), or elaborate on this item by providing additional information not in the ms, or briefly explain why the item is not applicable/relevant for your study

Not applicable. No changes were made.

7a) How sample size was determined

NPT: When applicable, details of whether and how the clustering by care provides or centers was addressed

7a-i) Describe whether and how expected attrition was taken into account when calculating the sample size

Describe whether and how expected attrition was taken into account when calculating the sample size.

	1	2	3	4	5	
subitem not at all important	○	○	○	○	○	essential

Does your paper address subitem 7a-i?

Copy and paste relevant sections from manuscript title (include quotes in quotation marks "like this" to indicate direct quotes from your manuscript), or elaborate on this item by providing additional information not in the ms, or briefly explain why the item is not applicable/relevant for your study

"The number of participants was limited by available resources (eg, activity bracelets, heart rate variability sensors, and technical support). The intervention length was limited partly by the estimated battery life of Mi Band activity bracelets (approximately 40-50 days without charging) and partly to avoid major public holidays that might affect participants' physical activity. The planned sample of 15 participants with 40 observation days would yield 600 observation days in total, which was similar to that of earlier factorial N-of-1 RCTs that included 8 participants for 62 days and 10 participants for 60 days [28,29]." Attrition was not accounted for.

7b) When applicable, explanation of any interim analyses and stopping guidelines

Does your paper address CONSORT subitem 7b? *

Copy and paste relevant sections from the manuscript (include quotes in quotation marks "like this" to indicate direct quotes from your manuscript), or elaborate on this item by providing additional information not in the ms, or briefly explain why the item is not applicable/relevant for your study

There were no stopping rules or interim analyses.

8a) Method used to generate the random allocation sequence

NPT: When applicable, how care providers were allocated to each trial group

Does your paper address CONSORT subitem 8a? *

Copy and paste relevant sections from the manuscript (include quotes in quotation marks "like this" to indicate direct quotes from your manuscript), or elaborate on this item by providing additional information not in the ms, or briefly explain why the item is not applicable/relevant for your study

"Each active study period tested the effects of one of the following conditions: (1) both dMI and biofeedback, (2) dMI alone, (3) biofeedback alone, or (4) a control period in which neither intervention was delivered. The 4 conditions were repeatedly block randomized to one of the 12 three-day active study periods using a computer-generated code. This led to each of the 4 conditions being assigned to three 3-day study periods overall. Randomizing the timing of repeated interventions helps avoid potential time-based confounders that might systematically coincide with intervention delivery [62]. A code for activating the individually randomized trial was printed and sealed in an opaque envelope. Each participant drew one of the envelopes at the baseline meeting with a researcher (JN) and entered this code into the app to initiate the trial procedure."

8b) Type of randomisation; details of any restriction (such as blocking and block size)

Does your paper address CONSORT subitem 8b? *

Copy and paste relevant sections from the manuscript (include quotes in quotation marks "like this" to indicate direct quotes from your manuscript), or elaborate on this item by providing additional information not in the ms, or briefly explain why the item is not applicable/relevant for your study

The 4 conditions were repeatedly block randomized to one of the 12 three-day active study periods using a computer-generated code. This led to each of the 4 conditions being assigned to three 3-day study periods overall.

9) Mechanism used to implement the random allocation sequence (such as sequentially numbered containers), describing any steps taken to conceal the sequence until interventions were assigned

Does your paper address CONSORT subitem 9? *

Copy and paste relevant sections from the manuscript (include quotes in quotation marks "like this" to indicate direct quotes from your manuscript), or elaborate on this item by providing additional information not in the ms, or briefly explain why the item is not applicable/relevant for your study

The 4 conditions were repeatedly block randomized to one of the 12 three-day active study periods using a computer-generated code. This led to each of the 4 conditions being assigned to three 3-day study periods overall. Randomizing the timing of repeated interventions helps avoid potential time-based confounders that might systematically coincide with intervention delivery [62]. A code for activating the individually randomized trial was printed and sealed in an opaque envelope.

10) Who generated the random allocation sequence, who enrolled participants, and who assigned participants to interventions

Does your paper address CONSORT subitem 10? *

Copy and paste relevant sections from the manuscript (include quotes in quotation marks "like this" to indicate direct quotes from your manuscript), or elaborate on this item by providing additional information not in the ms, or briefly explain why the item is not applicable/relevant for your study

"The 4 conditions were repeatedly block randomized to one of the 12 three-day active study periods using a computer-generated code. ... A code for activating the individually randomized trial was printed and sealed in an opaque envelope. Each participant drew one of the envelopes at the baseline meeting with a researcher (JN) and entered this code into the app to initiate the trial procedure."

11a) If done, who was blinded after assignment to interventions (for example, participants, care providers, those assessing outcomes) and how

NPT: Whether or not administering co-interventions were blinded to group assignment

11a-i) Specify who was blinded, and who wasn’t

Specify who was blinded, and who wasn’t. Usually, in web-based trials it is not possible to blind the participants [1, 3] (this should be clearly acknowledged), but it may be possible to blind outcome assessors, those doing data analysis or those administering co-interventions (if any).

	1	2	3	4	5	
subitem not at all important	○	○	○	○	○	essential

Does your paper address subitem 11a-i? *

Copy and paste relevant sections from the manuscript (include quotes in quotation marks "like this" to indicate direct quotes from your manuscript), or elaborate on this item by providing additional information not in the ms, or briefly explain why the item is not applicable/relevant for your study

As we aimed to test the impact of motivational features that require active cognitive engagement with the tasks [37], it was not possible to blind participants to the interventions they received on a given day. However, participants were not aware of the specific study hypotheses or analysis methods, and during the study, participants were blinded to the sequence in which intervention components would be delivered or available in the Precious app. Tests for blinding were not conducted.

11a-ii) Discuss e.g., whether participants knew which intervention was the “intervention of interest” and which one was the “comparator”

Informed consent procedures (4a-ii) can create biases and certain expectations - discuss e.g., whether participants knew which intervention was the “intervention of interest” and which one was the “comparator”.

	1	2	3	4	5	
subitem not at all important	○	○	○	○	○	essential

Does your paper address subitem 11a-ii?

Copy and paste relevant sections from the manuscript (include quotes in quotation marks "like this" to indicate direct quotes from your manuscript), or elaborate on this item by providing additional information not in the ms, or briefly explain why the item is not applicable/relevant for your study

As we aimed to test the impact of motivational features that require active cognitive engagement with the tasks [37], it was not possible to blind participants to the interventions they received on a given day. However, participants were not aware of the specific study hypotheses or analysis methods, and during the study, participants were blinded to the sequence in which intervention components would be delivered or available in the Precious app. Tests for blinding were not conducted.

11b) If relevant, description of the similarity of interventions

(this item is usually not relevant for ehealth trials as it refers to similarity of a placebo or sham intervention to a active medication/intervention)

Does your paper address CONSORT subitem 11b? *

Copy and paste relevant sections from the manuscript (include quotes in quotation marks "like this" to indicate direct quotes from your manuscript), or elaborate on this item by providing additional information not in the ms, or briefly explain why the item is not applicable/relevant for your study

Not applicable to this study.

12a) Statistical methods used to compare groups for primary and secondary outcomes

NPT: When applicable, details of whether and how the clustering by care providers or centers was addressed

Does your paper address CONSORT subitem 12a? *

Copy and paste relevant sections from the manuscript (include quotes in quotation marks "like this" to indicate direct quotes from your manuscript), or elaborate on this item by providing additional information not in the ms, or briefly explain why the item is not applicable/relevant for your study

There were no group comparisons in this trial as it was an N-of-1 RCT.

12a-i) Imputation techniques to deal with attrition / missing values

Imputation techniques to deal with attrition / missing values: Not all participants will use the intervention/comparator as intended and attrition is typically high in ehealth trials. Specify how participants who did not use the application or dropped out from the trial were treated in the statistical analysis (a complete case analysis is strongly discouraged, and simple imputation techniques such as LOCF may also be problematic [4]).

	1	2	3	4	5	
subitem not at all important	○	○	○	○	○	essential

Does your paper address subitem 12a-i? *

Copy and paste relevant sections from the manuscript (include quotes in quotation marks "like this" to indicate direct quotes from your manuscript), or elaborate on this item by providing additional information not in the ms, or briefly explain why the item is not applicable/relevant for your study

Missing values were not imputed as the missing completely at random test [71] found the study values not to be missing completely at random (χ225=85.3, P<.001) and common multiple-imputation methods cannot reliably handle this potential bias [72,73].

12b) Methods for additional analyses, such as subgroup analyses and adjusted analyses

Does your paper address CONSORT subitem 12b? *

Copy and paste relevant sections from the manuscript (include quotes in quotation marks "like this" to indicate direct quotes from your manuscript), or elaborate on this item by providing additional information not in the ms, or briefly explain why the item is not applicable/relevant for your study

Preliminary Analyses
The analyses were conducted using SPSS Statistics (version 24; IBM Corp). Steps and daily EMA scores were plotted to visualize temporal patterns, check for nonlinear patterns, and visually explore and compare individuals' scores as recommended in the studies by Bolger and Laurenceau [20], McDonald et al [32], and Manolov et al [67].
N-of-1 Analyses
Participants' time-series data sets were analyzed separately using dynamic regression [32,68] as this method can accommodate small sample sizes [69]. Dynamic regression modeling aims to capture the impact of past observations on an outcome by building autoregressive lag models and, therefore, can be used to account for regular cyclical patterns that may occur within an individual's physical activity levels over time [70]. To maximize statistical power, wash-out days were included in these analyses and treated as control days (as no interventions were delivered).
To investigate the effects of MI and biofeedback interventions on daily steps, a multistep procedure was followed. First, we examined linear and quadratic trends within the time series using curve estimation and retained any statistically significant time trends as predictors in subsequent models. Next, we examined cyclical patterns within the data by examining the day of the week as a predictor of daily steps. For participants for whom autocorrelation in steps was likely based on autocorrelation plots and the statistical threshold of $P<.05$, lagged step variables were created and used as controls.
Any variables that predicted steps in these initial models were retained for the final model, which in addition introduced 2 dichotomous intervention delivery variables (ie, delivery of dMI and delivery of biofeedback) as primary predictors of daily steps and perceived barriers and pain as control variables. This procedure was then repeated to examine the relationship between morning motivation and morning self-efficacy and participants' daily step totals.
Aggregated Analyses
To investigate the effects of the interventions on participants' steps, we used random intercept multilevel modeling on an aggregated data set that excluded wash-out days (465 possible observations; 409 after accounting for missing step data). First, a null model (model 1) was run to fit the grand mean for steps, provide a baseline for the model fit statistics, and assess the intraclass correlation coefficient (ICC). Time was then added as a fixed factor in model 2 and also as a random factor in model 3. In model 4, we added the day of the week (to control for weekly repeating patterns in steps) and within- and between-subject levels of pain or illness and perceived barriers (to control for factors that were not under the participants' control, which varied both within and between persons [20]). Model 5 introduced 2 dichotomous intervention delivery variables (ie, delivery of dMI and delivery of biofeedback) as the primary predictors of daily steps. Finally, in model 6, we examined whether including autocorrelation patterns improved model fit.
To examine the associations between morning motivation and morning self-efficacy and participants' steps, we used random intercept multilevel modeling on an aggregated data set that included wash-out days (600 possible observations; 530 after accounting for missing step data). We included wash-out days in these analyses as we did not expect carryover effects of the interventions to moderate the examined relationships between independent variables (ie, motivation and self-efficacy) and step counts. In these analyses, we followed the same 6-step modeling process described previously, but model 5 introduced within- and between-person measures of motivation or self-efficacy instead of intervention delivery variables
To facilitate interpretation of the intercept in all multilevel analyses, the scores were grand mean and person mean centered. Iterative improvements in model fit were assessed by conducting a chi-square test using the difference in deviance (−2 log likelihood) between successive models as the chi-square test value and the difference in df between successive models as the df for the chi-square test.

X26) REB/IRB Approval and Ethical Considerations [recommended as subheading under "Methods"] (not a CONSORT item)

X26-i) Comment on ethics committee approval

	1	2	3	4	5	
subitem not at all important	○	○	○	○	○	essential

Does your paper address subitem X26-i?

Copy and paste relevant sections from the manuscript (include quotes in quotation marks "like this" to indicate direct quotes from your manuscript), or elaborate on this item by providing additional information not in the ms, or briefly explain why the item is not applicable/relevant for your study

The University of Helsinki Ethical Review Board in the Humanities and Social and Behavioural Sciences granted a favorable decision for this study (Statement #: 3/2016).

x26-ii) Outline informed consent procedures

Outline informed consent procedures e.g., if consent was obtained offline or online (how? Checkbox, etc.?), and what information was provided (see 4a-ii). See [6] for some items to be included in informed consent documents.

	1	2	3	4	5	
subitem not at all important	○	○	○	○	○	essential

Does your paper address subitem X26-ii?

Copy and paste relevant sections from the manuscript (include quotes in quotation marks "like this" to indicate direct quotes from your manuscript), or elaborate on this item by providing additional information not in the ms, or briefly explain why the item is not applicable/relevant for your study

Participants were invited for a face-to-face intake session from October 2016 to November 2016 in which they were asked to read the study information sheet (which had also been sent to them by email) and could ask questions. Individuals wishing to be enrolled in the study then provided informed consent to participate and began the study.

X26-iii) Safety and security procedures

Safety and security procedures, incl. privacy considerations, and any steps taken to reduce the likelihood or detection of harm (e.g., education and training, availability of a hotline)

	1	2	3	4	5	
subitem not at all important	○	○	○	○	○	essential

Does your paper address subitem X26-iii?

Copy and paste relevant sections from the manuscript (include quotes in quotation marks "like this" to indicate direct quotes from your manuscript), or elaborate on this item by providing additional information not in the ms, or briefly explain why the item is not applicable/relevant for your study

There were no safety or security risks associated with this study. Participants could contact the research team at any time if some problems did occur however.

RESULTS

13a) For each group, the numbers of participants who were randomly assigned, received intended treatment, and were analysed for the primary outcome

NPT: The number of care providers or centers performing the intervention in each group and the number of patients treated by each care provider in each center

Does your paper address CONSORT subitem 13a? *

Copy and paste relevant sections from the manuscript (include quotes in quotation marks "like this" to indicate direct quotes from your manuscript), or elaborate on this item by providing additional information not in the ms, or briefly explain why the item is not applicable/relevant for your study

A total of 15 healthy adults (n=4, 27% male and n=11, 73% female) took part in the study, with ages ranging from 28 to 57 years (mean 42.33, SD 9.82 years). All participants reported wearing the activity bracelet continuously for the duration of the trial; however, some participants were missing step data because of technical errors, including participant 9, who had no step data, and participant 2, who had step data for only 52% (21/40) of the measurement days. This left a total of 88.3% (530/600) of usable data points.

13b) For each group, losses and exclusions after randomisation, together with reasons

Does your paper address CONSORT subitem 13b? (NOTE: Preferably, this is shown in a CONSORT flow diagram) *

Copy and paste relevant sections from the manuscript (include quotes in quotation marks "like this" to indicate direct quotes from your manuscript), or elaborate on this item by providing additional information not in the ms, or briefly explain why the item is not applicable/relevant for your study

A total of 15 healthy adults (n=4, 27% male and n=11, 73% female) took part in the study, with ages ranging from 28 to 57 years (mean 42.33, SD 9.82 years). All participants reported wearing the activity bracelet continuously for the duration of the trial; however, some participants were missing step data because of technical errors, including participant 9, who had no step data, and participant 2, who had step data for only 52% (21/40) of the measurement days. This left a total of 88.3% (530/600) of usable data points.

13b-i) Attrition diagram

Strongly recommended: An attrition diagram (e.g., proportion of participants still logging in or using the intervention/comparator in each group plotted over time, similar to a survival curve) or other figures or tables demonstrating usage/dose/engagement.

	1	2	3	4	5	
subitem not at all important	○	○	○	○	○	essential

Does your paper address subitem 13b-i?

Copy and paste relevant sections from the manuscript or cite the figure number if applicable (include quotes in quotation marks "like this" to indicate direct quotes from your manuscript), or elaborate on this item by providing additional information not in the ms, or briefly explain why the item is not applicable/relevant for your study

Not applicable as app content changed differently for each user, depending on the condition that day had been randomized to.

14a) Dates defining the periods of recruitment and follow-up

Does your paper address CONSORT subitem 14a? *

Copy and paste relevant sections from the manuscript (include quotes in quotation marks "like this" to indicate direct quotes from your manuscript), or elaborate on this item by providing additional information not in the ms, or briefly explain why the item is not applicable/relevant for your study

Participants were recruited from the general population using advertisements in the “Metro” newspaper of the Helsinki area, Finland, and a Facebook page and targeted advertisements in October 2016. The study lasted 40 days and all data collection was completed before the end of 2016.

14a-i) Indicate if critical “secular events” fell into the study period

Indicate if critical “secular events” fell into the study period, e.g., significant changes in Internet resources available or “changes in computer hardware or Internet delivery resources”

	1	2	3	4	5	
subitem not at all important	○	○	○	○	○	essential

Does your paper address subitem 14a-i?

Copy and paste relevant sections from the manuscript (include quotes in quotation marks "like this" to indicate direct quotes from your manuscript), or elaborate on this item by providing additional information not in the ms, or briefly explain why the item is not applicable/relevant for your study

Not applicable to this study

14b) Why the trial ended or was stopped (early)

Does your paper address CONSORT subitem 14b? *

Copy and paste relevant sections from the manuscript (include quotes in quotation marks "like this" to indicate direct quotes from your manuscript), or elaborate on this item by providing additional information not in the ms, or briefly explain why the item is not applicable/relevant for your study

Recruitment target was hit and the study ended as planned.

15) A table showing baseline demographic and clinical characteristics for each group

NPT: When applicable, a description of care providers (case volume, qualification, expertise, etc.) and centers (volume) in each group

Does your paper address CONSORT subitem 15? *

Copy and paste relevant sections from the manuscript (include quotes in quotation marks "like this" to indicate direct quotes from your manuscript), or elaborate on this item by providing additional information not in the ms, or briefly explain why the item is not applicable/relevant for your study

Table 1 contains this information

15-i) Report demographics associated with digital divide issues

In ehealth trials it is particularly important to report demographics associated with digital divide issues, such as age, education, gender, social-economic status, computer/Internet/ehealth literacy of the participants, if known.

	1	2	3	4	5	
subitem not at all important	○	○	○	○	○	essential

Does your paper address subitem 15-i? *

Copy and paste relevant sections from the manuscript (include quotes in quotation marks "like this" to indicate direct quotes from your manuscript), or elaborate on this item by providing additional information not in the ms, or briefly explain why the item is not applicable/relevant for your study

Table 1 contains this information

16) For each group, number of participants (denominator) included in each analysis and whether the analysis was by original assigned groups

16-i) Report multiple “denominators” and provide definitions

Report multiple “denominators” and provide definitions: Report N’s (and effect sizes) “across a range of study participation [and use] thresholds” [1], e.g., N exposed, N consented, N used more than x times, N used more than y weeks, N participants “used” the intervention/comparator at specific pre-defined time points of interest (in absolute and relative numbers per group). Always clearly define “use” of the intervention.

	1	2	3	4	5	
subitem not at all important	○	○	○	○	○	essential

Does your paper address subitem 16-i? *

Copy and paste relevant sections from the manuscript (include quotes in quotation marks "like this" to indicate direct quotes from your manuscript), or elaborate on this item by providing additional information not in the ms, or briefly explain why the item is not applicable/relevant for your study

Table 1 contains this information

16-ii) Primary analysis should be intent-to-treat

Primary analysis should be intent-to-treat, secondary analyses could include comparing only “users”, with the appropriate caveats that this is no longer a randomized sample (see 18-i).

	1	2	3	4	5	
subitem not at all important	○	○	○	○	○	essential

Does your paper address subitem 16-ii?

Copy and paste relevant sections from the manuscript (include quotes in quotation marks "like this" to indicate direct quotes from your manuscript), or elaborate on this item by providing additional information not in the ms, or briefly explain why the item is not applicable/relevant for your study

"This study conducted intention-to-treat analyses studying whether intervention availability is associated with increased step count. " "To maximize statistical power, wash-out days were included in these analyses and treated as control days (as no interventions were delivered)."

17a) For each primary and secondary outcome, results for each group, and the estimated effect size and its precision (such as 95% confidence interval)

Does your paper address CONSORT subitem 17a? *

Copy and paste relevant sections from the manuscript (include quotes in quotation marks "like this" to indicate direct quotes from your manuscript), or elaborate on this item by providing additional information not in the ms, or briefly explain why the item is not applicable/relevant for your study

Tables 3, 5, 9 & 10 contain this information

17a-i) Presentation of process outcomes such as metrics of use and intensity of use

In addition to primary/secondary (clinical) outcomes, the presentation of process outcomes such as metrics of use and intensity of use (dose, exposure) and their operational definitions is critical. This does not only refer to metrics of attrition (13-b) (often a binary variable), but also to more continuous exposure metrics such as “average session length”. These must be accompanied by a technical description how a metric like a “session” is defined (e.g., timeout after idle time) [1] (report under item 6a).

	1	2	3	4	5	
subitem not at all important	○	○	○	○	○	essential

Does your paper address subitem 17a-i?

Copy and paste relevant sections from the manuscript (include quotes in quotation marks "like this" to indicate direct quotes from your manuscript), or elaborate on this item by providing additional information not in the ms, or briefly explain why the item is not applicable/relevant for your study

Table 1 includes this information

17b) For binary outcomes, presentation of both absolute and relative effect sizes is recommended

Does your paper address CONSORT subitem 17b? *

Copy and paste relevant sections from the manuscript (include quotes in quotation marks "like this" to indicate direct quotes from your manuscript), or elaborate on this item by providing additional information not in the ms, or briefly explain why the item is not applicable/relevant for your study

Not applicable for this study

18) Results of any other analyses performed, including subgroup analyses and adjusted analyses, distinguishing pre-specified from exploratory

Does your paper address CONSORT subitem 18? *

Copy and paste relevant sections from the manuscript (include quotes in quotation marks "like this" to indicate direct quotes from your manuscript), or elaborate on this item by providing additional information not in the ms, or briefly explain why the item is not applicable/relevant for your study

Not applicable for this study

18-i) Subgroup analysis of comparing only users

A subgroup analysis of comparing only users is not uncommon in ehealth trials, but if done, it must be stressed that this is a self-selected sample and no longer an unbiased sample from a randomized trial (see 16-iii).

	1	2	3	4	5	
subitem not at all important	○	○	○	○	○	essential

Does your paper address subitem 18-i?

Copy and paste relevant sections from the manuscript (include quotes in quotation marks "like this" to indicate direct quotes from your manuscript), or elaborate on this item by providing additional information not in the ms, or briefly explain why the item is not applicable/relevant for your study

"Owing to the pilot nature of the study, the relatively low number of days of engagement with the service, and the spillover of the biofeedback measurements, no per-protocol analyses were conducted. "

19) All important harms or unintended effects in each group

(for specific guidance see CONSORT for harms)

Does your paper address CONSORT subitem 19? *

Copy and paste relevant sections from the manuscript (include quotes in quotation marks "like this" to indicate direct quotes from your manuscript), or elaborate on this item by providing additional information not in the ms, or briefly explain why the item is not applicable/relevant for your study

No harms were reported during the trial

19-i) Include privacy breaches, technical problems

Include privacy breaches, technical problems. This does not only include physical “harm” to participants, but also incidents such as perceived or real privacy breaches [1], technical problems, and other unexpected/unintended incidents. “Unintended effects” also includes unintended positive effects [2].

	1	2	3	4	5	
subitem not at all important	○	○	○	○	○	essential

Does your paper address subitem 19-i?

Copy and paste relevant sections from the manuscript (include quotes in quotation marks "like this" to indicate direct quotes from your manuscript), or elaborate on this item by providing additional information not in the ms, or briefly explain why the item is not applicable/relevant for your study

No privacy breaches were reported. "some participants were missing step data because of technical errors, including participant 9, who had no step data, and participant 2, who had step data for only 52% (21/40) of the measurement days." and "Unforeseen technical problems prevented 13% (2/15) of the participants from accessing their biofeedback reports during the trial (details in Table 1 and Table S2 in Multimedia Appendix 2). " and "Delivery of the dMI elements of the Precious app was deemed acceptable and feasible, whereas the biofeedback interventions faced some technical and practical challenges. As 27% (4/15) of the participants missed some biofeedback measurement days, smartphone notifications alone seem to have been an insufficient nudge to start the measurement. "

19-ii) Include qualitative feedback from participants or observations from staff/researchers

Include qualitative feedback from participants or observations from staff/researchers, if available, on strengths and shortcomings of the application, especially if they point to unintended/unexpected effects or uses. This includes (if available) reasons for why people did or did not use the application as intended by the developers.

	1	2	3	4	5	
subitem not at all important	○	○	○	○	○	essential

Does your paper address subitem 19-ii?

Copy and paste relevant sections from the manuscript (include quotes in quotation marks "like this" to indicate direct quotes from your manuscript), or elaborate on this item by providing additional information not in the ms, or briefly explain why the item is not applicable/relevant for your study

"Interestingly, anecdotal feedback in the follow-up interviews (not reported in this study) revealed that the participants may have actively varied their activity levels on biofeedback measurement days to receive a comprehensive picture of their well-being. Thus, the biofeedback measurements may have encouraged participants to even decrease their steps to receive a baseline reading on a recovery day."

DISCUSSION

22) Interpretation consistent with results, balancing benefits and harms, and considering other relevant evidence

NPT: In addition, take into account the choice of the comparator, lack of or partial blinding, and unequal expertise of care providers or centers in each group

22-i) Restate study questions and summarize the answers suggested by the data, starting with primary outcomes and process outcomes (use)

Restate study questions and summarize the answers suggested by the data, starting with primary outcomes and process outcomes (use).

	1	2	3	4	5	
subitem not at all important	○	○	○	○	○	essential

Does your paper address subitem 22-i? *

Copy and paste relevant sections from the manuscript (include quotes in quotation marks "like this" to indicate direct quotes from your manuscript), or elaborate on this item by providing additional information not in the ms, or briefly explain why the item is not applicable/relevant for your study

Yes, this is covered in the manuscript.

22-ii) Highlight unanswered new questions, suggest future research

Highlight unanswered new questions, suggest future research.

	1	2	3	4	5	
subitem not at all important	○	○	○	○	○	essential

Does your paper address subitem 22-ii?

Copy and paste relevant sections from the manuscript (include quotes in quotation marks "like this" to indicate direct quotes from your manuscript), or elaborate on this item by providing additional information not in the ms, or briefly explain why the item is not applicable/relevant for your study

"Future studies are needed to investigate how digital interventions could approach the effectiveness of personal contact, exploring, for instance, the amount of contact [80] or the depth of engagement with intervention content [37]. " and "Future studies could focus on detecting the effects of motivation on planning and follow the enactment of these plans." and "future tests of the Precious system would need to be conducted using a research design that allows for the use of interactive motivational content. One option is the changing criterion design [82], in which a new intervention feature is introduced when participants reach a certain level in the outcome variable." and "Future studies could explore whether the days in which participants actively engaged with the intervention materials are associated with changes in behavior as behavior change interventions are most effective for people who actively use the available BCTs [85-87]."

20) Trial limitations, addressing sources of potential bias, imprecision, and, if relevant, multiplicity of analyses

20-i) Typical limitations in ehealth trials

Typical limitations in ehealth trials: Participants in ehealth trials are rarely blinded. Ehealth trials often look at a multiplicity of outcomes, increasing risk for a Type I error. Discuss biases due to non-use of the intervention/usability issues, biases through informed consent procedures, unexpected events.

	1	2	3	4	5	
subitem not at all important	○	○	○	○	○	essential

Does your paper address subitem 20-i? *

Copy and paste relevant sections from the manuscript (include quotes in quotation marks "like this" to indicate direct quotes from your manuscript), or elaborate on this item by providing additional information not in the ms, or briefly explain why the item is not applicable/relevant for your study

"Limitations
As 27% (4/15) of the participants missed some biofeedback measurement days, smartphone notifications alone seem to have been an insufficient nudge to start the measurement. As N-of-1 RCTs are very sensitive to the accurate timing of interventions [61], future studies using app-controlled intervention delivery need to ensure that notifications are received on time, possibly using sound and vibration alarms in addition to text-based notifications.
This study conducted intention-to-treat analyses studying whether intervention availability is associated with increased step count. Participants typically only engaged with the dMI interventions on the first day they were available. Owing to the pilot nature of the study, the relatively low number of days of engagement with the service, and the spillover of the biofeedback measurements, no per-protocol analyses were conducted. Future studies could explore whether the days in which participants actively engaged with the intervention materials are associated with changes in behavior as behavior change interventions are most effective for people who actively use the available BCTs [85-87].
To minimize participant burden and the risk of confounding motivational effects, all EMA questions were single items [88]. However, single items offer no same-day reference points for imputing missing values. As participants showed high adherence to the twice-a-day EMA measurement [89], future studies could consider assessing more motivational variables that, for example, distinguish qualitatively different motivations from SDT [49].
This study used a factorial design to test 2 different interventions in a short field trial. The duration of this trial was limited to 40 days, primarily because of the estimated battery life of the accelerometers used to collect step data. This means that the study was underpowered to assess the interaction effects of the 2 interventions and, according to recent evidence [90], perhaps even underpowered to study the effects of single interventions in which longer time-series data are needed."

21) Generalisability (external validity, applicability) of the trial findings

NPT: External validity of the trial findings according to the intervention, comparators, patients, and care providers or centers involved in the trial

21-i) Generalizability to other populations

Generalizability to other populations: In particular, discuss generalizability to a general Internet population, outside of a RCT setting, and general patient population, including applicability of the study results for other organizations

	1	2	3	4	5	
subitem not at all important	○	○	○	○	○	essential

Does your paper address subitem 21-i?

Copy and paste relevant sections from the manuscript (include quotes in quotation marks "like this" to indicate direct quotes from your manuscript), or elaborate on this item by providing additional information not in the ms, or briefly explain why the item is not applicable/relevant for your study

Your answer

21-ii) Discuss if there were elements in the RCT that would be different in a routine application setting

Discuss if there were elements in the RCT that would be different in a routine application setting (e.g., prompts/reminders, more human involvement, training sessions or other co-interventions) and what impact the omission of these elements could have on use, adoption, or outcomes if the intervention is applied outside of a RCT setting.

	1	2	3	4	5	
subitem not at all important	○	○	○	○	○	essential

Does your paper address subitem 21-ii?

Copy and paste relevant sections from the manuscript (include quotes in quotation marks "like this" to indicate direct quotes from your manuscript), or elaborate on this item by providing additional information not in the ms, or briefly explain why the item is not applicable/relevant for your study

Your answer

OTHER INFORMATION

23) Registration number and name of trial registry

Does your paper address CONSORT subitem 23? *

Copy and paste relevant sections from the manuscript (include quotes in quotation marks "like this" to indicate direct quotes from your manuscript), or elaborate on this item by providing additional information not in the ms, or briefly explain why the item is not applicable/relevant for your study

The trial was not formally preregistered as conventions for the registration of factorial N-of-1 experimental studies had not been established before this study's commencement in 2016.

24) Where the full trial protocol can be accessed, if available

Does your paper address CONSORT subitem 24? *

Cite a Multimedia Appendix, other reference, or copy and paste relevant sections from the manuscript (include quotes in quotation marks "like this" to indicate direct quotes from your manuscript), or elaborate on this item by providing additional information not in the ms, or briefly explain why the item is not applicable/relevant for your study

a dated publicly available version of the study protocol was published just after the start of data collection and before any data analyses [60].

25) Sources of funding and other support (such as supply of drugs), role of funders

Does your paper address CONSORT subitem 25? *

Copy and paste relevant sections from the manuscript (include quotes in quotation marks "like this" to indicate direct quotes from your manuscript), or elaborate on this item by providing additional information not in the ms, or briefly explain why the item is not applicable/relevant for your study

The research leading to these results has received funding from the European Community Seventh Framework Programme for the Precious project under grant agreement 611366, the Doctoral Programme in Social Sciences of the University of Helsinki, the KAUTE Foundation, and the Yrjö Jahnsson Foundation.

X27) Conflicts of Interest (not a CONSORT item)

X27-i) State the relation of the study team towards the system being evaluated

In addition to the usual declaration of interests (financial or otherwise), also state the relation of the study team towards the system being evaluated, i.e., state if the authors/evaluators are distinct from or identical with the developers/sponsors of the intervention.

	1	2	3	4	5	
subitem not at all important	○	○	○	○	○	essential

Does your paper address subitem X27-i?

Copy and paste relevant sections from the manuscript (include quotes in quotation marks "like this" to indicate direct quotes from your manuscript), or elaborate on this item by providing additional information not in the ms, or briefly explain why the item is not applicable/relevant for your study

Your answer

About the CONSORT EHEALTH checklist

As a result of using this checklist, did you make changes in your manuscript? *

- ◯ yes, major changes
- ◉ yes, minor changes
- ◯ no

What were the most important changes you made as a result of using this checklist?

Your answer

How much time did you spend on going through the checklist INCLUDING making changes in your manuscript *

2 hours and 0 minutes = too damn long

As a result of using this checklist, do you think your manuscript has improved? *

- ◯ yes
- ◉ no
- ◯ Other:

Would you like to become involved in the CONSORT EHEALTH group?

This would involve for example becoming involved in participating in a workshop and writing an "Explanation and Elaboration" document

- ○ yes
- ◉ no
- ○ Other:

Clear selection

Any other comments or questions on CONSORT EHEALTH

Why require minimum length on questions?

STOP - Save this form as PDF before you click submit

To generate a record that you filled in this form, we recommend to generate a PDF of this page (on a Mac, simply select "print" and then select "print as PDF") before you submit it.

When you submit your (revised) paper to JMIR, please upload the PDF as supplementary file.

Don't worry if some text in the textboxes is cut off, as we still have the complete information in our database. Thank you!

Final step: Click submit !

Click submit so we have your answers in our database!

Submit Clear form

Never submit passwords through Google Forms.

This content is neither created nor endorsed by Google. Report Abuse - Terms of Service - Privacy Policy

Google Forms
